# Novel Selective Estrogen Receptor Ligand Conjugates Incorporating Endoxifen-Combretastatin and Cyclofenil-Combretastatin Hybrid Scaffolds: Synthesis and Biochemical Evaluation

**DOI:** 10.3390/molecules22091440

**Published:** 2017-08-31

**Authors:** Patrick M. Kelly, Niall O. Keely, Sandra A. Bright, Bassem Yassin, Gloria Ana, Darren Fayne, Daniela M. Zisterer, Mary J. Meegan

**Affiliations:** 1 School of Pharmacy and Pharmaceutical Sciences, Trinity Biomedical Sciences Institute, 152-160 Pearse Street, Trinity College Dublin, Dublin 2, Ireland; kellyp9@tcd.ie (P.M.K.); anag@tcd.ie (G.A.); 2 School of Pharmacy and Pharmaceutical Sciences, Trinity College Dublin, Dublin 2, Ireland; nkeely@tcd.ie (N.O.K.); bassem76@yahoo.com (B.Y.); 3 School of Biochemistry and Immunology, Trinity Biomedical Sciences Institute, 152-160 Pearse Street, Trinity College Dublin, Dublin 2, Ireland; brights@tcd.ie (S.A.B.); FAYNED@tcd.ie (D.F.); dzistrer@tcd.ie (D.M.Z.)

**Keywords:** tumour targeting conjugates, selective estrogen receptor modulators, combretastatin A-4(CA-4), endoxifen, cyclofenil, estrogen receptor ligands, hormone-dependent breast cancer, apoptosis

## Abstract

Nuclear receptors such as the estrogen receptors (ERα and ERβ) modulate the effects of the estrogen hormones and are important targets for design of innovative chemotherapeutic agents for diseases such as breast cancer and osteoporosis. Conjugate and bifunctional compounds which incorporate an ER ligand offer a useful method of delivering cytotoxic drugs to tissue sites such as breast cancers which express ERs. A series of novel conjugate molecules incorporating both the ER ligands endoxifen and cyclofenil-endoxifen hybrids covalently linked to the antimitotic and tubulin targeting agent combretastatin A-4 were synthesised and evaluated as ER ligands. A number of these compounds demonstrated pro-apoptotic effects, with potent antiproliferative activity in ER-positive MCF-7 breast cancer cell lines and low cytotoxicity. These conjugates displayed binding affinity towards ERα and ERβ isoforms at nanomolar concentrations e.g., the cyclofenil-amide compound **13e** is a promising lead compound of a clinically relevant ER conjugate with IC_50_ in MCF-7 cells of 187 nM, and binding affinity to ERα (IC_50_ = 19 nM) and ERβ (IC_50_ = 229 nM) while the endoxifen conjugate **16b** demonstrates antiproliferative activity in MCF-7 cells (IC_50_ = 5.7 nM) and binding affinity to ERα (IC_50_ = 15 nM) and ERβ (IC_50_ = 115 nM). The ER binding effects are rationalised in a molecular modelling study in which the disruption of the ER helix-12 in the presence of compounds **11e**, **13e** and **16b** is presented These conjugate compounds have potential application for further development as antineoplastic agents in the treatment of ER positive breast cancers.

## 1. Introduction

Breast cancer is the most common cancer in women worldwide, affecting one in eight women and representing a significant cause of cancer death in women. Incidence rates are increasing steadily, with nearly 1.7 million new cases diagnosed in 2012 worldwide [1]. The majority of early stage breast cancers are hormone-dependent and patient prognosis is good. However, when the breast cancer is or becomes hormone-independent then prognosis is poor. About 5% of breast cancers, denoted BRCA-1 and BRCA-2, are considered hereditary [2]. The two nuclear estrogen receptors (ERα and ERβ) mediate the biological effects of the estrogen hormones [3,4]. These receptors are widely distributed in the body and are attractive therapeutic targets for diseases such as breast cancer and osteoporosis [5,6]. These receptors differ in their tissue distribution and in their ability to bind ligands. ERα is mainly found in uterus, bone, cardiovascular tissue and liver, and is the predominant receptor expressed in breast tumours [7]. ERα is also recognised to be mainly responsible for the effects of estrogen in hormonal replacement therapy. ERβ is expressed in many tissues and is the predominant ER in vascular endothelium, bone and male prostate tissues. The role of ERβ in the progression of breast cancer is still under investigation; ERβ expression has been reported to have a potentially protective effect on ERα promoted hyperproliferation [8,9,10,11,12]. The two ER subtypes differ significantly in size: 595 amino acids in ERα compared to 485 amino acids in ERβ. The conservation of amino acid sequence in the ligand binding sites of ERα and ERβ is only 59%, with the most notable differences in the ligand binding pocket (LBP) being replacement of Met412 and Leu384 in ERα with Ile and Met in ERβ [13,14,15]. Approaches to the discovery of selective estrogen receptor modulators (SERMs) rely on ER binding and cell-based estrogen response element-driven assays to identify compounds that are osteoprotective and antiproliferative in breast and uterine tissue. Estrogens are known to have tissue selective effects, and there is considerable interest in the therapeutic use of SERMs. These compounds act as ER antagonists in some tissues, e.g., uterus and breast, while functioning as ER agonists in the cardiovascular system, bone and brain.

**Figure 1:**
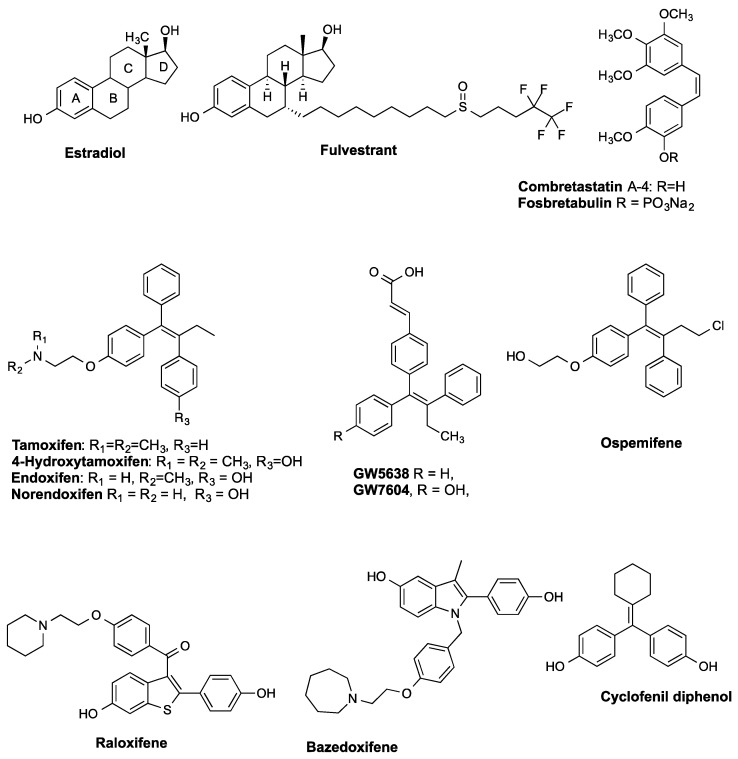
Estradiol, selected ER antagonists and combretastatin A-4.

A number of SERMs are currently in clinical use or clinical trials [16], including raloxifene [17] and tamoxifen [18,19], which are in widespread use for the prevention of osteoporosis and treatment of hormone-dependent breast cancer, respectively [4,20] (Figure 1). The indole-containing compound bazedoxifene [21] is also available for the treatment of osteoporosis and hot flushes in postmenopausal women, while ospemifene is approved for vaginal dryness [22]. Use of tamoxifen has been linked to increased risk of thrombosis and endometrial cancer. Also as the disease progresses, the effectiveness of drugs such as tamoxifen decreases as the tumours can become more resistant and less hormone dependent. The clinical success of tamoxifen and raloxifene has driven the search for new SERMs with applications as multifunctional drugs. Selective estrogen receptor downregulators (SERDs) such as fulvestrant reduce ERα protein levels and block ER activity. Fulvestrant is in clinical use for the treatment of postmenopausal ER positive breast cancer (locally advanced or metastatic) and is indicated for disease progression or relapse on adjuvant anti-estrogen therapy [23] (Figure 1). The acrylic acid GW5638 and the related hydroxylated GW7604, are identified as SERDs with similar core ligand triarylethylene structure to tamoxifen, but the basic side chain is replaced by an acrylic acid. GW5638 demonstrates agonist effects in bone but acts as an antagonist in breast tumours [24,25] (Figure 1).

Many approaches have been investigated to improve the selectivity of drugs used for chemotherapy by targeting tumour cells and associated receptors. The metabolites of tamoxifen, e.g., 4-hydroxytamoxifen, endoxifen and norendoxifen (Figure 1) possess potent antiestrogen activity [26]. Endoxifen is a potent antiestrogen, with strong ER affinity, degrades ER and interestingly demonstrates aromatase inhibitory activity [27,28,29]. In this research, the endoxifen molecule provides the ER targeting scaffold to be synthesised having selectivity to target the ER over-expressed in ER positive breast cancer cells, such as MCF-7 cells. The use of the endoxifen scaffold in the novel conjugate design should allow the selectivity to deliver a cytotoxic agent directly to the tumour site. Previous studies by Burke [30] and Keely [31] have demonstrated the use of the antiestrogen endoxifen for the conjugation of estradiol and cytotoxic drugs such as doxorubicin. The high affinity nonsteroidal ER ligand cyclofenil diphenol (F6060) [32,33] is also selected in the present work as an ER targeting core ligand for development of a novel conjugate design. Cyclofenil diphenol (F6060) and its derivatives have high binding affinity for ER, with mixed agonist-antagonist activity typical for a SERM such as tamoxifen or raloxifene [33]. F6060 and analogues have been shown to inhibit proteoglycan synthesis [34], similar to tamoxifen. Seo et al. reported good ER-subtype binding and selectivity in a number of cyclofenil analogues [33].

Typical conjugates are bifunctional molecules containing covalently linked ligands or pharmacophores which are designed to produce selectivity in targeting the intracellular ER [35,36]. The objectives of the conjugate design investigated in the current research are to produce conjugates capable of the delivery of cytotoxic agents to the ER positive breast cancer tumour cell, and to increase the selectivity of these cytotoxic agents which should result in less toxicity and increased efficacy. We also wished to produce ER antagonists through inclusion of the additional, bulky linker-cytotoxic agent moiety of the conjugate structure and with the possibility of achieving a dual-action activity i.e., ER antagonism and antimitotic activity.

ER ligand conjugates of cytotoxic agents, photodynamic therapeutic agents and radioligands which deliver cytotoxic agents have been reported [31,37]. We have previously reported stable conjugates of endoxifen (a tamoxifen metabolite and ER antagonist) with DNA alkylating agents, aromatase inhibitors, COX2 inhibitors and antitubulin compounds which demonstrate antiproliferative and ER binding effects [38]. We have also reported related conjugates based on the 2-arylindole scaffold structure which selectively target the ER in hormone dependent breast cancers [39]. Many previously reported ER conjugates have included ER agonist ligands such as estradiol as the ER ligand component [37,40]. However, in the present work we have incorporated ER antagonistic ligands such as endoxifen [27] and the novel cyclofenil based ER antagonist in the designed structures.

Combretastatin A-4 (CA-4) (Figure 1), a potent antimitotic agent isolated from the bark of the South African tree *Combretum caffrum*. It exhibits potent antitubulin effects by binding to tubulin at the colchicine binding site. CA-4 demonstrates cytotoxicity against a wide range of human cancer cell lines, including those that are multi-drug resistant [41,42,43]. However, because of poor water solubility of CA-4, a more water-soluble combretastatin A4-phosphate CA-4P (fosbretabulin, Figure 1) was synthesized and is currently under investigation in combination with pazopanib in a Phase 1b and Phase II study for the treatment of advanced recurrent ovarian cancer [44]. Many synthetic analogues of combretastatin A4 have been developed [45]. Among the various hybrids and conjugates of combretastatin A-4 which have been reported are hydrophobic combretastatin A-4 conjugated with hydrophilic irinotecan to form amphiphilic molecules (in which an azobenzene bond imparts hypoxia sensitivity) which can self assembly into nanoparticles for breast cancer synergistic therapy [46]. High-molecular weight conjugates of combretastatins with polyethylene glycol have been reported (comprising a block copolymer of a polyethylene glycol moiety and a polymer moiety having two or more carboxylic acid groups), in which a carboxylic acid group of the polymer moiety is linked to a hydroxyl group of combretastatins via an ester [47]. Combretastatin A-4 conjugated anti-angiogenic micellar drug delivery systems using dendron-polymer conjugates are also reported [48]. CA-4 analogues have been introduced onto steroid scaffolds to explore proapoptotic effects [49].

In the present work, ER antagonistic ligands such as endoxifen and cyclofenil related compounds are chosen as the targeting mechanism for the conjugate, with the objective of designing effective antiproliferative compounds for in vitro evaluation. The introduction of steric hindrance provided by the combretastatin CA-4 amide fragment is now investigated to determine if this modification enhances the ER antagonistic effects of the endoxifen and cyclofenil conjugates in the ER positive MCF-7 cells, possibly by interference with Helix-12 of the ER. To determine the influence of ER ligand structural modifications of the conjugate on antiproliferative activity in ER positive MCF-7 breast cancer cells, the following ER ligand conjugate structural types will be investigated:

1. Endoxifen—combretastatin-related acrylic acid conjugates **3a**–**3k**
2. Endoxifen—combretastatin-related modified acrylic acid conjugates **5a**–**5d**
3. Cyclofenil—combretastatin-related acrylic acid conjugates **13a**–**13e**
4. Endoxifen—combretastatin ester type conjugates **16a**–**16c**

## 2. Results and Discussion

### 2.1. Synthesis of Conjugates

**Scheme 1:**
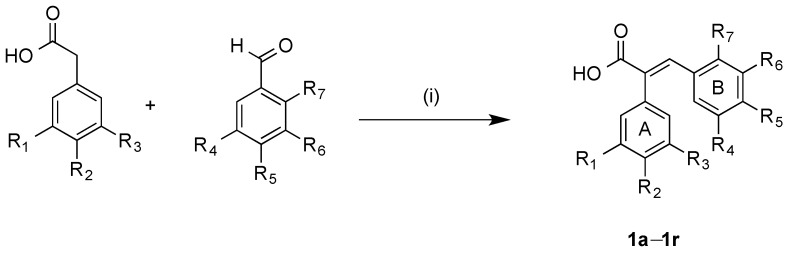
Synthesis of acrylic acids **1a**–**1r**: *Reagents and conditions*: (**i**) (CH_3_CO)_2_O, Et_3_N, microwaves, 100 °C, 0.5 h, followed by conc HCl.

For this study the required combretastatin related acrylic acids **1a**–**1r** (Table 1) were prepared using the Perkin condensation reaction which is an efficient synthetic route for novel combretastatin acrylic acid analogues from a variety of aldehyde and phenylacetic acid starting materials as shown in Scheme 1 [50]. The carboxylic acid located on the ethylene products will facilitate the conjugate formation with the ER ligands via coupling reactions to afford the required ester or amide linking system. Selection of the series of acrylic acids for synthesis was initially based on the requirement for the 3,4,5-trimethoxyphenyl substitution for ring A of CA4; however, other structurally related substitutions were also used as shown in Scheme 1. The panel of novel and previously reported acrylic acid combretastatin analogues **1a**–**1r** were synthesised via the Perkin reaction using both the reflux and microwave methods [51]. In all cases studied the yields for the microwave method were superior to those of the conventional technique method with shorter reaction time (<30 min) and with exclusive formation of the desired *cis* isomer. 2-(3,5-Dimethoxyphenyl)acetic acid and 2-(3-hydroxy-4-methoxyphenyl)acetic acid were prepared as described in the Supplementary information. Combretastatin CA-4 was used as a standard reference and was prepared by the Wittig reaction sequence [52] or by decarboxylation of **1l** [50].

Endoxifen was chosen as a suitable ER-ligand scaffold due to its high affinity ER-binding properties. Additionally, the secondary amine group present on the basic side chain can undergo amide type coupling reactions to synthesise the prototype conjugated compounds. The OTBDMS protected endoxifen ligand **2a** was prepared in a multistep route as we previously reported via the McMurry reaction which is a low valent titanium mediated crossed coupling of substituted benzophenones and ketones [31].

The McMurry reaction has been the route of choice for the preparation of the triarylethylene scaffold as it commonly leads to favourable *E*:*Z* isomer ratios. [53] The *E:Z* isomeric ratio for **2a** is calculated as 1:1.3 based on the integral of the signals of the chemical shifts assigned to the OCH_2_ and NCH_2_ signals for the protons of the basic side chain in the isomeric mixtures [54]. However, 4-hydroxytamoxifen and endoxifen and related 4-hydroxysubstituted triarylethylenes undergo *E*/*Z* isomerisation under physiological conditions, and have little effect on ER activity [55,56,57]. Therefore, the *E/Z* isomer mixture of **2a** obtained in the present work was used without further separation in the formation of the subsequent conjugates. Deprotection of **2a** affords endoxifen.

**Scheme 2:**
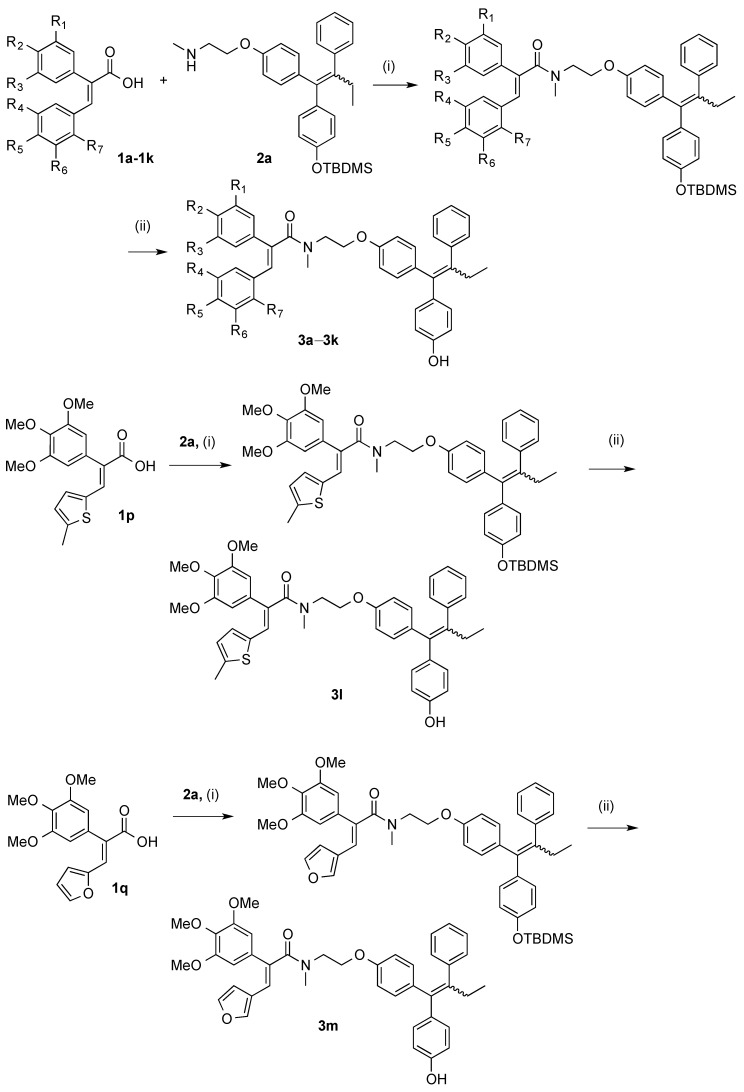
Synthesis of combretastatin-endoxifen conjugates **3a**–**3m**. *Reagents and Conditions*: ^a^ (i) DCC, HOBt, CH_2_Cl_2_, 24 h, 20 °C; (ii)TBAF, THF, 0.5 h, 20 °C; ^b^ (i) EDC, HOBt, CH_2_Cl_2_, 24 h, 20 °C; (ii) TBAF, THF, 0.5 h, 20 °C.

**Scheme 3:**
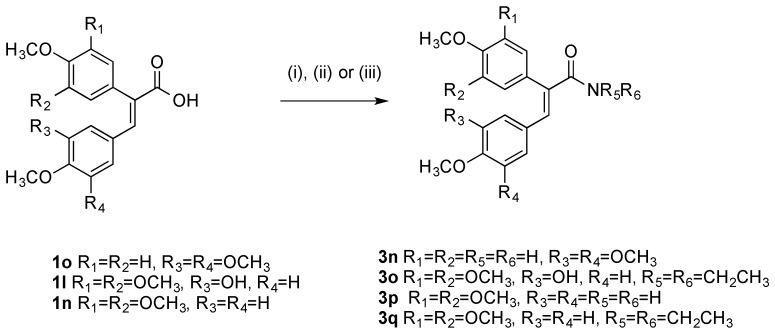
Synthesis of combretastatin-amides **3n**–**3q**: *Reagents and conditions*: **3o**, **3q** (**i**) Mukaiyama’s reagent, Et_3_N, DCM, 3 h, 20 °C; **3n** (**ii**) (COCl)_2_, DMF, DCM, 1 2h,20 °C, followed by NH_4_OH; **3p** (**iii**) SOCl_2_, DCM, Et_3_N, 2 h followed by NH_4_OH, 12 h, 20 °C.

The acrylic acid combretastatin analogues **1a**–**1j**, **1m**, **1p**, **1q** were directly coupled to the silyl-protected endoxifen analogue **2a** to afford the conjugates **3a**–**3m** (Scheme 2, Table 2). The initial coupling procedure investigated DCC as the coupling agent for the synthesis of this series of conjugates. Equimolar amounts of the acrylic acid, amine **2a** DCC and HOBt were reacted and the reaction was monitored via TLC. The resulting silyl-protected conjugates were treated with TBAF to afford the direct amide conjugates **3a**–**3m** in high yields as ~1:1 (*E/Z*)-isomeric mixtures, Table 2. The isomeric ratios were calculated based on those of the endoxifen starting material and confirmed by integration of appropriate signals in the ^1^H-NMR spectra. The presence of rotamers also resulted in complex spectra. EDC was also used as the coupling reagent for the synthesis of conjugates **3a**–**3k**. The related amide compounds **3n**, **3o***,* **3p** and **3q** were prepared for biochemical evaluation by reaction of the acrylic acids **1l**, **1n** and **1p** with ammonium acetate and diethylamine respectively (Scheme 3).

**Scheme 4:**
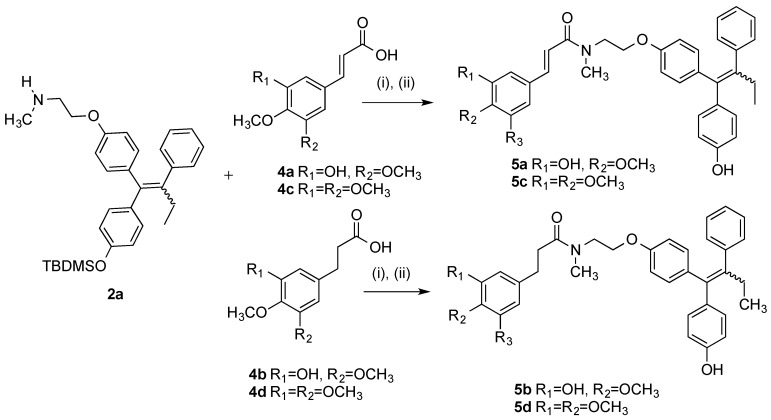
Synthesis of cinnamic and propanoic acid-combretastatin hybrid conjugates **5a**–**5d**. *Reagents and conditions*: (**i**) DCC or EDC, HOBt, CH_2_Cl_2_, 24 h, 20 °C; (**ii**) TBAF, THF, 0.5–24 h, 20 °C.

A further objective of this project was to prepare conjugates of endoxifen with cinnamic and phenylpropanoic acids related in structure to A and B rings of CA4. For this modified structure the substituent ring would now be either the 3,4,5-trimethoxy ring (A ring) or the 3-hydroxy-4-methoxy-ring (B ring). Both rings (A and B) have been shown to be relevant for the antitubulin CA4 activity [45]. The cinnamic acids **4a** and **4c** were prepared by reaction of the appropriate aryl aldehyde with malonic acid under microwave conditions [58]; subsequent reduction via a palladium/C hydrogenation afforded the 3-phenylpropanoic acids **4b** and **4d** [59] with yields in excess of 80%. (See Supplementary information). The compounds **4a**–**4d** were then used to synthesise the required endoxifen conjugates **5a**–**5d** via EDC or DCC and HOBt coupling reactions; the intermediate silyl-protected conjugates were deprotected with TBAF affording the desired amide conjugates in high yields (90–95%) as ~1:1 (*E/Z*)-isomeric mixtures, (Scheme 4).

In the present study, the cyclofenil analogues chosen for synthesis retain the cycloalkyl group of the cyclofenil parent structure, while in addition, they include the basic side chain moiety of the endoxifen parent structure. Unlike the previous synthesis of the triarylethylene scaffold for the endoxifen analogues, the use of a cyclic ketone eliminates any issue of *E*- and *Z*- isomers in the products. It was envisioned that the elimination of the *E/Z* isomers could lead to a simpler purification step and would result in a less complex NMR spectra.

**Scheme 5:**
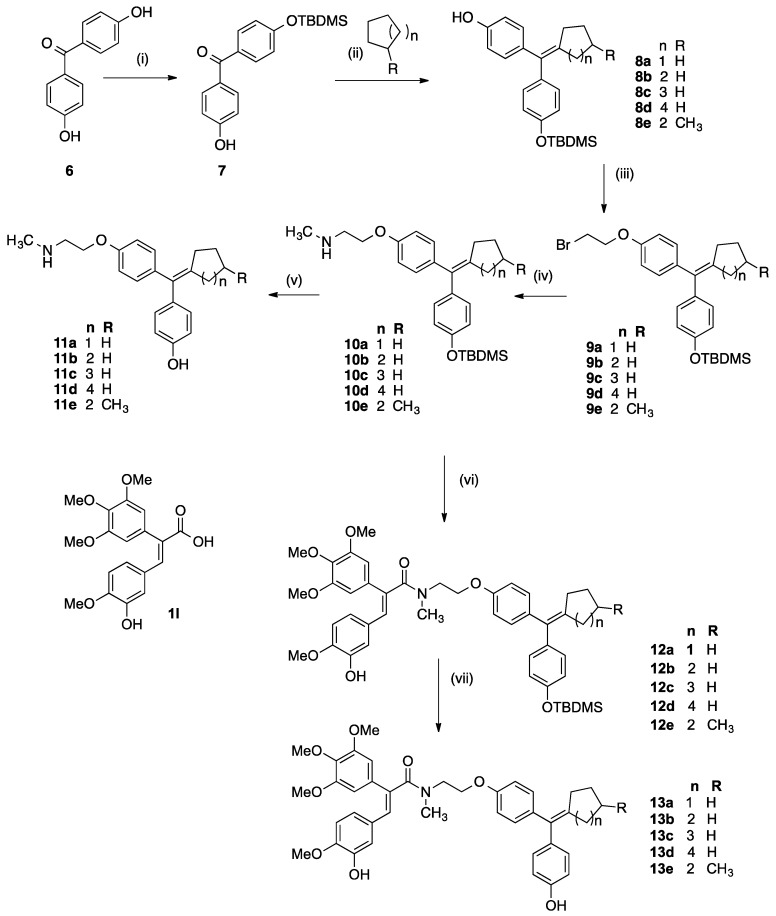
Synthesis of cyclofenil-combretastatin hybrid conjugates **13a**–**13e**. *Reagents and conditions*: (**i**) TBDMSCl, imidazole, DMF; (**ii**) Zn, TiCl_4_; (**iii**) Br_2_(CH_2_)_2_, NaOH(aq), nBu)4NHSO_3_, CH_2_Cl_2_; (**iv**) CH_3_NH_2_, sealed tube, 60 °C; (**v**) TBAF, THF; (**vi**) EDC, HOBt, CH_2_Cl_2_, 24 h, 0 °C; (**vii**) TBAF, THF, 0.5 h, 0 °C.

The 4,4′-dihydroxybenzophenone **6** (protected as the OTBDMS ether **7**) was reacted with the appropriate cyclic ketone (cyclopentanone, cyclohexanone, cycloheptanone, cyclooctanone and 4-methylcyclohexanone) under the McMurry reaction conditions to give the compounds **8a**–**8e** respectively, (Scheme 5).

Compounds **8a**–**8e** then underwent an ethylbromination reaction to afford **9a**–**9e**. Following an amination reaction step, analogues **10a**–**10e** were used in the formation of novel conjugates. Compounds **10a**–**10e** were subsequently deprotected to afford the endoxifen-type cyclofenil analogues **11a**–**11e**. These novel ER ligands containing a basic side chain ether similar to that of endoxifen, were subsequently used for the novel linkage to the CA4-type cytotoxic agent. The acrylic acid combretastatin analogue **1l** was directly coupled to the silyl-protected cyclofenil-based analogues **10a**–**10e** to afford the protected conjugates **12a**–**12e**. This procedure employed EDC as the coupling reagent. The synthesis of this series of silyl-protected conjugates was similar to that for the endoxifen conjugates synthesis **3a**–**3k** the reagent ratio was optimised: 1.2 eq. of acrylic acid, 1.4 eq. DCC, 1.4 eq. HOBt and 1 eq of amine were reacted and the reaction was monitored via TLC. The silyl-protected conjugates **12a**–**12e** were isolated and fully characterised and then treated with TBAF to afford the direct amide conjugates **13a**–**13e** in high yields (Scheme 5).

With all of the conjugate prototypes investigated to date, the synthesis of the conjugates required a coupling reaction between a carboxylic acid group with an amine forming an amide linkage. Coupling of the phenolic functionality of CA-4 with the free carboxylic acid group of diacid linker compounds, forming ester linkages was also investigated. A diacid type linker was chosen to allow for the formation of ester and/or amide bonds with any available phenol and/or amine groups present on the conjugate component-fragments. Therefore, it is envisioned that these diacid fragments can be metabolised easily in vivo thus releasing the conjugate component ligands and possibly exerting a dual action effect.

**Scheme 6:**
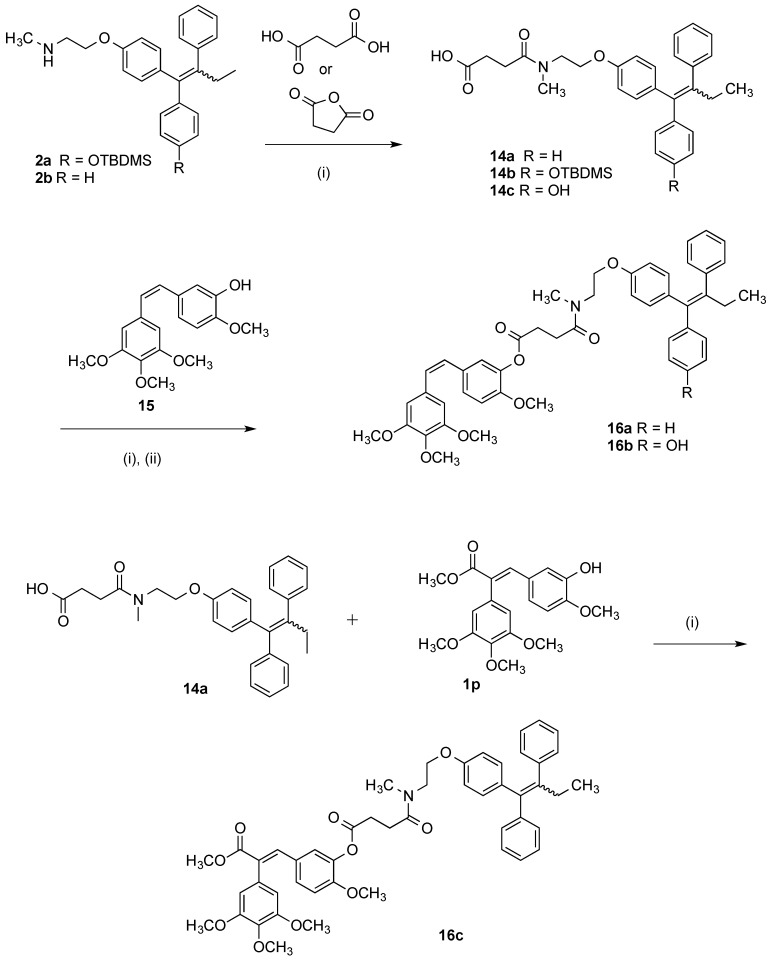
Synthesis of endoxifen-combretastatin conjugates **16a**–**16c**. *Reagents and Conditions*: (**i**) DCC, DMAP, DCM; (**ii**) (a) DCC, DMAP, DCM (b) TBAF, THF.

Desmethyltamoxifen **2b** was initially used as the prototype ER-ligand for the DCC coupling reaction with the dicarboxylic acid (Scheme 6). Succinic acid, DCC and HOBt were reacted with **2b** and an isomeric mixture (*E:Z* = 1:2.3) of the product **14a** was afforded (90% yield) (see Scheme 6). It was found that the reactions involving the formation of the diacid linker compounds from secondary amine, triarylethylenes with acid anhydrides were successful without the need for any additional reagents such as DCC, HOBt or DMAP. Succinic anhydride was reacted successfully with desmethyltamoxifen **2b** to afford the diacid-linker compounds **14a** in 97% yield.

In addition to the preliminary diacid reactions carried out on desmethyltamoxifen **2b,** succinic anhydride was next reacted with the silyl-protected endoxifen analogue **2a** to afford **14b**. The phenol group present on the endoxifen ligand plays an important role in ER-binding and is a desirable functionality on the proposed conjugate structures for improved ER-binding and overall bioactivity. Initially, it was decided to carry out the conjugate prototype reactions using a single linker-ligand **14b**. The succinic linker was chosen as it is a flexible fragment and allows the conjugate some flexibility. Therefore, the prototype succinic-conjugates would not be overly restricted and adopt a configuration for optimal binding.

The use of DCC with HOBt was effective for the majority of the coupling reactions above; however coupling of CA-4 **15** with the diacid linker compound **14a** to afford the conjugate **16a** gave very low yields (Scheme 5). It was then decided to first react the diacid linker component succinic acid with the CA4 structure and then to react the resulting CA4-linker product with the desmethyltamoxifen analogue **2b** forming the amide linkage of the conjugate **16a.** However, initial attempts to couple the succinic acid/anhydride linker directly to CA4 were unsuccessful using a variety of reaction conditions (e.g., DCC/HOBt, Et_3_N, or Mitsunobu conditions, PPh_3_/DIAD). Pettit et al. reported the synthesis of succinic acid esters of combretastatin analogues as CA4 prodrugs and water soluble derivatives [41]. In the present study CA4 **15** was treated with **14b** using DCC coupling conditions with DMAP as a base. The coupling reaction was successful (Scheme 5) and the intermediate protected conjugate was treated with TBAF to give the target compound **16b** as an isomeric mixture (*E:Z* = 1:1) in a high yield (88%). Similarly, **16a** was obtained in high yield (83%) by reaction of **15** with the desmethyltamoxifen **2b**. The related conjugate **16c** was prepared by coupling of the acrylic acid **14a** with the phenolic ester **1s**, to afford a product with additional ester functionality on the acrylic alkene (Scheme 6).

The stability of the target conjugate compounds **16a**, **16b** and **16c**, was evaluated in phosphate buffer at pH values in the range 4–9 also in plasma and the half-life was determined to be greater than 20 h for each compound at these pH values. For the most potent conjugate compound **16b**, >90% remained over pH range 4–9 at 12 h with half life >48 h; and 77% remained intact in plasma at 12 h, with a half life >48 h. We observed no significant degradation of the conjugate. This result indicates that the combretastatin stilbene moiety of the endoxifen conjugate structure is required for optimum interaction with helix 12 of the ER LBD, and contributes to the observed ER antagonistic effects.

### 2.2. Antiproliferative Activity in MCF-7 Breast Cancer Cells 

The antiproliferative activity of the conjugate compounds synthesised was first evaluated using the ER-expressing (ER-dependent) MCF-7 human breast cancer cell line. Alamar Blue dye was used to quantify the cell viability. Cytotoxicity in MCF-7 cells was determined using the LDH assay [60]. Tamoxifen (IC_50_ 2.13 μM), endoxifen (IC_50_ 0.029 μM), and CA-4 (IC_50_ 3.57 nM) were used as postive control compounds in the MCF-7 human breast cancer cell line. The IC_50_ values obtained are in agreement with the previously reported antiproliferative activity for these compounds which are: for CA-4, IC_50_ = 0.008 μM [61,62,63]; tamoxifen IC_50_ = 4.4 μM [64], endoxifen IC_50_ = 50 nM [65] in the MCF-7 human breast cancer cell line.

The conjugate compounds synthesised incorporating the ER-binding triphenylethylene moiety of the SERM endoxifen **3a**–**3k** were initially evaluated for their ability to reduce the viability of the MCF-7 cell line. In the present study, the terminal methylamine basic substituent was designed to mimic the endoxifen basic side chain structure. It was hypothesised that by incorporating a basic side chain linker of the endoxifen structure onto the combretastatin acrylic acid moiety that high affinity ER ligands with potential antagonist activity may be achieved. A range of novel substituted acrylic acids were evaluated in the MCF-7 cell line. The biochemical data observed is presented in Table 2. The most active of the series of conjugate compounds **3i** and **3k** demonstrated micromolar IC_50_ activity (Table 3) with IC_50_ value of 4.2 μM and 1.47 μM respectively. The lead compound **3i** which incorporated the 3,4,5-trimethoxyphenyl Ring A and 3-hydroxy4-methoxyphenyl Ring B of CA4 in the structure of the acrylic acid moiety remains the key compound from this series. We had previously demonstrated the activity of the reverse CA4 conjugate [31]. The diversly substituted acrylic acid analogues were further investigated to enhance the activity seen for **3i**. Compound **3j** containing the 3,5-dimethoxy-4-hydroxy substitution pattern (Ring B of the acrylic acid) displayed weak antiproliferative activity (IC_50_ = 11.8 μM), as did the furan containing compound **3m** (IC_50_ = 36.5 μM).

However, the majority of compounds **3a**–**3m** investigated did not exhibit any antiproliferative activity below IC_50_ of 50 μM. Therefore, this indicates that the substituent on the acrylic acid analogues play a key role in determining the effects of these conjugates on cell proliferation. Compounds **3a**–**3m** all have cLogP values greater than 5 and are predicted to have poor oral absorption. The amides **3n**–**3q** demonstrated low potency when evaluated against the MCF-7 cell line. Compounds **5a**–**5d** were next investigated to determine the effect of the core structure of the conjugate scaffold on the antiproliferative activity. It was decided to determine if both aryl rings (A and B) of the combretastatin structure were required for optimum antiproliferative activity containing cinnamic acids and 3-phenylpropanoic acids which were subsequently coupled to form the desired conjugates **5a**–**5d**. The biochemical data for compounds **5a**–**5d** is presented in Table 3. The four compounds evaluated for this investigation showed reduced activity compared with **3i** and **3k**. Compounds **5a** and **5b** which have the *p*-methoxy and 3-OH substituent (Ring B CA4) showed weak activity, with IC_50_ values of 51.3 and 46.7 μM respectively. Compounds **5c** and **5d** which contain the 3,4,5-trimethoxy substituent (Ring A) did not show any antiproliferative activity below 100 μM. Therefore from the biochemical data for this series it is apparent that the two aryl rings, A and B, of the combretastatin structure with the CA4 type substituents were essential for optimum antiproliferative activity of the conjugate compounds.

The cyclofenil-based analogues **11a**–**11e** were synthesised based on the reported cyclofenil structure which demonstrated impressive ERα and ERβ binding affinities. It was hypothesised that by incorporating a basic side chain onto the cyclofenil structures that high affinity ER ligands with potential antagonist activity may be achieved. The cyclofenil-based analogues were designed to be used as the potential ER targeting ligands for this series of conjugates. As these cyclofenil-based analogues were novel compounds it was decided that they should be first evaluated individually for antiproliferative activity. This data would be beneficial as it would allow the cyclofenil conjugates **13a**–**13e** to be compared directly to ER-ligands **11a**–**11e** for reduced/enhanced activity.

The biochemical data for compounds **11a**–**11e** is presented in Table 3. The cyclofenil-based analogues **11a**–**11e** all showed low micromolar antiproliferative activity in MCF-7 cells with IC_50_ values ranging from 1.38 to 4.78 μM. This activity allowed for the rationalization of the next series of conjugates that incorporated the cyclofenil-based analogues. In this series only **11a** had a CLogP less than 5, and this compound would be expected to demonstrate reasonable oral absorption properties. The conjugates **13a**–**13e** incorporated the novel ER-ligands **11a**–**11e** and were obtained via a direct amide linkage to the acrylic acid analogue **1l**. The biochemical data for conjugates **13a**–**13e** is presented in Table 3. This series of conjugates **13a**–**13e** showed mostly low micromolar activity (IC_50_ range of 1.38–2.5 μM) with two compounds exhibiting sub-micromolar activity (IC_50_ values of 0.182 μM for **13e** and 0.346 μM for **13d**). The activity of the conjugates varies depending on the nature of the cycloalkane ring attached and the most active conjugates, **13d** and **13e** contains the cyclooctane and cyclohexane rings respectively. The compounds **13a**–**13e** are predicted to have poor oral absorption with CLogP greater than 5.

Of the ester linked conjugates, the combretastatin containing compounds **16a** and **16b** were the most potent conjugates in this series with antiproliferative activity in MCF-7 cell line of IC_50_ = 90 nM and 5.7 nM respectively. The cytotxicity of compounds **16a** and **16b** was determined in the lactate dehydrogenase (LDH) assay to be 13.2% and 4.1% respectively, and compares favourably with the cytoxicity of endoxifen (23%) and CA-4 (13%) when evaluated in the same assay.

### 2.3. NCI 60 Cell Line 

The activity of conjugate compound **16a** was evaluated using a 60-cell line screen facility of different cancer cell lines of diverse tumour origin in the National Cancer Institute (NCI, Bethesda, MD, USA) Division of Cancer Treatment and Diagnosis (DCTD)/Developmental Therapeutics Programme (DTP). In the one-dose screen, compound **16a** displayed very high growth inhibition in the cell lines of colon cancer HCC-2998 (95%), HCT-116 (98%), HCT-15 (99%) and HT29 (93%); breast cancer BT-549 (94%), MCF-7 (90%) and MDA-MB-468 (99%); melanoma M14 (99%); CNS cancer SF-295 (94%) and U251 (90%) when evaluated at 10 μM concentration. The compound caused between 80–89% growth inhibition in a further 10 cell lines. The compound **16a** displayed GI_50_ (IC_50_) values within the range 10–72 nM for most of the 60 cancer cell lines. Compound **16a** displayed a GI_50_ (IC_50_) value of 36 nM and a LC_50_ value greater than 100 μM in the MCF-7 breast cancer cell line, (Table 4), indicating a significant therapeutic window between the concentration required for inhibition of cancer cell growth, and the concentration that is determined to be toxic to MCF-7 breast cancer cells.

As **16a** does not display selectivity and enhanced activity towards the ER-positive cell line, it suggests that **16a** may be exerting its potent activity by other biological mechanisms (i.e., inhibition of tubulin polymerisation) other than through the ER alone. Again, this is a promising result and highlights the possible therapeutic applications for the prototype ER-conjugates. Future research is being undertaken to determine what other mechnisms this compounds may inhibit in cancer cells. For **16a** the COMPARE analysis was run on a database of common anti-cancer agents (JobID: 37888) and the larger more comprehensive database including natural products and other submitted agents (JobID: 37889). The highest correlation coefficient achieved was 0.629 in relation to the synthetic nucleoside antitumour, DNA and RNA synthesis inhibitor tiazofurin [66]. CA-4 **15** was listed as a high-ranking hit with a correlation coefficient of 0.658. A correlation coefficient of above 0.6 is considered a positive correlation. This result supports the suggestion that the combretastatin moiety plays an important role in the activity profile of this ester-conjugate as it has a similar pattern or mechanism of action.

### 2.4. Estrogen Receptor Binding Studies

It was necessary to test the binding efficiency of each compound for both ER isoforms (ERα and ERβ), to determine how effectively the ligand binds to the receptor and whether the compound is acting as an antagonist or agonist. The competitive binding experiments were conducted using a fluorescence polarization assay which consists of purified baculovirus expressed human ERα and ERβ and fluoromone, a fluorescein labelled estrogen ligand [67,68]. The most active SERM type compounds and conjugates incorporating an ER type ligand from the antiproliferative assay were selected for ER binding study. The selectivity value reported is the ratio of relative binding affinity (RBA) values ERα relative to ERβ, for each of the compounds. For selectivity values greater than 1, the compounds have a more pronounced affinity for ERα binding site, while for values less than 1, the compounds have a more pronounced affinity for the ERβ binding site.

The most potent compounds evaluated for both ERα and ERβ affinity exhibit impressive binding activity. Compound **3i** displayed ER binding activity in the nanomolar region for both ER isoforms (IC_50_ ERα 182 nM and IC_50_ ERβ 436 nM), with selectivity (3.13) for ERα thus demonstrating that the incorporation of a combretastatin-endoxifen hybrid scaffold structure results in a compound with potential SERM properties. The cyclofenil type compounds **11c** and **11e** display nanomolar activity in ERβ (IC_50_ = 199 nM and IC_50_ = 67 nM respectively) and low micromolar activity in ERα (IC_50_ = 1.738 μM and IC_50_ = 3.162 μM respectively), with selectivity for ERβ in both cases. Compound **11e** which was a cyclofenil type ligand, exhibited the greatest selectivity (46 fold) towards the ERβ isoform while compound **13e** which was a direct conjugate of **11e** displayed the greatest selectivity (12.3 fold) towards ERα. The corresponding cyclofenil conjugate **13e** demonstrated potent nanomolar binding activity for ERα (IC_50_ = 19.0 nM) and ERβ (IC_50_ = 229 nM).

The CA4 conjugate **16b** displays significant ER binding affinities which help explain the antiproliferative activity of the conjugate. Interestingly the succinic-endoxifen linker compound **14c** displays potent binding affinities in both ERα and ERβ with competitive binding IC_50_ values of 11.3 nM (ERα) and 6.7 nM (ERβ). These values represent an approximately 3-fold increase in binding affinity in both ER isoforms when compared to 4-hydroxytamoxifen. The carboxylic acid group may interact favourably with a residue in the binding site. Interestingly, the carboxylic acids such as **14a** (Table 3) closely related (differing only in the hydroxy moiety on the triarylethylene aryl ring scaffold) displayed no significant antiproliferative activity.

As in the antiproliferative assays, the conjugate **16b** demonstrated impressive ER binding results and is suitable as a possible lead compound for further development. The conjugate displays nanomolar binding to the ERα and ERβ, [IC_50_ ERα 52.1 nM and IC_50_ ERβ 115 nM]. The phenolic hydroxy functionality present on the endoxifen fragment can interact favourably with the residues Glu353, Arg394 and His524 in the ER binding site through hydrogen bonding which can explain the high binding affinity. The combretastatin fragment may also form favourable interactions in the binding site as there is comparable ERα binding affinity with endoxifen. Presumably, the hydroxy functionality present on the combretastatin fragment is involved in hydrogen bonding.

Often the relative binding affinity (RBA) of estrogen receptor ligands is reported. Estradiol is typically used as the reference ligand and is taken as the 100% binding value. Using the reference IC_50_ values obtained from the literature for estradiol in the ERα (5.7 nM) and ERβ (5.6 nM), the RBAs of the selected conjugates were calculated (see Table 5). All of the conjugates investigated in the ER competitive binding assays had RBA values greater than or equal to 1%. The RBA values between of 5–16% would be considered moderate binding while the RBA value for **14c** demonstrates strong RBA ERα = 50.44, ERβ = 83.6. The lead conjugates e.g., **13e** and **16b** display very impressive RBA values of ERα = 30 and ERβ 2.44 for **13e** and ERα = 10.94, ERβ 4.87 for **16b**.

### 2.5. Effects of Selected Active Compounds on Cell Cycle and Cell Death.

**Figure 2:**
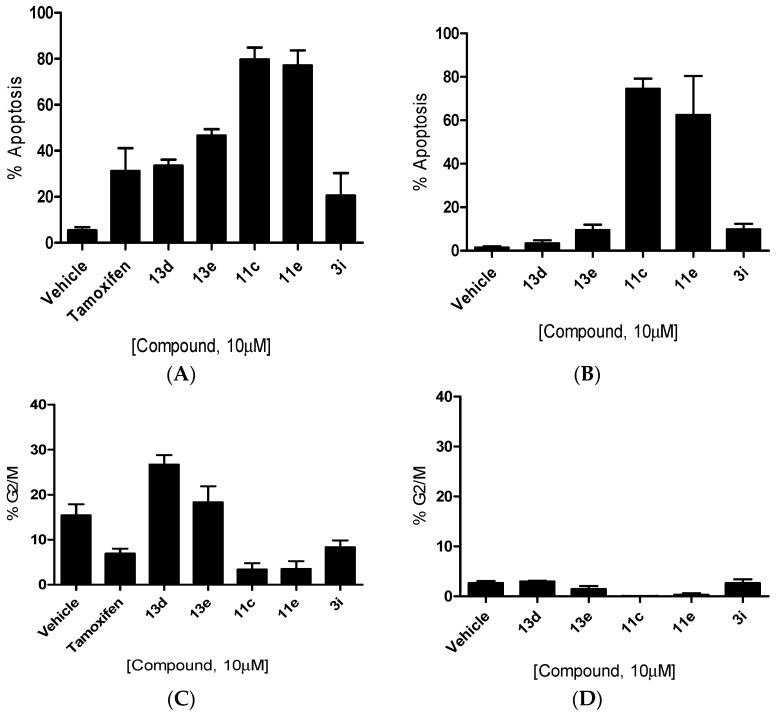
Compounds **3i**, **11c**, **11e**, **13d** and **13e** potently induce apoptosis in MCF-7 cells, but not healthy PBMCs. Cells were treated for 72 h with a vehicle or the indicated compounds. After the required incubation period, cells were fixed in 1 mL ethanol and 100 μL PBS and stained with propidium iodide. Cells were subsequently analysed by flow cytometry to determine the percentage of apoptotic (**A**) MCF-7 cells and (**B**) PBMCs and G2/M-arrested (**C**) MCF-7 cells and (**D**) PBMCs. Values represent the mean ± SEM of three independent experiments.

Based on the cell viability assay results, a selected cohort of the most active compounds from each series (IC_50_ values 0.182–4.57 µM) were subjected to cell cycle analysis, Figure 2. Flow cytometry with propidium iodide stained cells was carried out to analyse the percentage of cells in each stage of the cell cycle and the number of apoptotic or dead cells. Samples were treated for 72 h at 10 µM. Results showed that all compounds tested increased apoptosis when compared to vehicle-treated cells. Of the active compounds, the cyclofenil based analogues **11c** and **11e** proved significantly more active than the positive control tamoxifen (*p* value < 0.001). The most potent compound tested, **11c** induced 79.7 ± 5.2% apoptosis at 10 µM while compound **11e** induced 76% apoptosis. Conjugates **13c**, **13d** and **13e** demonstrated pro-apoptotic effects of 20%, 36% and 48% respectively.

Interestingly, in addition to inducing apoptosis, the CA4 direct amide cyclofenil based conjugate **13d** (*p* value < 0.01) also induced a significant G2/M-arrest (27%) in MCF-7 cells when compared to vehicle-treated cells, with **13e** causing a lesser effect on G2M arrest of 18%. A G2/M arrest typically, though not necessarily always, precedes apoptosis. Future studies at earlier time-points (24 h and 48 h) will be undertaken to establish if the other active compounds in this cohort also induce a G2/M arrest preceding apoptosis. A G2/M arrest is commonly observed with tubulin-targeting compounds and so this data suggests that the tubulin-targeting ability of combretastatins is maintained when complexed to various ligands with induction of significant apoptosis.

Next, the same cohort of compounds was tested on peripheral blood mononuclear cells (PBMCs) isolated from the blood of healthy donors. Cells were treated with 10 µM of each compound for 72 h and subjected to flow cytometry. Results showed that only two of the compounds, (**11c** and **11e**), the most active compounds in the MCF-7 cells), induced apoptosis in PBMCs, with no toxic effects observed with the other compounds. None of the compounds caused a G2/M arrest in PBMCs. The potent activity of **13d**, **13e** and **3i** of this cohort of compounds in MCF-7 breast cancer cells and their lack of toxicity in normal PBMCs make them ideal candidates for further anti-cancer studies.

### 2.6. Molecular Modelling of ***11e***, ***16b*** and ***13e*** in ERα and ERβ

A retrospective molecular modelling study of **11e**, **16b** and **13e** was undertaken to rationalise the selectivity profile differences of the compounds for ERα and ERβ. Two amino acid differences within the ligand binding pocket of the ER isoforms are replacement of Met412 and Leu384 in ERα with Ile373 and Met336 respectively in ERβ which have an impact on the following results.

The 3ERT X-ray structure of hERα co-crystallised with 4-hydroxytamoxifen (4-OHT) [69] was downloaded from the PDB website. For ERβ the 1NDE X-ray structure co-crystallised with a triazine modulator was used [70]. An in-depth binding poses analysis on compounds **11e**, **16b** and **13e** was performed. The top five docked poses of each compound in each isoform were analysed and mapping to the X-ray structure ligands binding poses was considered. Amino acid numbering corresponds to AA(#ERα)/(#ERβ) unless specified in the text.

**Figure 3:**
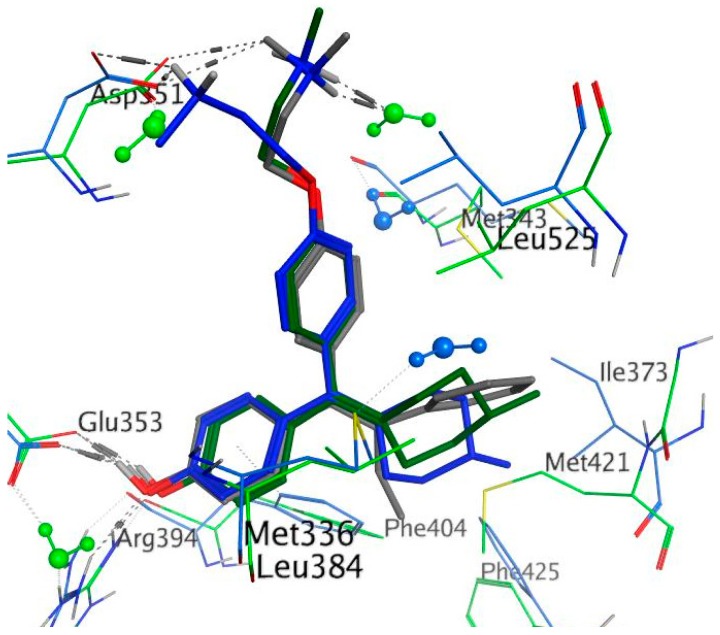
Ranked poses of **11e** in ERα and ERβ overlaid on 4-OHT X-ray structure. Carbon atoms of **11e** are illustrated in dark green in ERα, dark blue in ERβ and grey in 4-OHT (oxygen atoms are red; nitrogen in dark blue; sulphur in yellow). ERα and associated water molecules are in light green, ERβ and associated water molecules are in light blue. Amino acid numbering is for ERα except when different in ERβ where both labels are used. PDB structures 3ERT [69] and 1NDE [70] were used for molecular docking.

**11e** docked well into both ER isoforms and recapitulated the interactions formed by 4-OHT in the 3ERT X-ray structure. The 4-methylcyclohexylidene moiety occupies a hydrophobic pocket delineated by Leu525, Met343, Met421 (Ile373 in ERβ) and Leu384 (Met336 in ERβ). In ERα each of these amino acids are closer to the ligand and form a tighter binding sub-pocket than for ERβ. In 4-OHT the D-ring phenyl group occupies this sub-pocket and is a planar conformationally rigid structure but the corresponding moiety in **11e** is 4-methylcyclohexylidene which is bulkier and converts between multiple conformational states. This results in clashing with the more tightly packed ERα amino acids. Additionally, in ERβ Leu384 is mutated to Met336 which, in the 1NDE X-ray structure, forms a hydrogen bond acceptor interaction (HBA) with a cocrystallised water molecule, thereby decreasing the hydrophobicity of the sub-pocket. Both of these factors result in a 47-fold binding affinity preference for ERβ over ERα of this compound. The similarities in overlays between **11e** docked in ERα and ERβ is illustrated in Figure 3.

**Figure 4:**
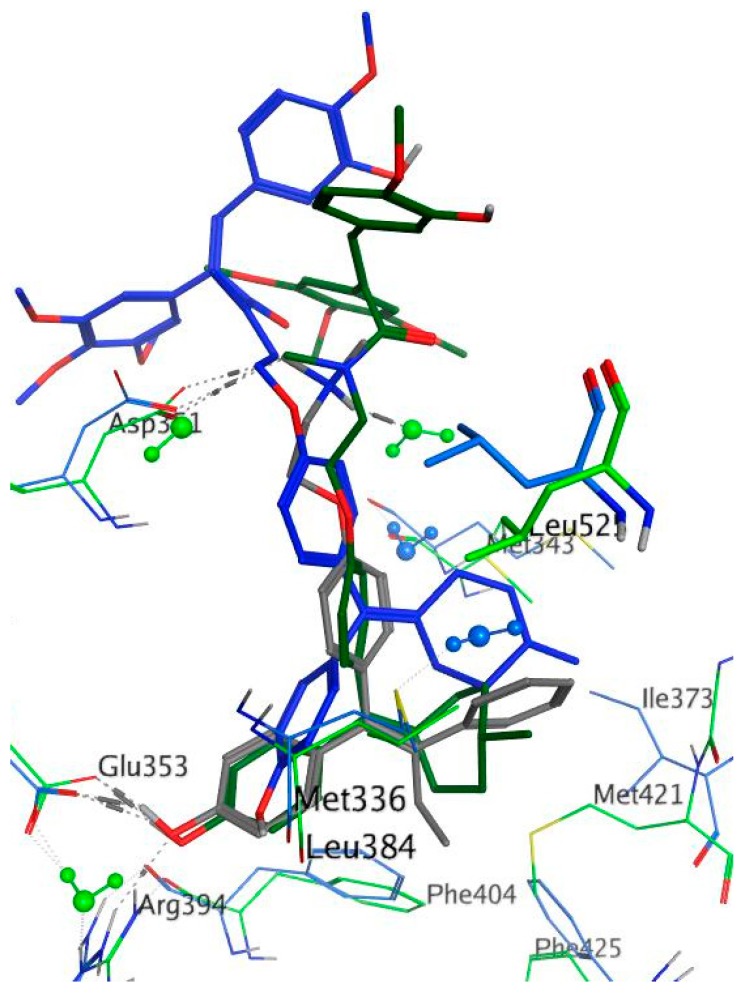
Ranked poses of **13e** in ERα and ERβ overlaid on 4-OHT X-ray structure. The potential clashing interaction between Leu525/476 (rendered in tube style) of ERβ with the methylamide of **13e** is present. Carbon atoms of **13e** are illustrated in dark green in ERα, dark blue in ERβ and grey in 4-OHT (oxygen atoms are red; nitrogen in dark blue; sulphur in yellow). ERα and associated water molecules are in light green, ERβ and associated water molecules are in light blue. Amino acid numbering is for ERα except when different in ERβ where both labels are used. PDB structures 3ERT [69] and 1NDE [70] were used for molecular docking.

Docking of compound **13e** in ERα retained the same 4-OHT mapping binding mode as observed for **11e**. Analogous to the situation with **16b**, the methylamide moiety of **13e** would clash with Leu476 of ERβ so the ligand is significantly shifted upwards thereby unable to make optimal interactions within the binding pocket. This is reflected in the 12-fold selectivity of the ligand for ERα over ERβ and is shown in Figure 4.

**16b** docked with a perfect overlay on the ERα OHT X-ray. The corresponding docked pose in ERβ is capable of making the same hydrogen bond donor (HBD) interaction as the 4-OHT A-ring phenolic oxygen atom but the ligand is shifted upwards in the binding site. A contributing factor to this is the repositioning of Leu525/476 which is adjacent to the 4-OHT C-ring in ERα but flipped 180° upwards towards the binding pocket entrance in ERβ.

**Figure 5:**
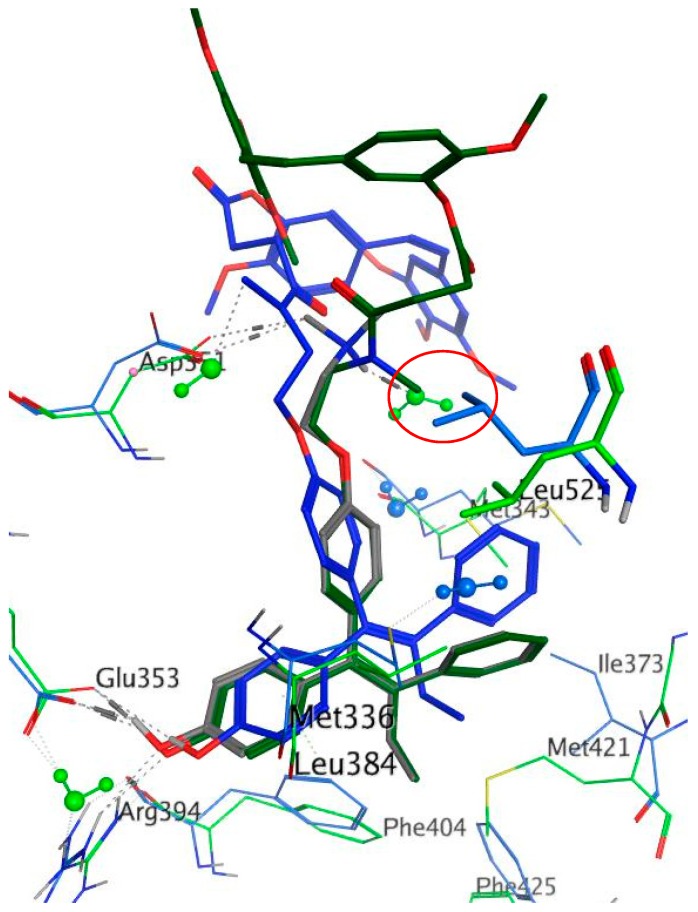
Ranked poses of **16b** in ERα and ERβ overlaid on 4-OHT X-ray structure. The potential clashing interaction between Leu525/476 (rendered in tube style) of ERβ with the methylamide of **16b** is circled in red. Carbon atoms of **16b** are illustrated in dark green in ERα, dark blue in ERβ and grey in 4-OHT (oxygen atoms are red; nitrogen in dark blue; sulphur in yellow). ERα and associated water molecules are in light green, ERβ and associated water molecules are in light blue. Amino acid numbering is for ERα except when different in ERβ where both labels are used. PDB structures 3ERT [69] and 1NDE [70] were used for molecular docking.

As highlighted with a red circle in Figure 5, this repositioned Leu now clashes with the methylamide of the side-chain of **16b** in ERβ so the ligand is shifted away and twisted so as to avoid electrostatic repulsion between the amide carbonyl oxygen atom and Asp351/303. This is revealed in a 7-fold binding affinity preference for ERα over ERβ.

## 3. Experimental Section

### 3.1. General Information

All reagents were commercially available and were used without further purification unless otherwise indicated [19]. Tetrahydrofuran (THF) was distilled immediately prior to use from Na/Benzophenone under a slight positive pressure of nitrogen, toluene was dried by distillation from sodium and stored on activated molecular sieves (4 Å) and dichloromethane was dried by distillation from calcium hydride prior to use. Uncorrected melting points were measured on a Gallenkamp apparatus. Infra-red (IR) spectra were recorded as thin film on NaCl plates, or as potassium bromide discs on a Perkin Elmer FT-IR Specrtum 100 spectrometer (Perkin Elmer, Waltham, MA, USA). ^1^H- and ^13^C-nuclear magnetic resonance (NMR) spectra were recorded at 27 °C on a Bruker Avance DPX 400 spectrometer (400.13 MHz, ^1^H; 100.61 MHz, ^13^C) (Bruker, Billerica, MA, USA) at 20 °C in CDCl_3_ (internal standard tetramethylsilane (TMS)), CD_3_OD or DMSO-*d*_6_ by Dr. John O’Brien and Dr. Manuel Ruether in the School of Chemistry, Trinity College Dublin. For CDCl_3_, ^1^H-NMR spectra were assigned relative to the TMS peak at 0.00 δ and ^13^C-NMR spectra were assigned relative to the middle CDCl_3_ triplet at 77.00 ppm. For CD_3_OD, ^1^H and ^13^C-NMR spectra were assigned relative to the centre peaks of the CD_3_OD multiplets at 3.30 δ and 49.00 ppm respectively. Electrospray ionisation mass spectrometry (ESI-MS) was performed in the positive ion mode on a liquid chromatography time-of-flight (TOF) mass spectrometer (Micromass LCT, Waters Ltd., Manchester, UK), equipped with electrospray ionization (ES) interface operated in the positive ion mode at the High Resolution Mass Spectrometry Laboratory by Dr. Martin Feeney in the School of Chemistry, Trinity College. Mass measurement accuracies of < ±5 ppm were obtained. Low resolution mass spectra (LRMS) were acquired on a Hewlett-Packard 5973 MSD GC-MS system (Hewlett-Packard, Palo Alto, CA, USA) in electron impact (EI) mode. R_f_ values are quoted for thin layer chromatography on silica gel F-254 plates (Merck, Kenilworth, NJ, USA) unless otherwise stated. Compounds were visually detected with UV at 254 and 366 nm. Flash column chromatography was carried out on Merck Kieselgel 60 (particle size 0.040–0.063 mm), aluminium oxide, (activated, neutral, Brockmann I, 50 mesh, Aldrich, Darmstadt, Germany) or Aldrich aluminium oxide, (activated, acidic, Brockmann I, 50 mesh). All products isolated were homogenous on TLC. Analytical high-performance liquid chromatography (HPLC) to determine the purity of the final compounds was performed using a Waters 2487 Dual Wavelength Absorbance detector, a Waters 1525 binary HPLC pump, a Waters In-Line Degasser AF and a Waters 717 plus Autosampler (Waters Corporation, Milford, MA 01757, USA). The column used was a Varian Pursuit XRs C18 reverse phase 150 × 4.6 mm chromatography column (Agilent, Santa Clara, CA, USA). Samples were detected using a wavelength of 254 nm. All samples were analyzed using acetonitrile (70%): water (30%) over 10 min and a flow rate of 1 mL/min. Unless otherwise indicated, the purity of the final products was ≥95%. Chromatograpic separations were also carried out on Biotage SP4 instrument (Biotage AB, Uppsala, Sweden). Microwave experiments were carried out with the Biotage Initiator and Discover CEM microwave synthesisers. Combretastatin A-4 (CA4) **15** [50,52] and the acrylic acids **1a** [71], **1b** [72], **1d** [73], **1e** [73], **1g** [74], **1i** [75], **1l** [76], **1m** [77], **1n** [50], **1s** [50] were prepared as previously reported.

### 3.2. Chemistry 

#### 3.2.1. General Method for Synthesis of Acrylic Acids **1a**–**1r**

*Method A*: A mixture of the appropriate the appropriate phenylacetic acid (0.50 g, 1 equivalent), benzaldehyde (1 equivalent), acetic anhydride (2 mL) and triethylamine (1 mL) were heated under reflux for 3–5 h. After acidification with concentrated hydrochloric acid (5 mL), the resulting solid was filtered off and recrystallised to yield the required acrylic acid. *Method B*: A mixture of the appropriate benzaldehyde (1 eq.), the appropriate phenylacetic acid (700 mg, 1 eq.), acetic anhydride (2 mL) and triethylamine (1 mL) were reacted in the microwave reactor at a 120 °C and for 30 min. After acidification with concentrated hydrochloric acid (5 mL), the resulting solid was filtered and recrystallised to afford the required acrylic acid.

*(E)-2,3-bis(3,4,5-Trimethoxyphenyl)acrylic acid* (**1c**). 3,4,5-Trimethoxybenzaldehyde (700 mg, 3.59 mmol) and 3,4,5-trimethoxyphenylacetic acid (744 mg, 3.59 mmol) were reacted following the general method A. Recrystallisation from methanol afforded the acrylic acid as fine yellow solid (484 mg, 33%). 3,4,5-Trimethoxybenzaldehyde (606 mg, 3.09 mmol) and 3,4,5-trimethoxyphenylacetic acid (700 mg, 3.09 mmol) were reacted following the general method B. Recrystallisation from methanol afforded the acrylic acid as fine yellow solid (1.2 g, 96%), m.p. 169–172 °C. ^1^H-NMR (CDCl_3_): δ 7.85 (s, 1H, C=CH), 6.53 (s, 2H, Ar-H), 6.42 (s, 2H, Ar-H), 3.88 (s, 3H, OCH_3_), 3.85 (s, 3H, OCH_3_), 3.83 (s, 6H, 2 × OCH_3_), 3.63 (s, 6H, 2 × OCH_3_). ^13^C-NMR (DMSO-*d*_6_): δ 171.4, 160.9, 153.1, 152.2, 141.7, 139.0, 137.1, 129.8, 128.7, 107.9, 106.9, 99.9, 60.5 (OCH_3_), 60.4 (OCH_3_), 55.2 (OCH_3_), 55.0 (OCH_3_). IR: ν_max_ (KBr) cm^−1^: 3440.90, 2938.31, 1668.56, 1580.98, 1505.11, 1413.46, 1272.44, 1240.40. HRMS (EI): Found 405.1452 [M + H]^+^, C_21_H_25_O_8_ requires 405.1471. 405.1549.

*(E)-3-(3-Hydroxyphenyl)-2-(3,4,5-trimethoxyphenyl)acrylic acid* (**1f**). 3-Hydroxybenzaldehyde (377 mg, 3.09 mmol), 3,4,5-trimethoxyphenylacetic acid (700 mg, 3.09 mmol) were reacted following the general method B. Recrystallisation from methanol afforded the acrylic acid as fine yellow soild (711 mg, 60%), m.p. 230–231 °C. ^1^H-NMR (DMSO-*d*_6_): δ 7.61 (s, 1H, C=CH), 7.05 (t, 1H, 8 Hz, Ar-H), 6.67 (dd, 1H, 8 Hz, 1 Hz, Ar-H), 6.54–6.52 (m, 2H, Ar-H), 6.44 (s, 2H, Ar-H), 3.70 (s, 3H, O CH_3_), 3.67 (s, 6H, OCH_3_). ^13^C-NMR (DMSO-*d*_6_): δ 168.40 (COOH), 157.02, 152.93, 139.05, 136.96, 135.59, 132.96, 131.72, 129.19, 121.28, 116.85, 116.27, 106.74, 60.12 (OCH_3_), 55.91 (OCH_3_). IR: ν_max_ (KBr) cm^−1^: 3362.7, 2941.0, 2836.0, 2627.4, 1681.6, 1584.8, 1411.8, 1239.2, 1125.0, 997.3, 965.6, 688.2. HRMS (EI): Found 353.1003 [M + Na]^+^, C_18_H_18_O_6_Na requires 353.1001. Hydrolysis of (*E*)-3-(3-acetoxyphenyl)-2-(3,4,5-trimethoxy-phenyl)acrylic acid **1k** with NAOH/MeOH for 12 h followed by acidification to pH 4 afforded **1f** in 88% yield.

*(E)-3-(2,3,4-Trimethoxyphenyl)-2-(3,4,5-trimethoxyphenyl)acrylic acid* (**1h**). 2,3,4-Trimethoxy-benzaldehyde (607 mg, 3.09 mmol) and 3,4,5-trimethoxyphenylacetic acid (700 mg, 3.09 mmol) were reacted following the general method B. Recrystallisation from methanol afforded the acrylic acid as fine yellow solid (374 mg, 30%), m.p. 222–225 °C. ^1^H-NMR (DMSO-*d*_6_): δ 7.91 (s, 1H, C=CH), 6.61 (d, 1H, 9 Hz, Ar-H), 6.46 (m, 3H, Ar-H), 3.86 (s, 3H, OCH_3_), 3.74 (s, 6H, OCH_3_), 3.70 (s, 3H, OCH_3_), 3.68 (s, 6H, OCH_3_). ^13^C-NMR (DMSO-*d*_6_): δ 168.96, 154.79, 153.48, 153.36, 141.88, 137.35, 133.34, 132.05, 124.97, 121.23, 108.20, 107.30, 61.95 (OCH_3_), 60.88 (OCH_3_), 60.58 (OCH_3_), 56.40 (OCH_3_), 56.31 (OCH_3_) IR: ν_max_ (KBr) cm^−1^: 3440.90, 2938.31, 1668.56, 1580.98, 1505.11, 1413.46, 1272.44, 1240.40. HRMS (EI): Found 427.1370 [M + Na]^+^, C_21_H_24_O_8_Na requires 427.1369.

*(E)-3-(4-Hydroxy-3,5-dimethoxyphenyl)-2-(3,4,5-trimethoxyphenyl)acrylic acid* (**1j**). 3,5-Dimethoxy-4-hydroxybenzaldehyde (350 mg, 1.91 mmol) and 3,4,5-trimethoxyphenylacetic acid (433 mg, 1.91 mmol) were reacted following the general method B. Recrystallisation from methanol afforded the acrylic acid as pale yellow solid (400 mg, 48%), m.p. 212–214 °C. ^1^H-NMR (CDCl_3_): δ 7.86 (s, 1H, C=CH), 6.52 (s, 2H, Ar-H), 6.44 (s, 2H, Ar-H), 3.86 (s, 3H, OCH_3_), 3.83 (s, 6H, OCH_3_), 3.59 (s, 6H, OCH_3_). ^13^C-NMR (CDCl_3_): δ 171.94, 168.05, 153.40, 151.32, 141.59, 137.23, 131.51, 130.88, 130.35, 139.41, 107.34, 105.99, 60.34 (OCH_3_), 55.77 (OCH_3_), 55.37 (OCH_3_), 19.97 (CH_3_). IR: ν_max_ (KBr) cm^−1^: 3438.9, 2942.4, 1765.4, 1676.7, 1594.8, 1506.7, 1420.4, 1272.3, 1236.7, 1127.5, 999.8, 906.9. HRMS (EI): Found 455.1321 [M + Na]^+^, C_22_H_24_O_9_Na requires 455.1318.

*(E)-3-(3-Acetoxyphenyl)-2-(3,4,5-trimethoxyphenyl)acrylic acid* (**1k**). 3-Hydroxybenzaldehyde (350 mg, 2.3 mmol) and 3,4,5-trimethoxyphenylacetic acid (520 mg, 2.3 mmol) were reacted following the general method B. Recrystallisation from methanol afforded the acrylic acid as fine yellow solid (515 mg, 56%), m.p. 176–180 °C. ^1^H-NMR (CDCl_3_): δ 7.88 (s, 1H, C=CH), 6.95 (d, 1H, 8Hz, Ar-H), 6.88 (dd, 1H, 18 Hz, 2 Hz, Ar-H), 6.65 (s, 2H, Ar-H), 6.50 (s, 2H, Ar-H), 3.88 (s, 3H, OCH3), 3.81 (s, 6H, O CH3), 2.30 (s, 3H, COCH3). ^13^C-NMR (CDCl_3_): δ 171.72, 153.36, 150.13, 141.07, 140.40, 137.21, 132.37, 130.80, 130.36, 124.46, 122.28, 113.19, 105.96, 60.38 (OCH3), 55.72 (OCH3), 54.88 (OCH3), 20.18 (CH3). IR: νmax (KBr) cm^−1^: 3362.7, 2941.0, 2836.0, 2627.4, 1681.6, 1584.8, 1411.8, 1239.2, 1125.0, 997.3, 965.6, 688.2. HRMS (EI): Found 395.1110 [M + H]^+^, C_20_H_20_O_7_Na requires 395.1107.

*(E)-2-(3,4,5-Trimethoxyphenyl)-3-(5-methylthiophen-2-yl)but-2-enoic acid* (**1p**). 5-Methyl-2-thiophene-carboxaldehyde (389 mg, 0.337 mL, 3.09 mmol) and 3,4,5-trimethoxyphenylacetic acid (700 mg, 2.3 mmol) were reacted following the general method B. Recrystallisation from methanol afforded the acrylic acid as a dark yellow solid (375 mg, 36%), m.p. 212–217 °C. ^1^H-NMR (DMSO-*d*_6_): δ 7.87 (s, 1H, C=CH), 7.24 (d, 1H, 3.6 Hz, thiophene-H), 6.74 (d, 1H, 3.6 Hz, thiophene-H), 6.47 (s, 2H, Ar-H), 3.72 (s, 9H, OCH3), 2.31 (s, 3H, CH3). ^13^C-NMR (DMSO-*d*_6_): δ 15.23 (CH3), 55.93 (OCH3), 60.20 (OCH3), 106.94, 125.42, 128.27, 130.99, 133.25, 134.85, 136.05, 137.44, 153.36, 168.10. IR: νmax (KBr) cm^−1^: 3426.13, 2998.75, 2537.61, 1662.03, 1581.35, 1412.40, 1283.72, 1128.23. HRMS (EI): Found 357.0763 [M + Na]^+^, C_17_H_18_O_5_NaS requires 357.0773.

*(E)-3-(Furan-3-yl)-2-(3,4,5-trimethoxyphenyl)acrylic acid* (**1q**). 3-Furaldehyde (296 mg, 0.26 mL, 3.09 mmol) and 3,4,5-trimethoxyphenylacetic acid (700 mg, 2.3 mmol) were reacted following the general method B. Recrystallisation from methanol afforded the acrylic acid as a dark brown solid (460 mg, 49%), m.p. 188–191 °C. ^1^H-NMR (DMSO-*d*_6_): 7.90 (s, 1H, C=CH), 7.74 (s, 1H, C=CH), 7.29–7.25 (m, 1H, C=CH), 6.86 (m, 1H, C=CH), 6.38 (s, 2H, Ar-H), 3.92 (s, 3H, OCH_3_), 3.81 (s, 6H, OCH_3_). ^13^C-NMR (DMSO-*d*_6_): 168.3, 153.0, 143.7, 143.6, 139.0, 138.4, 138.0, 124.4, 108.2, 105.1, 60.8 (OCH_3_), 56.1 (OCH_3_). IR: ν_max_ (KBr) cm^−1^: 3447.5, 2938.9, 2627.6, 1674.9, 1582.2, 1504.8, 1909.5, 1504.8, 1909.5, 1236.5, 1127.5, 1007.9, 803.2. HRMS (EI): Found 327.0839 [M + Na]^+^, C_16_H_16_O_6_Na requires 327.0845.

*(E)-3-(3-Methoxy-4-hydroxyphenyl)-2-(3,4,5-trimethoxyphenyl)acrylic acid* (**1r**). 4-Hydroxy-3-methoxy-benzaldehyde (470 mg, 3.09 mmol) and 3,4,5-trimethoxyphenylacetic acid (700 mg, 3.09 mmol) were reacted following the general method B. Recrystallisation from methanol afforded the acrylic acid as fine yellow needles (923 mg, 83%), m.p. 237–239 °C. ^1^H-NMR (DMSO-*d*_6_): 12.45 (br s, 1H, COOH), 8.98 (br s, 1H, OH), 7.57 (s, 1H, C=CH), 6.81 (d, *J* = 7 Hz, 1H, Ar-H), 6.60 (d, *J* = 7 Hz, 1H, Ar-H), 6.53 (s, 1H, Ar-H), 6.44 (s, 2H, Ar-H), 3.73 (s, 3H, OCH_3_), 3.71 (s, 3H, OCH_3_), 3.69 (s, 6H, OCH_3_). ^13^C-NMR (DMSO-*d*_6_): 168.61 (COOH), 153.09, 148.89, 145.84, 139.13, 136.90, 132.18, 130.31, 127.02, 122.99, 117.19, 111.49, 106.65, 60.12 (OCH_3_), 55.91 (OCH_3_), 55.45 (OCH_3_). IR: ν_max_ (KBr) cm^−1^: 3423.9 (br), 2939.8, 1671.3, 1585.2, 1509.6, 1455.3, 1411.1, 1268.3, 1239.3, 1126.2. HRMS (EI): Found 383.1103 [M + Na]^+^, C_19_H_20_O_7_Na requires 383.1107.

*(4-((E/Z)-1-(4-(2-Methylaminoethoxy)phenyl)-2-phenylbut-1-enyl)phenoxy)(tert-butyl)dimethylsilane* (**2a**). (4-((*E/Z*)-1-(4-(2-Bromoethoxy)phenyl)-2-phenylbut-1-enyl)phenoxy)(*tert*-butyl) dimethylsilane (1.457 g, 2.69 mmol) was dissolved in a solution (2 M) of methylamine in THF (1.678 g, 26.99 mL, 54 mmol). The reaction mixture was stirred in a sealed pressure tube for 48 h at 60 °C, followed by the addition of dichloromethane (100 mL). The organic phase was washed with a sodium bicarbonate/sodium carbonate (pH 10), (50 mL). The aqueous phase was then extracted with 3 × 50 mL dichloromethane. The organic layers were combined, dried over sodium sulfate and concentrated in vacuo to yield crude product. The material was purified via flash chromatography on silica gel (DCM:EtOAc 3:1) to afford the product as a brown resin. (1.24 g, 94%). ^1^H-NMR (CDCl_3_): δ 7.20–6.50 (m, 26H, Ar-H), 4.11 (t, 0.43 × 4H, *J* = 5.0 Hz, CH_2_), 3.95 (t, 0.43 × 4H, *J* = 5.0 Hz, CH_2_), 3.36 (s, 2H, NH), 2.99 (s, 0.57 × 4H, CH_2_), 2.89 (s, 0.43 × 4H, CH_2_) 2.53 (m, 10H, NCH_3_, CH_2_), 1.01–0.92 (m, 24H, CH_3_, (CH_3_)_3_), 0.26 (s, 0.43 × 12H, Si(CH_3_)_2_), 0.13 (s, 0.57 × 12H, Si(CH_3_)_2_). ^13^C-NMR (CDCl_3_): δ 156.94, 156.10, 153.84, 153.06, 142.19, 142.11, 140.65, 140.59, 137.53, 137.44, 136.35, 136.06, 136.02, 135.68, 131.57, 131.43, 130.20, 130.12, 129.25, 127.41, 127.33, 125.48, 125.44, 119.12, 118.54, 113.54, 112.80, 65.99, 65.73, 50.12, 50.03, 49.47, 35.39, 35.26, 28.60, 28.47, 25.26, 25.24, 17.75, 13.24, 13.21, −4.80, −4.91. IR: ν_max_ (KBr) cm^−1^: 3431.2, 1606.0, 1508.0, 1241.3, 1173.2, 1099.5, 1078.8, 1036.9, 938.9, 846.8. HRMS (EI): Found 488.3088 [M + H]^+^, C_31_H_41_NO_2_Si requires 488.2907.

*{2-[4-(1,2-Diphenylbut-1-enyl)phenoxy]ethyl}methylamine* (**2b**) {2-[4-(1,2-Diphenylbut-1-enyl)phenoxy]-ethyl}bromide (0.41 g, 1.00 mmol) was dissolved in anhydrous tetrahydrofuran (20 mL) together with methylamine (in a 20 molar equivalent excess), and sealed in a high pressure tube. The reaction was heated to 60 °C while stirring for 48–72 h. Following addition of sodium carbonate/sodium hydrogen carbonate pH 10 buffer solution (50 mL), the mixture were extracted with DCM (3 × 50 mL). The organic phases were combined, dried over sodium sulfate and the solvent evaporated in vacuo to afford a crude product which was then purified via flash chromatography (DCM:MeOH) to afford the product as a brown oil (0.28 g, 77%, *E/Z* = 1:1.1). ^1^H-NMR (CDCl_3_): δ 0.96–1.02 (m, 6H, CH_3_), 2.49–2.58 (m, 10H, CH_2_, CH_3_), 2.98–3.08 (m, 4H, CH_2_N), 3.99–4.23 (m, 6H, NH, CH_2_O), 6.59–7.40 (m, 28H, ArH). ^13^C-NMR (CDCl_3_): 13.25, 28.62, 28.64, 35.16, 35.28, 49.76, 49.89, 65.45, 65.78, 112.96, 113.70, 125.28, 125.67, 126.17, 126.92, 127.39, 127.50, 127.72, 129.03, 129.28, 130.23, 130.40, 131.53, 135.44, 135.94, 137.74, 137.89, 141.03, 141.56, 141.89, 142.88, 143.33, 156.06, 156.92. IR: ν_max_ (KBr) cm^−1^: 3431.2, 1606.0, 1508.0, 1241.3, 1173.2, 1099.5, 1078.8, 1036.9, 938.9, 846.8. HRMS (EI): Found 358.2173 [M + H]^+^, C_25_H_28_NO requires 358.2171.

#### 3.2.2. General Method for the Synthesis of Endoxifen-Acrylic Acid Conjugates **3a**–**3m**

Step (i): A mixture of the required acrylic acid (1 eq., 0.154 mmol), DCC (1 eq., 0.154 mmol, 32 mg), and HOBt (1 eq., 0.154 mmol, 21 mg) was suspended in 3 mL of anhydrous DCM and stirred for 10 min under a nitrogen atmosphere. The endoxifen derivative **2a** (75 mg, 1 eq., 0.154 mmol) was dissolved in 3 mL of anhydrous DCM and slowly added to the mixture via syringe. Reaction was allowed stir for 24–48 h. Reaction was monitored via TLC. The reaction mixture was diluted to 15 mL with anhydrous DCM and filtered to remove DCU. The filtrate was evaporated to dryness under reduced pressure. Purification was not required at this step. *Silyl deprotection*: The residue was dissolved in THF (3 mL) and stirred under nitrogen. A solution of 0.1 M TBAF (2 eq.) was added to the mixture and allowed to stir for 24 h. The mixture was evaporated to dryness under reduced pressure. The residue was dissolved in DCM and was washed with 10% HCl solution. The resulting organic phase was dried over sodium sulphate and evaporated to dryness under vacuum. The material was purified via flash chromatography on silica gel.

Step (ii): A mixture of the required acrylic acid (1.2 eq.), EDC (1.4 eq.), and HOBt (1.4 eq.) was suspended in anhydrous dichloromethane (3 mL) and stirred for 10 min under a nitrogen atmosphere. The endoxifen derivative **2a** (1 eq.) was dissolved in anhydrous dichloromethane (3 mL) and slowly added to the mixture via syringe. Reaction was allowed stir for 16 h. Reaction was monitored via TLC. The reaction mixture was diluted to 15 mL with anhydrous dichloromethane. To this mixture, water (20 mL) was added. The aqueous phase was extracted with DCM (20 mL × 3), brine (50 mL), dried over Na_2_SO_4_ and evaporated to dryness in vacuo to yield the crude product. The material was purified via flash chromatography on silica gel. (DCM:EtOAc). The silyl deprotection was performed as above.

*(E/Z)-3-(Benzo[d][1,3]dioxol-5-yl)-N-(2-(4-((Z)-1-(4-hydroxyphenyl)-2-phenylbut-1-en-1-yl)phenoxy)ethyl)-2-(4-methoxyphenyl)-N-methylacrylamide* (**3k**). Following the general method above, the acrylic acid **1m** (1.2 eq., 176 mg, 0.492 mmol), EDC (1.4 eq., 110 mg, 0.572 mmol) and HOBt (1.4 eq., 77.6 mg, 0.572 mmol) was reacted with endoxifen derivative (1 eq., 200 mg, 0.41 mmol). Purification by flash chromatography over silica gel (DCM:EtOAc, gradient 20:1 to 10:1) afforded the silyl ether as a white solid (261 mg, 83%). HRMS: Found 790.3566 [M + Na]^+^, C_48_H_53_NO_6_NaSi requires 790.3540. The protected compound (142 mg, 0.185 mmol) was dissolved in THF (3 mL) and stirred under nitrogen. A solution of TBAF (1M in THF, 1 eq., 0.185 mL, 0.185 mmol) was added to the mixture and allowed to stir for 1 h. The mixture was evaporated to dryness under reduced pressure and purified via flash chromatography over silica gel (DCM:EtOAc, gradient 6:1 to 3:1) to afford the product as a white solid (109 mg, 91%), m.p. 84–86 °C. ^1^H-NMR (CDCl_3_) δ 6.99–7.24 (m, 17H, Ar-H, C=CH), 6.34–6.88 (m, 25H, Ar-H), 5.87 (s, 4H, CH_2_), 4.00–4.26 (m, 4H, CH_2_), 3.59–3.86 (m, 10H, OCH_3_, CH_2_), 2.96–3.13 (m, 6H, NCH_3_), 2.38–2.54 (m, 4H, CH_2_), 0.87–0.96 (m, 6H, CH_3_). ^13^C-NMR (CDCl_3_) δ 172.8 (C=O), 160.7, 159.3, 158.3, 154.9, 154.0, 148.0, 147.3, 147.3, 147.2, 147.1, 142.6, 140.9, 137.8, 135.7, 135.4, 131.98, 131.96, 130.6, 130.3, 130.2, 130.1, 129.7, 129.7, 129.5, 127.81, 127.8, 125.9, 124.0, 115.1, 114.4, 114.2, 113.9, 113.2, 109.1, 108.1, 108.1, 101.0, 100.2 (OCH_2_O), 65.9 (CH_2_), 60.4 (CH_2_), 55.2 (OCH_3_), 47.6 (CH_2_), 38.7 (NCH_3_), 25.6 (CH_2_), 13.6 (CH_3_). IR: ν_max_ (KBr) cm^−1^: 3239.3, 2929.9, 1735.6, 1605.7, 1508.4, 1244.2, 1036.7, 835.5 HRMS (EI): Found 676.2685 [M + Na]^+^, C_42_H_39_NO_6_Na requires 676.2675.

*(E/Z)-N-(2-(4-((E)-1-(4-Hydroxyphenyl)-2-phenylbut-1-en-1-yl)phenoxy)ethyl)-3-(4-methoxy-3-nitro-phenyl)-N-methyl-2-(3,4,5-trimethoxyphenyl)acrylamide* (**3a**). Following the general method above, the acrylic acid **1a** (1.2 eq., 191 mg, 0.492 mmol), EDC (1.4 eq., 110 mg, 0.572 mmol), and HOBt (1.4 eq., 77.6 mg, 0.572 mmol) was reacted with endoxifen derivative **2a** (1 eq., 200 mg, 0.41 mmol). The crude product was afforded as a brown resin. The material was purified via flash chromatography over silica gel (DCM:EtOAc, gradient 20:1 to 10:1) to afford the product as a white solid. (309 mg, 88%), HRMS: Found 881.3841 [M + Na]^+^, C_50_H_58_N_2_O_9_NaSi requires 881.3809. The product was then deprotected. The protected compound (182 mg, 0.212 mmol) was dissolved in 3 mL THF and stirred under nitrogen environment. A solution of TBAF (1M in THF, 1 eq., 0.212 mL, 0.212 mmol) was added to the mixture and allowed to stir for 1 h. The mixture was evaporated to dryness under reduced pressure. The material was purified via flash chromatography over silica gel (DCM:EtOAc, gradient 6:1 to 3:1) to afford the product as a white solid. (146 mg, 93%), m.p. 77–78 °C. ^1^H-NMR (CDCl_3_) δ 7.58–7.73 (m, 2H, Ar-H), 6.99–7.17 (m, 12H, Ar-H, C=CH), 6.09–6.88 (m, 24H, Ar-H), 4.01–4.28 (m, 4H, CH_2_), 3.53–3.93 (m, 28H, CH_2_, OCH_3_), 2.99–3.20 (m, 6H, NCH_3_), 2.36–2.50 (m, 4H, CH_2_), 0.84–0.96 (m, 6H, CH_3_). ^13^C-NMR (CDCl_3_): δ 172.8 (C=O), 172.7 (C=O), 157.2, 156.3, 155.9, 155.1, 154.1, 153.2, 142.7, 142.6, 141.2, 141.1, 140.9, 137.8, 137.7, 137.1, 136.7, 136.3, 135.7, 135.3, 132.1, 132.0, 130.8, 130.7, 129.7, 127.8, 125.9, 115.1, 114.4, 113.9, 113.1, 113.1, 105.3, 105.3, 66.7, 66.4, 65.1, 64.8, 60.9, 60.5, 56.1, 49.2, 48.3, 48.2, 37.7, 37.6, 35.7, 35.7, 35.2, 35.1, 34.2, 34.1, 32.1, 32.0, 31.6, 31.6, 29.1, 29.0 (CH_2_), 13.7 (CH_3_). IR: ν_max_ (KBr) cm^−1^: 3240.6, 2933.8, 1733.9, 1606.5, 1505.9, 1238.4, 1126.7, 834.2. HRMS (EI): Found 743.2974 [M − H]^−^, C_44_H_43_N_2_O_9_ requires 743.2969.

*(E/Z)-2-(3,5-Dimethoxyphenyl)-N-(2-(4-((Z)-1-(4-hydroxyphenyl)-2-phenylbut-1-en-1-yl)phenoxy)ethyl)-N-methyl-3-(3,4,5-trimethoxyphenyl)acrylamide* (**3b**). Following the general method above, the acrylic acid analogue **1b** was reacted with endoxifen derivative **2a**. The final product was purified via flash chromatography over silica gel (DCM:EtOAc, gradient 6:1 to 3:1) to afford the product as a brown resin (33%). ^1^H-NMR (CDCl_3_): δ 7.17–6.37 (m, 38H, Ar-H, C=CH), 4.25–4.09 (m, 38H, OCH_3_, NCH_2_, OCH_2_), 3.23–3.05 (m, 6H, NCH_3_), 2.52–2.48 (m, 4H, CH_2_), 0.94–0.91 (m, 6H, CH_3_), ^13^C-NMR (CDCl_3_): δ 160.56, 154.86, 153.90, 152.22, 152.20, 140.38, 137.34, 137.30, 131.56, 131.52, 130.20, 130.13, 129.97, 129.23, 127.38, 125.44, 114.67, 113.95, 113.47, 112.71, 106.28, 106.25, 100.06, 60.45 (OCH_3_), 55.34 (OCH_3_), 54.94 (OCH_3_), 53.01, 48.70 (NCH_3_), 33.41 (CH_2_), 28.63 (CH_2_), 28.54 (CH_2_), 25.10 (CH_2_), 24.45 (CH_2_), 13.22 (CH_3_), 13.17 (CH_3_) IR: ν_max_ (KBr) cm^−1^: 3167.5, 2935.3, 1737.4, 1581.6, 1505.6, 1233.1, 1125.0, 1003.7 834.9 HRMS (EI): Found 752.3223 [M + Na]^+^, C_45_H_47_NO_8_Na requires 752.3199.

*(E/Z)-N-(2-(4-((Z)-1-(4-Hydroxyphenyl)-2-phenylbut-1-en-1-yl)phenoxy)ethyl)-N-methyl-2,3-bis(3,4,5-trimethoxyphenyl)acrylamide* (**3c**). Following the general method above, the acrylic acid **1c** (1.2 eq., 198 mg, 0.492 mmol), EDC (1.4 eq., 110 mg, 0.572 mmol), and HOBt (1.4 eq., 77.6 mg, 0.572 mmol) was reacted with endoxifen derivative **2a** (1 eq., 200 mg, 0.41 mmol) to afford the silyl ether which was purified via flash chromatography over silica gel (DCM:EtOAc, gradient 20:1 to 10:1) to afford a white solid. (315 mg, 88%). HRMS: Found 898.4193 [M + H]^+^, C_52_H_63_NO_9_NaSi requires 896.4170. The protected compound (158 mg, 0.18 mmol) was dissolved in 3 mL THF and treated with a solution of TBAF (1 M in THF, 1 eq., 0.18 mL, 0.18 mmol) was added to the mixture and allowed to stir for 1 h. Purifiication by flash chromatography over silica gel (DCM:EtOAc, gradient 6:1 to 3:1) afforded the product as a white solid. (128 mg, 94%), m.p. 77–78 °C. ^1^H-NMR (CDCl_3_) δ 6.97–7.19 (m, 14H, Ar-H, C=CH), 6.30–6.89 (m, 24H, Ar-H), 4.02–4.27 (m, 4H, CH_2_), 3.52–3.95 (m, 40H, OCH_3_), 2.98–3.29 (m, 6H, NCH_3_), 2.37–2.53 (m, 4H, CH_2_), 0.83–0.96 (m, 6H, CH_3_), ^13^C-NMR (CDCl_3_), δ 171.3 (C=O), 155.0, 154.1, 153.4, 152.7, 152.7, 142.6, 142.5, 141.0, 137.8, 137.8, 137.6, 136.6, 135.6, 135.2, 132.0, 131.9, 130.6, 130.6, 130.5, 129.6, 129.6, 127.8, 125.9, 115.0, 114.3, 113.9, 113.1, 106.7, 106.6, 106.1, 106.1, 60.9, 60.82 (OCH_3_), 60.81 (OCH_3_), 60.4 (OCH_3_), 56.1 (OCH_3_), 55.8 (OCH_3_), 53.4, 47.7, 39.0 (NCH_3_) 29.1 (CH_2_), 29.0 (CH_2_), 13.6 (CH_3_), 13.56 (CH_3_) IR: ν_max_ (KBr) cm^−1^: 3167.5, 2935.3, 1737.4, 1581.6, 1505.6, 1233.1, 1125.0, 1003.7 834.9. HRMS (EI): Found 782.3301 [M + Na]^+^, C_46_H_49_NO_9_Na requires 782.3305.

*(E/Z)-2-(3,5-Dimethoxyphenyl)-3-(3-hydroxy-4-methoxyphenyl)-N-(2-(4-((Z)-1-(4-hydroxyphenyl)-2-phenylbut-1-en-1-yl)phenoxy)ethyl)-N-methylacrylamide* (**3d**). Following the general method above, the acrylic acid analogue **1d** was reacted with endoxifen derivative **2a**. Following deprotection, the final product was purified via flash chromatography over silica gel (DCM:EtOAc, gradient 6:1 to 3:1) to afford the product as a brown resin (54%). ^1^H-NMR (CDCl_3_): δ 7.19–6.37 (m, 40H, Ar-H, C=CH), 4.24–3.60 (m, 26H, OCH_3_, O-CH_2_, N-CH_2_), 3.18–3.04 (m, 6H, NCH_3_), 2.53–2.47 (m, 4H, CH_2_), 0.95–0.91 (m, 6H, CH_3_). ^13^C-NMR (CDCl_3_): δ 160.44 (C=O), 156.55, 154.65, 153.69, 146.00, 145.98, 144.60, 144.58, 142.17, 140.49, 140.38, 137.38, 135.30, 135.15, 131.56, 130.18, 129.25, 127.98, 127.38, 127.35, 125.44, 121.62, 115.01, 114.63, 113.90, 113.48, 112.74, 109.71, 106.29, 100.13, 55.40 (OCH_3_), 54.89 (OCH_3_), 53.00 (CH_2_), 48.75 (NCH_3_), 33.41 (OCH_2_), 28.61 (CH_2_CH_3_), 28.55 (CH_2_CH_3_), 25.11 (CH_2_), 24.45 (CH_2_), 13.21 (CH_3_), 13.18 (CH_3_). IR: ν_max_ (KBr) cm^−1^: 3379.5, 2933.2, 1735.1, 1606.3, 1581.7, 1505.9, 1238.9, 1124.6, 1026.5, 833.4. HRMS (EI): Found 708.2925 [M + Na]^+^, C_43_H_43_NO_7_Na requires 708.2937.

*(E/Z)-3-(3,4-Dihydroxyphenyl)-N-(2-(4-((Z)-1-(4-hydroxyphenyl)-2-phenylbut-1-en-1-yl)phenoxy)ethyl)-N-methyl-2-(3,4,5-trimethoxyphenyl)acrylamide* (**3e**). Following the general method above, the acrylic acid **1e** (1.2 eq., 170 mg, 0.492 mmol), EDC (1.4 eq., 110 mg, 0.572 mmol), and HOBt (1.4 eq., 77.6 mg, 0.572 mmol) was reacted with endoxifen derivative **2a** (1 eq., 200 mg, 0.41 mmol) to afford the silyl ether as a which was purified via flash chromatography over silica gel (DCM:EtOAc, gradient 20:1 to 10:1) to afford a white solid. (237 mg, 71%). HRMS: Found 838.3751 [M + Na]^+^, C_49_H_57_NO_8_NaSi requires 838.3751. The silyl ether (157 mg, 0.192 mmol) was dissolved in 3 mL THF and stirred under nitrogen environment. A solution of TBAF (1 M in THF, 1 eq., 0.192 mL, 0.192 mmol) was added to the mixture and allowed to stir for 1 h. The mixture was evaporated to dryness under reduced pressure. The material was purified via flash chromatography over silica gel to afford the product as a white solid. (113 mg, 84%), m.p. 63–69 °C. ^1^H-NMR (CDCl_3_): δ 7.13–6.49 (m, 38H, Ar-H, C=CH), 4.22–3.52 (m, 26H, OCH_3_, O-CH_2_, N-CH_2_), 3.04–2.94 (m, 6H, NCH_3_), 2.47 (m, 4H, CH_2_), 1.32–1.28 (m, 6H, CH_3_). ^13^C-NMR (CDCl_3_): δ 156.62, 154.66, 153.66, 152.88, 145.98, 145.96, 144.60, 142.12, 137.33, 135.20, 132.01, 131.54, 130.22, 130.10, 129.58, 129.25, 128.00, 127.32, 127.30, 125.46, 121.50, 115.10, 114.64, 114.05, 113.40, 112.65, 109.76, 105.58, 65.71, 55.57, 55.44, 48.72, 33.44, 28.62, 25.31, 24.54, 13.22, 13.17. IR: ν_max_ (KBr) cm^−1^: 3250.6, 2930.1, 1735.4, 1605.6, 1605.1, 1244.1, 1037.1, 835.6 HRMS (EI): Found 724.2881 [M + Na]^+^, C_43_H_43_NO_8_Na requires 724.2886.

*(E/Z)-3-(3-Hydroxyphenyl)-N-(2-(4-((Z)-1-(4-hydroxyphenyl)-2-phenylbut-1-en-1-yl)phenoxy)ethyl)-N-methyl-2-(3,4,5-trimethoxyphenyl)acrylamide* (**3f**). Following the general method above, the acrylic acid analogue **1f** was reacted with endoxifen derivative **2a**. The product was purified by flash chromatography over silica gel (DCM:EtOAc, gradient 6:1 to 3:1) to afford the product as a brown resin (45%). ^1^H-NMR (CDCl_3_): δ 7.15–6.38 (m, 40H, Ar-H, C=CH), 4.27–3.45 (m, 26H, OCH_3_, N-CH_2_, O-CH_2_), 3.26–3.07 (m, 6H, NCH_3_), 2.49–2.47 (m, 4H, CH_2_), 0.94–0.91 (m, 6H, CH_3_) ^13^C-NMR (CDCl_3_): δ 171.96, 171.90, 156.64, 155.82, 154.44, 153.53, 152.67, 142.16, 142.65, 140.66, 140.52, 137.24, 137.15, 136.42, 136.28, 135.92, 135.28, 134.91, 131.59, 131.55, 130.25, 130.18, 129.82, 129.50, 129.45, 129.23, 128.87, 127.40, 125.56, 120.82, 120.68, 115.64, 114.94, 114.62, 113.91, 113.40, 112.66, 105.56, 105.47, 105.49, 65.83, 66.46, 55.60, 55.41, 53.01, 47.46, 38.71, 38.63, 28.63, 28.55, 13.20, 13.17. IR: ν_max_ (KBr) cm^−1^: 3250.6, 2930.1, 1735.4, 1605.6, 1605.1, 1244.1, 1037.1, 835.6. HRMS (EI): Found 708.2937 [M + Na]^+^, C_43_H_43_NO_7_Na requires 708.2937.

*(E/Z)-3-(3,4-Dimethoxyphenyl)-N-(2-(4-((Z)-1-(4-hydroxyphenyl)-2-phenylbut-1-en-1-yl)phenoxy)ethyl)-N-methyl-2-(3,4,5-trimethoxyphenyl)acrylamide* (**3g**). Following the general method above, the acrylic acid **1g** (1.2 eq., 184 mg, 0.492 mmol) ), EDC (1.4 eq., 110 mg, 0.572 mmol), and HOBt (1.4 eq., 77.6 mg, 0.572 mmol) was reacted with endoxifen derivative **2a** (1 eq., 200 mg, 0.41 mmol). Purification by flash chromatography over silica gel (DCM:EtOAc, gradient 20:1 to 10:1) afforded a white solid, (300 mg, 87%). HRMS: Found 866.4046 [M + Na]^+^, C_51_H_61_NO_8_NaSi requires 866.4046. The silyl ether (176 mg, 0.21 mmol) was dissolved in THF (3 mL) and stirred under nitrogen. A solution of TBAF (1 M in THF, 1 eq., 0.21 mL, 0.21 mmol) was added to the mixture and allowed to stir for 1 h. The mixture was evaporated to dryness under reduced pressure. The material was purified via flash chromatography over silica gel (DCM:EtOAc, gradient 6:1 to 3:1) to afford the product as a white solid. (139 mg, 91%), m.p. 63–66 °C. ^1^H-NMR (CDCl_3_) δ 6.99–7.19 (m, 13H, Ar-H, C=CH), 6.29–6.87 (m, 25H, Ar-H), 5.89 (br. s., 1H, OH), 4.02–4.26 (m, 4H, CH_2_), 3.51–3.87 (m, 34H, OCH_3_, CH_2_), 2.98–3.24 (m, 6H, NCH_3_), 2.38–2.51 (m, 4H, CH_2_), 0.85–0.95 (m, 6H, CH_3_). ^13^C-NMR (CDCl_3_) δ 171.6 (C=O), 161.0, 154.9, 154.0, 152.7, 152.6, 142.6, 141.0, 137.9, 137.7, 136.7, 135.7, 132.0, 130.6, 130.4, 129.7, 129.6, 127.8, 127.8, 125.9, 115.0, 114.3, 113.9, 113.2, 106.8, 106.7, 100.5, 60.8 (CH_2_), 60.4 (OCH_3_), 55.8 (OCH_3_), 55.4 (OCH_3_), 38.9 (NCH_3_), 25.6 (CH_2_), 13.6 (CH_3_), 13.6 (CH_3_). IR: ν_max_ (KBr) cm^−1^: 3242.0, 2934.9, 2837.9, 2837.6, 1736.1, 1600.2, 1505.9, 1421.4, 1239.7, 1155.4, 833.2 HRMS (EI): Found 752.3210 [M + Na]^+^, C_45_H_47_NO_8_Na requires 752.3199.

*(E/Z)-N-(2-(4-((Z)-1-(4-Hydroxyphenyl)-2-phenylbut-1-en-1-yl)phenoxy)ethyl)-N-methyl-3-(2,3,4-tri-methoxyphenyl)-2-(3,4,5-trimethoxyphenyl)acrylamide* (**3h**). Following the general method above, the acrylic acid analogue **1h** was reacted with endoxifen derivative **2a**. The final product was purified by flash chromatography over silica gel (DCM:EtOAc, gradient 6:1 to 3:1) to afford the product as a brown resin (26%). ^1^H-NMR (CDCl_3_): δ 7.16–6.43 (m, 36H, Ar-H, C=CH), 4.12–3.55 (m, 40H, OCH3, N-CH_2_, O-CH_2_), 3.12–3.10 (m, 6H, NCH_3_), 2.50–2.47 (m, 4H, CH_2_), 0.94–0.91 (t, 6H, J = 7.5Hz CH_3_) ^13^C-NMR (CDCl_3_): δ 170.7 (C=O), 155.0 (C_q_), 154.2 (C_q_), 153.4 (C_q_), 152.7 (C_q_), 152.7 (C_q_), 142.6 (C_q_), 142.5 (C_q_), 141.0 (C_q_), 137.8 (C_q_), 137.8 (C_q_), 137.6 (C_q_), 136.6 (C_q_), 135.6 (C_q_), 135.2 (C_q_), 132.0 (ArC), 131.9 (ArC), 130.6 (ArC), 130.6 (ArC), 130.5 (ArC), 129.6 (ArC), 129.6 (ArC), 127.8 (ArC), 125.9 (ArC), 115.0 (ArC), 114.3 (ArC), 113.9 (ArC), 113.1 (ArC), 106.7 (ArC), 106.6 (ArC), 106.1 (ArC), 106.1 (ArC), 60.9 (CH_2_), 60.82 (OCH_3_), 60.81 (OCH_3_), 60.4 (OCH_3_), 56.1 (OCH_3_), 55.8 (OCH_3_), 53.4, 47.7, 39.0 (NCH_3_) 29.1 (CH_2_), 29.0 (CH_2_), 13.6 (CH_3_), 13.56 (CH_3_). IR: ν_max_ (KBr) cm^−1^: 3167.5, 2935.3, 1737.4, 1581.6, 1505.6, 1233.1, 1125.0, 1003.7, 834.9. HRMS (EI): Found 782.3310 [M + Na]^+^, C_46_H_49_NO_9_Na requires 782.3305.

*(E/Z)-2-(3-Hydroxy-4-methoxyphenyl)-N-(2-(4-((Z)-1-(4-hydroxyphenyl)-2-phenylbut-1-en-1-yl)phenoxy)-ethyl)-N-methyl-3-(3,4,5-trimethoxyphenyl)acrylamide* (**3i**). Following the general method above, the acrylic acid **1i** (1.2 eq., 177 mg, 0.492 mmol)), EDC (1.4 eq., 110 mg, 0.572 mmol) and HOBt (1.4 eq., 77.6 mg, 0.572 mmol) was reacted with endoxifen derivative **2a** (1 eq., 200 mg, 0.41 mmol). Purification by flash chromatography over silica gel (DCM:EtOAc, gradient 20:1 to 10:1) afforded the product as a white solid. (289 mg, 85%), HRMS: Found 852.3920 [M + Na]^+^, C_50_H_59_NO_8_NaSi requires 852.3908. The silyl ether (137 mg, 0.16 mmol) was dissolved in THF (3 mL) and stirred under nitrogen. A solution of TBAF (1 M in THF, 1 eq., 0.16 mL, 0.16 mmol) was added to the mixture and allowed to stir for 1 h. The mixture was evaporated to dryness under reduced pressure. The material was purified by flash chromatography over silica gel (DCM:EtOAc, gradient 6:1 to 3:1) to afford the product as a white solid. (103 mg, 90%), m.p: 81–83 °C. (DCM:EtOAc). ^1^H-NMR (CDCl_3_) δ 6.99–7.19 (m, 15H, Ar-H, C=CH), 5.85–6.88 (m, 23H, Ar-H), 4.05–4.29 (m, 4H, CH_2_), 3.49–3.89 (m, 28H, OCH_3_, CH_2_), 2.91–3.25 (m, 6H, NCH_3_), 2.39–2.50 (m, 4H, CH_2_), 0.85–0.94 (m, 6H, CH_3_). ^13^C-NMR (CDCl_3_) δ 171.2 (C=O), 168.7, 154.9, 154.0, 153.4, 151.7, 151.7, 135.7, 133.3, 132.0, 131.9, 130.7, 130.6, 129.6, 129.6, 128.3, 127.9, 127.8, 125.9, 115.0, 114.3, 113.9, 113.1, 106.1, 106.0, 60.8 (OCH_3_), 60.4 (CH_2_), 56.3 (OCH_3_), 56.2 (OCH_3_), 56.1 (OCH_3_), 55.9 (OCH_3_), 29.04 (CH_2_), 28.97 (CH_2_), 13.6 (CH_3_), 13.57 (CH_3_). IR: ν_max_ (KBr) cm^−1^: 3428.1, 2935.6, 2346.1, 1766.2, 1596.7, 1508.0, 1239.8, 1127.5, 835.0. HRMS (EI): Found 716.3226 [M + H]^+^, C_44_H_46_NO_8_ requires 716.3223.

*(E/Z)-3-(4-Hydroxy-3,5-dimethoxyphenyl)-N-(2-(4-((Z)-1-(4-hydroxyphenyl)-2-phenylbut-1-en-1-yl)-phenoxy)ethyl)-N-methyl-2-(3,4,5-trimethoxyphenyl)acrylamide* (**3j**). Following the general method above, the acrylic acid **1j** (1.2 eq., 194 mg, 0.492 mmol), EDC (1.4 eq., 110 mg, 0.572 mmol) and HOBt (1.4 eq., 77.6 mg, 0.572 mmol) was reacted with endoxifen derivative **2a** (1 eq., 200 mg, 0.41 mmol). Purification by flash chromatography over silica gel (DCM:EtOAc, gradient 20:1 to 10:1) afforded the product as a white solid. (315 mg, 76%), HRMS: Found 882.4022 [M + Na]^+^, C_51_H_61_NO_9_NaSi requires 882.4013. The silyl ether (158 mg, 0.18 mmol) was dissolved in THF (3 mL) and stirred under nitrogen environment. A solution of TBAF (1 M in THF, 1 eq., 0.18 mL, 0.18 mmol) was added to the mixture and allowed to stir for 1 h. The solvent was evaporated under reduced pressure and the residue was purified by flash chromatography over silica gel (DCM:EtOAc, gradient 6:1 to 3:1) to afford the product as a white solid (128 mg, 94%), m.p: 76–79 °C. ^1^H-NMR (CDCl_3_) δ 6.97–7.19 (m, 14H, Ar-H, C=CH), 6.30–6.89 (m, 24H, Ar-H), 4.02–4.27 (m, 4H, CH_2_), 3.52–3.95 (m, 34H, CH_2_, OCH_3_), 2.98–3.29 (m, 6H, NCH_3_), 2.37–2.53 (m, 4H, CH_2_), 0.83–0.96 (m, 6H, CH_3_). ^13^C-NMR (CDCl_3_) δ 171.3 (C=O), 155.0, 154.1, 153.4, 152.7, 152.7, 142.6, 142.5, 141.0, 137.8, 137.8, 137.6, 136.6, 135.6, 135.2, 132.0, 131.9, 130.6, 130.6, 130.5, 129.6, 129.6, 127.8, 125.9, 115.0, 114.3, 113.9, 113.1, 106.7, 106.6, 106.1, 106.1, 60.9 (CH_2_), 60.82 (OCH_3_), 60.81 (OCH_3_), 60.4 (OCH_3_), 56.1 (OCH_3_), 55.8 (OCH_3_), 53.4, 47.7, 39.0 (NCH_3_) 29.1 (CH_2_), 29.0 (CH_2_), 13.6 (CH_3_), 13.56 (CH_3_). IR: ν_max_ (KBr) cm^−1^: 3167.5, 2935.3, 1737.4, 1581.6, 1505.6, 1233.1, 1125.0, 1003.7 834.9. HRMS (EI): Found 768.3133 [M + Na]^+^, C_45_H_47_NO_9_Na requires 768.3149.

*(E/Z)-N-(2-(4-((E)-1-(4-Hydroxyphenyl)-2-phenylbut-1-en-1-yl)phenoxy)ethyl)-N-methyl-3-(5-methyl-thiophen-2-yl)-2-(3,4,5-trimethoxyphenyl)acrylamide* (**3l**). Following the general method above, the acrylic acid **1p** (1.2 eq., 165 mg, 0.492 mmol), EDC (1.4 eq., 110 mg, 0.572 mmol) and HOBt (1.4 eq., 77.6 mg, 0.572 mmol) was reacted with endoxifen derivative **2a** (1 eq., 200 mg, 0.41 mmol). The product was purified by flash chromatography over silica gel (DCM:EtOAc, gradient 20:1 to 10:1) to afford a white solid (184 mg, 56%). HRMS: Found 826.3604 [M + Na]^+^, C_48_H_57_NO_6_NaSiS requires 826.3574. The silyl ether (101 mg, 0.126 mmol) was dissolved in (THF) 3 mL and stirred under nitrogen environment. A solution of TBAF (1 M in THF, 1 eq., 0.126 mL, 0.126 mmol) was added to the mixture and allowed to stir for 1 h. The mixture was evaporated to dryness under reduced pressure. The material was purified via flash chromatography over silica gel (DCM:EtOAc, gradient 6:1 to 3:1) to afford the product as a white solid. (70 mg, 81%), m.p: 83–84 °C. ^1^H-NMR (CDCl_3_): δ 5.82–7.24 (m, 34H, Ar-H, C=CH), 3.99–4.33 (m, 4H, CH_2_), 3.72–3.96 (m, 22H, CH_2_, OCH_3_), 2.97–3.37 (m, 6H, NCH_3_), 2.43–2.56 (m, 4H, CH_2_), 2.37 (d, *J* = 3.26 Hz, 6H, CH_3_), 0.94 (s, 6H, CH_3_). ^13^C-NMR (CDCl_3_): δ 171.3 (C=O), 157.1, 156.3, 154.9, 154.0, 153.6, 153.6, 142.8, 142.7, 142.6, 142.5, 141.1, 141.0, 138.3, 138.2, 137.7, 136.9, 136.0, 135.8, 135.4, 132.3, 132.3, 132.0, 131.4, 131.4, 130.7, 129.7, 127.9, 125.9, 125.7, 125.6, 124.7, 124.7, 115.1, 114.4, 113.9, 113.2, 106.4, 106.3, 61.0 (OCH_3_), 60.5 (CH_2_), 56.3 (OCH_3_), 56.2 (OCH_3_), 38.7 (NCH_3_) (CH_2_), 29.1 (CH_2_), 29.0 (CH_2_), 15.5 (CH_3_), 13.7 (CH_3_), 13.6 (CH_3_). IR: ν_max_ (KBr) cm^−1^: 3255.2, 2961.7, 2932.4, 1735.6, 1605.5, 1580.2, 1505.9, 1238.4, 1120.2, 834.0. HRMS (EI): Found 712.2720 [M + Na]^+^, C_42_H_43_NNaO_6_S requires 712.2709.

*(E/Z)-3-(Furan-3-yl)-N-(2-(4-((E)-1-(4-hydroxyphenyl)-2-phenylbut-1-en-1-yl)phenoxy)ethyl)-N-methyl-2-(3,4,5-trimethoxyphenyl)acrylamide* (**3m**). Following the general method above, the acrylic acid **1q** (1.2 eq., 149 mg, 0.492 mmol), EDC (1.4 eq., 110 mg, 0.572 mmol) and HOBt (1.4 eq., 77.6 mg, 0.572 mmol) was reacted with endoxifen derivative **2a** (1 eq., 200 mg, 0.41 mmol). Purification by flash chromatography over silica gel (DCM:EtOAc, gradient 20:1 to 10:1) afforded the product as a white solid. (89 mg, 32%), HRMS: Found 796.3656 [M + Na]^+^, C_47_H_55_NO_7_NaSi requires 796.3646. The silyl ether (70 mg, 0.09 mmol) was dissolved in THF (3 mL) and stirred under nitrogen. A solution of TBAF (1M in THF, 1 eq., 0.09 mL, 0.09 mmol) was added to the mixture and allowed to stir for 1 h. The solvent was evaporated under reduced pressure. Purification by flash chromatography over silica gel afforded the product as a white solid. (29 mg, 50%), m.p: 81–84 °C. ^1^H-NMR (CDCl_3_) δ 7.55–7.66 (m, 2H, Ar-H, CCH_2_), 5.90–7.47 (m, 40H, Ar-H, C=CH, C=C_2_), 4.00–4.32 (m, 4H, CH_2_), 3.66–3.99 (m, 22H, OCH_3_, CH_2_), 3.02–3.30 (m, 6H, NCH_3_), 2.43–2.56 (m, 4H, CH_2_), 0.90–1.00 (m, 6H, CH_3_). ^13^C-NMR (CDCl_3_): δ 171.7 (C=O), 154.8, 153.9, 153.4, 153.3, 143.6, 142.9, 142.9, 142.6, 142.5, 141.1, 137.9, 137.7, 135.8, 135.6, 135.5, 135.5, 132.0, 130.7, 130.7, 129.7, 127.9, 126.0, 121.3, 121.3, 115.1, 114.4, 113.9, 113.1, 110.0, 106.0, 105.9, 66.2 (OCH_3_), 61.0 (OCH_3_), 60.5 (CH_2_), 56.1 (OCH_3_), 39.1 (NCH_3_), 29.1 (CH_2_), 29.0 (CH_2_), 13.7 (CH_3_), 13.6 (CH_3_). HRMS (EI): Found 682.2791 [M + Na]^+^, C_41_H_41_NNaO_7_ requires 682.2781.

*(E)-N,N-Diethyl-3-(4-methoxyphenyl)-2-(3,4,5-trimethoxyphenyl)acrylamide* (**3q**). The acrylic acid **1n** (1 eq., 0.87 mmol, 0.3 g) was reacted with Mukaiyama’s reagent (3 eq., 2.61 mmol, 0.67 g) in dry DCM (20 mL). After stirring for 5 min, diethylamine (1 eq., 0.87 mmol, 0.06 g) and trimethylamine (5 eq., 4.35 mmol, 0.44 g, 0.60 mL) were added. The reaction was stirred at room temperature for 3 h. After completion the mixture was diluted with DCM and washed with HCl 10% (10 mL), NaHCO_3_ sat (10 mL), water (10 mL), brine (10 mL) and dried over sodium sulphate. The crude material was then purified via flash column chromatography (eluent, DCM: ethylacetate 1:1) to afford the product as a pale yellow oil, (63%, 0.215 g). ^1^H-NMR (CDCl_3_) δ 7.08 (d, *J* = 8.8 Hz, 2H, Ar-H), 6.70 (d, *J* = 8.7 Hz, 2H, Ar-H), 6.55 (s, 1H, CH), 6.54 (s, 2H, Ar-H), 3.82 (s, 3H, OCH_3_), 3.73 (s, 3H, OCH_3_), 3.66 (s, 6H, OCH_3_), 3.39 (s, 4H, CH_2_), 1.06 (d, *J* = 62.4 Hz, 6H, CH_3_). ^13^C-NMR (CDCl_3_) δ 171.10 (C=O), 159.08 (C), 153.22 (2 × C), 137.70 (CH), 136.21 (C), 130.96 (C), 130.73 (2 × CH), 128.46 (C), 127.90 (C), 113.48 (2 × CH), 105.94 (2 × CH), 60.90 (OCH_3_), 56.03 (2 × OCH_3_), 55.17 (OCH_3_), 43.02 (2 × CH_2_), 14.15 (2 × CH_3_). IR: ν_max_ (KBr) cm^−1^: 3368, 2935, 2836, 1668, 1603, 1578, 1507, 1454, 1379, 1275, 1237, 1122, 1028, 1002, 908, 811, 729, 637, 569. HRMS (APCI): Found 400.2113 (M + H) C_23_H_30_NO_5_ requires 400.2118.

*(E)-2-(4-Methoxyphenyl)-3-(3,4,5-trimethoxyphenyl)acrylamide* (**3n**). The acrylic acid **1p** (1 eq., 0.87 mmol, 0.3 g) was reacted with oxalyl chloride (4 eq., 3.48 mmol, 0.44 g, 0.3 mL) and DMF in catalytic amount and stirred in dry DCM at room temperature overnight. The product was concentrated and used for the following step without purification. The residue of the chlorination was redissolved in DCM and reacted with 4 mL of 28% aqueous ammonium hydroxide to afford the product as a yellow oil (50%). ^1^H-NMR (DMSO-*d*_6_), δ 7.37 (s, 1H, CH), 7.20 (s, 1H, H), 7.09 (d, *J* = 8.7 Hz, 2H, Ar-H), 6.99 (d, *J* = 8.8 Hz, 2H, Ar-H), 6.62 (s, 1H, H), 6.30 (s, 2H, Ar-H), 3.74 (s, 3H, OCH_3_), 3.57 (s, 3H, OCH_3_), 3.45 (s, 6H, OCH_3_). ^13^C-NMR (DMSO-*d*_6_) δ 169.25, 159.38 (C), 152.65, 137.96, 135.68, 134.79, 131.20, 130.83, 129.11, 115.00, 107.90, 60.41 (OCH_3_), 55.73 (2 × OCH_3_), 55.65 (OCH_3_). IR: ν_max_ (KBr) cm^−1^: 3375, 2935, 2839, 1672 1593, 1579, 1451, 1424, 1301, 1210, 1234, 1121, 904, 833, 807, 775, 730, 688, 610, 565. HRMS (APCI): Found 344.1485 (M + H) C_19_H_22_NO_5_ requires 344.1492.

*(E)-N,N-diethyl-3-(3-hydroxy-4-methoxyphenyl)-2-(3,4,5-trimethoxyphenyl) acrylamide* (**3o**). Following the general method given above for compound **3q**, the acrylic acid **1l** (1 eq., 1.38 mmol, 0.5 g) was reacted with Mukaiyama’s reagent (3 eq., 4.16 mmol, 1.06 g) in dry DCM (20 mL). After stirring for 5 min, diethylamine (1 eq., 1.38 mmol, 0.1 g) and trimethylamine (5 eq., 6.9 mmol, 0.69 g, 0.96 mL) were added. The reaction was stirred at room temperature for 3 h. After completion, the mixture was diluted with DCM and washed with HCl 10% (10 mL), NaHCO_3_ sat (10 mL), water (10 mL), brine (10 mL) and dried over sodium sulphate., to afford the product as an oil ^1^H-NMR (CDCl_3_) δ 7.26 (s, 1H, Ar-H), 7.21 (d, *J* = 8.7 Hz, 1H, Ar-H), 6.93 (d, *J* = 8.7 Hz, 1H, Ar-H), 6.68 (s, 1H, Ar-H), 6.52 (s, 2H, Ar-H), 3.80 (s, 3H, OCH_3_), 3.76 (s, 3H, OCH_3_), 3.71 (s, 6H, OCH_3_), 3.59 (s, 4H, CH_2_), 1.16 (s, 3H, CH_3_), 0.97 (s, 3H, CH_3_). ^13^C-NMR (CDCl_3_), δ 159.20, 153.43, 149.35, 148.41, 138.93, 138.79, 130.11, 129.46, 126.99, 123.63, 120.79, 113.07, 106.02, 60.97 (OCH_3_), 56.48 (2 × OCH_3_), 56.41 (OCH_3_), 46.58 (2 × CH_2_), 13.89 (CH_3_), 12.68 (CH_3_). IR: ν_max_ (KBr) cm^−1^: 3399, 2935, 1651, 1637, 1578, 1506, 1448, 1413, 1381, 1288, 1238, 1157, 1119, 1017, 971, 808, 773, 569. HRMS (APCI): Found 416.2049 (M + H) C_23_H_30_NO_6_ requires 416.2067.

*(E)-3-(4-Methoxyphenyl)-2-(3,4,5-trimethoxyphenyl)acrylamide* (**3p**). To a solution of acrylic acid **1l** (2 mmol, 0.68 g) in dichloromethane (7 mL), triethylamine (1.2 mL, 8.6 mmol) and thionyl chloride (0.27 mL, 3.72 mmol) were added dropwise. The reaction mixture was stirred at room temperature for 2 h whereupon it was concentrated to dryness under reduce pressure. To 30 mL of a 28% aqueous NH_4_OH solution a solution of the crude residue in dichloromethane (30 mL) was added. The reaction mixture was vigorously stirred at room temperature overnight, then diluted with water and extracted with dichloromethane. The combined organic layers were dried over anhydrous sodium sulfate, filtered and evaporated to dryness under reduce pressure. The residue was purified by flash chromatography (eluent, DCM/ethyl acetate, *v/v* 1:1) to afford the product as white solid (15%, 0.1 g) [71]. ^1^H-NMR (DMSO-*d*_6_) δ 7.43 (s, 1H, Ar-H), 7.20 (s, 1H, NH), 6.99 (d, *J* = 8.8 Hz, 2H, Ar-H), 6.79 (d, *J* = 8.9 Hz, 2H, Ar-H), 6.59 (s, 1H, NH), 6.44 (s, 2H, Ar-H), 3.72 (s, 3H, OCH_3_), 3.70 (s, 3H, OCH_3_), 3.69 (s, 6H, OCH_3_). ^13^C-NMR (CDCl_3_) δ 169.02, 160.20, 154.28, 137.69, 132.23, 130.83, 127.67, 127.10, 113.77, 106.28, 61.04 (OCH_3_), 56.22 (2 × OCH_3_), 55.20 (OCH_3_). IR: ν_max_ (KBr) cm^−1^: 3421, 3298, 3145, 2941, 1673, 1589, 1503, 1452, 1421, 1409, 1368, 1295, 1250, 1234, 1182, 1026, 994, 822, 805, 772, 725, 691, 652. HRMS (EI): Found 344.2194 (M + H) C_19_H_21_NO_5_ requires 344.1498.

#### 3.2.3. General Method for the Synthesis of Endoxifen-Cinnamic Acid/3-Phenylpropanoic Acid Conjugates **5a**–**5d**

Step (A): A mixture of the appropriate cinnamic acid/3-phenylpropanoic acid (1 eq., 0.154 mmol), DCC (1 eq., 0.154 mmol, 32 mg), and HOBt (1 eq., 0.154 mmol, 21 mg) were suspended in anhydrous dichloromethane (3 mL) and stirred for 10 min under a nitrogen. The silyl-protected endoxifen derivative **2a** (75 mg, 1 eq., 0.154 mmol) was dissolved in anhydrous DCM (3 mL) and slowly added to the mixture via syringe. The reaction was allowed stir for 24–48 h and monitored via TLC. The reaction mixture was diluted to 15 mL with anhydrous DCM and filtered to remove DCU. The filtrate was evaporated to dryness under reduced pressure and the residue was dissolved in THF (3 mL) and stirred under nitrogen atmosphere. A solution of 0.1 M TBAF (2 eq.) was added to the mixture and allowed to stir for 24 h. The solvent was evaporated to dryness under reduced pressure. The residue was dissolved in DCM and was washed with 10% HCl solution. The resulting organic phase was dried over sodium sulphate and evaporated to dryness. The material was purified via flash chromatography on silica gel, (eluent: DCM:EtOAc).

Step (B): A mixture of the appropriate cinnamic acid/3-phenylpropanoic acid (1.2 eq), EDC (1.4 eq.), and HOBt (1.4 eq.) were suspended in anhydrous dichloromethane (3 mL) and stirred for 10 min under a nitrogen atmosphere. The protected endoxifen (1 eq.) **2a** was dissolved in anhydrous dichloromethane (3 mL) and slowly added to the mixture via syringe and the reaction was stirred for 16 h and monitored via TLC. The reaction mixture was diluted to 15 mL with anhydrous dichloromethane. To this mixture, water (20 mL) was added. The aqueous phase was extracted with DCM (20 mL × 3), brine (50 mL), dried over Na_2_SO_4_ and evaporated to dryness in vacuo to yield the crude product. The material was purified via flash chromatography on silica gel. (eluent: DCM:EtOAc). The above residue was dissolved in THF (3 mL) and stirred under nitrogen atmosphere. A solution of TBAF (1 M in THF, 1 eq.) was added to the mixture and allowed to stir for 1 h. The mixture was evaporated to dryness under reduced pressure. The material was purified via flash chromatography over silica gel (eluent: DCM:EtOAc) to afford the product.

*(E/Z)-3-(3-Hydroxy-4-methoxyphenyl)-N-(2-(4-((Z)-1-(4-hydroxyphenyl)-2-phenylbut-1-en-1-yl)phenoxy)-ethyl)-N-methylacrylamide* (**5a**). Following the general method above, the cinnamic acid **4a** (1.2 eq., 118 mg, 0.492 mmol), EDC (1.4 eq., 110 mg, 0.572 mmol) and HOBt (1.4 eq., 77.6 mg, 0.572 mmol) was reacted with endoxifen derivative **2a** (1 eq., 200 mg, 0.41 mmol). Purification by flash chromatography over silica gel (DCM:EtOAc, gradient 10:1 to 10:1) affordrd the product as a white solid, (236 mg, 87%). HRMS: Found 686.3317 [M + Na]^+^, C_46_H_49_NO_5_NaSi requires 686.3278. The silyl ether (126 mg, 0.189 mmol) was dissolved in THF (3 mL). A solution of TBAF (1 M in THF, 1 eq., 0.189 mL, 0.189 mmol) was added to the mixture and stirred under nitrogen for 1 h. The solvent was evaporated under reduced pressure. Purification by flash chromatography over silica gel (DCM:EtOAc, gradient 6:1 to 3:1) afforded the product as a white solid. (94 mg, 94%), m.p: 77–78 °C. ^1^H-NMR (CDCl_3_) δ 7.51–7.65 (m, 2H, C=CH), 6.91–7.18 (m, 18H, Ar-H), 6.62–6.87 (m, 13H, Ar-H), 5.79–6.52 (m, 7H, Ar-H, C=CH), 3.94–4.21 (m, 4H, CH_2_), 3.80–3.92 (m, 8H, OCH_3_, 0.5 × CH_2_), 3.75 (s, 2H, CH_2_), 3.03–3.34 (m, 6H, NCH_3_), 2.45 (m, 4H, CH_2_), 0.90 (t, *J* = 7.04 Hz, 6H, CH_3_). ^13^C-NMR (CDCl_3_) δ 171.3 (C=O), 167.2, 167.2, 157.2, 156.3, 155.0, 154.1, 148.2, 145.8, 143.0, 142.7, 142.6, 140.9, 140.8, 137.8, 136.6, 136.2, 135.7, 135.3, 132.0, 132.0, 130.7, 130.6, 129.7, 128.7, 127.8, 125.8, 121.9, 121.6, 115.4, 115.1, 114.3, 113.9, 113.1, 112.6, 112.6, 110.5, 66.8, 66.5, 60.4 (CH_2_), 56.0 (OCH_3_), 48.6, 37.7 (NCH_3_), 35.1 (NCH_3_), 29.01 (CH_2_), 28.99 (CH_2_), 13.6 (CH_3_). IR: ν_max_ (KBr) cm^−1^: 3385.2, 2930.3, 1733.5, 1643.5, 1584.1, 1504.9, 1265.0, 1238.0, 1025.7, 834.0. HRMS (EI): Found 572.2413 [M + Na]^+^, C_35_H_35_NO_5_Na requires 572.2413.

*(E/Z)-3-(3-hydroxy-4-methoxyphenyl)-N-(2-(4-(1-(4-hydroxyphenyl)-2-phenylbut-1-en-1-yl)phenoxy)ethyl)-N-methylpropanamide* (**5b**). Following the general method above, the 3-phenylpropanoic acid **4b** (1.2 eq., 96.5 mg, 0.492 mmol), EDC (1.4 eq., 110 mg, 0.572 mmol) and HOBt (1.4 eq., 77.6 mg, 0.572 mmol) was reacted with endoxifen derivative **2a** (1 eq., 200 mg, 0.41 mmol). Purification by flash chromatography over silica gel (DCM:EtOAc, gradient 20:1 to 10:1) afforded the product as a white solid. (234 mg, 86%). HRMS: Found 688.3438 [M + Na]^+^, C_41_H_51_NO_5_NaSi requires 688.3434. The silyl ether (144 mg, 0.219 mmol) was dissolved in THF (3 mL) and stirred under nitrogen. A solution of TBAF (1M in THF, 1 eq., 0.219 mL, 0.219 mmol) was added to the mixture and stirred for 1 h. The solvent was evaporated and the material was purified via flash chromatography over silica gel (DCM:EtOAc, gradient 6:1 to 3:1) to afford the product as a white solid. (108 mg, 90%), m.p. 78–80 °C. (DCM:EtOAc). ^1^H-NMR (CDCl_3_) δ 7.01–7.20 (m, 12H, Ar-H), 5.57–6.86 (m, 20H, Ar-H), 3.50–4.17 (m, 13H, OCH_3_, CH_2_), 2.40–3.09 (m, 18H, NCH_3_, CH_2_), 0.75–0.98 (m, 6H, CH_3_). ^13^C-NMR (CDCl_3_) δ 172.91 (C=O), 172.85 (C=O), 157.2, 156.3, 156.0, 154.9, 154.0, 145.5, 145.5, 145.5, 145.0, 145.0, 142.7, 142.6, 141.0, 140.9, 137.8, 137.8, 136.6, 136.1, 135.8, 135.7, 135.4, 134.4, 134.4, 132.0, 132.0, 131.9, 130.7, 130.6, 130.6, 129.7, 127.8, 127.8, 125.9, 119.8, 119.8, 115.0, 114.5, 114.5, 114.3, 113.9, 113.1, 113.1, 110.7, 110.7, 66.6 (CH_2_), 66.3 (CH_2_), 64.9 (CH_2_), 60.4 (CH_2_), 55.9 (OCH_3_), 49.1 (CH_2_), 48.2 (CH_2_), 48.1 (CH_2_), 37.6 (NCH_3_), 37.5 (NCH_3_), 35.6 (CH_2_), 35.5 (CH_2_), 35.1 (CH_2_), 35.0 (CH_2_), 34.2, 34.1, 31.0 (CH_2_), 30.7 (CH_2_), 30.6 (CH_2_), 29.0 (CH_2_), 28.98 (CH_2_), 13.6 (CH_3_), 13.6 (CH_3_). IR: ν_max_ (KBr) cm^−1^: 3441.1, 3232.4, 2931.4, 1733.5, 1607.4, 1508.4, 1272.0, 1239.5, 1029.1, 834.8. HRMS (EI): Found 574.2565 [M + Na]^+^, C_35_H_37_NO_5_Na requires 574.2569.

*(E/Z)-N-(2-(4-((Z)-1-(4-Hydroxyphenyl)-2-phenylbut-1-en-1-yl)phenoxy)ethyl)-N-methyl-3-(3,4,5-tri-methoxyphenyl)acrylamide* (**5c**). Following the general method above, the cinnamic acid **4c** (1.2 eq., 117 mg, 0.492 mmol), EDC (1.4 eq., 110 mg, 0.572 mmol) and HOBt (1.4 eq., 77.6 mg, 0.572 mmol) was reacted with endoxifen derivative **2a** (1 eq., 200 mg, 0.41 mmol). Purification by flash chromatography over silica gel (DCM:EtOAc, gradient 20:1 to 10:1) afforded the product as a white solid, (258 mg, 89%). HRMS: Found 730.3569 [M + Na]^+^, C_43_H_53_NO_6_NaSi requires 730.3540. The silyl ether (160 mg, 0.226 mmol) was dissolved in THF (3 mL) and stirred under nitrogen. A solution of TBAF (1 M in THF, 1 eq., 0.226 mL, 0.226 mmol) was added to the mixture stirred for 1 h. The solvent was evaporated under reduced pressure. The material was purified via flash chromatography over silica gel (DCM:EtOAc, gradient 6:1 to 3:1) to afford the product as a white solid. (127 mg, 95%), m.p: 66–68 °C. ^1^H-NMR (CDCl_3_) δ 7.53–7.65 (m, 2H, C=CH), 7.01–7.20 (m, 14H, Ar-H), 6.43–6.89 (m, 18H, Ar-H, C=CH), 3.96–4.25 (m, 4H, CH_2_), 3.74–3.95 (m, 22H, OCH_3_, CH_2_), 3.04–3.39 (m, 6H, CH_3_), 2.46 (q, *J* = 7.04 Hz, 4H, CH_2_), 0.84–0.98 (m, 6H, CH_3_), OH not observed. ^13^C-NMR (CDCl_3_), δ 169.6 (C=O), 153.4, 153.3, 132.0, 130.7, 129.7, 127.8, 125.9, 116.4, 115.0, 114.3, 113.9, 113.1, 105.4, 105.1, 60.9 (CH_2_), 60.4 (OCH_3_), 56.2 (OCH_3_), 56.2 (OCH_3_), 37.9 (NCH_3_), 29.0 (CH_2_), 13.6 (CH_3_) IR: ν_max_ (KBr) cm^−1^: 3236.4, 2934.8, 1735.4, 1647.2, 1583.3, 1506.3, 1240.5, 1089.3, 825.9. HRMS (EI): Found 616.2691 [M + Na]^+^, C_37_H_39_NO_6_Na requires 616.2675.

*N-(2-(4-((E/Z)-1-(4-Hydroxyphenyl)-2-phenylbut-1-enyl)phenoxy)ethyl)-3-(3,4,5-trimethoxyphenyl)-N-methylpropanamide* (**5d**). (i) Following the general method above, the cinnamic acid/3-phenylpropanoic acid analogue **4d** was reacted with endoxifen derivative **2a**. The crude mixture was then taken to the next step without further purification. The final product was purified via flash chromatography over silica gel (DCM:EtOAc, gradient 6:1 to 3:1) to afford the product as a brown resin (29%). (ii) As per general method, the 3-phenylpropanoic acid **4d** (1.2 eq., 118 mg, 0.492 mmol), EDC (1.4 eq., 110 mg, 0.572 mmol), and HOBt (1.4 eq., 77.6 mg, 0.572 mmol) was reacted with endoxifen **2a** (1 eq., 200 mg, 0.41 mmol). The crude product was afforded as a brown resin. The material was purified via flash chromatography over silica gel (DCM:EtOAc, gradient 20:1 to 10:1) to afford the product as a white solid. (264 mg, 92%). HRMS: Found 732.3723 [M + Na]^+^, C_43_H_55_NO_6_NaSi requires 732.3696. The product was then deprotected. The protected compound (156 mg, 0.22 mmol) was dissolved in THF (3 mL) and stirred under nitrogen environment. A solution of TBAF (1 M in THF, 1 eq., 0.22 mL, 0.22 mmol) was added to the mixture and allowed to stir for 1 h. The mixture was evaporated to dryness under reduced pressure. The material was purified via flash chromatography over silica gel (DCM:EtOAc, gradient 6:1 to 3:1) to afford the product as a white solid. (121 mg, 93%), m.p. 73–75 °C. ^1^H-NMR (CDCl_3_): δ 7.18–6.40 (m, 15 H, Ar-H), 4.20–4.13 (m, 1H, CH_2_-O-), 4.05–3.97 (m, 1H, -OCH_2_O-), 3.85–3.83 (m, 9H, OCH_3_), 3.79–3.77 (m, 1H, CH_2_-NCH_3_), 3.70–3.66 (m, 1H, CH_2_-NCH_3_), 3.11 (s, 1H, NCH_3_), 3.08 (s, 1H, NCH_3_), 3.00 (s, 0.5H, NCH_3_), 2.99 (s, 0.5H, NCH_3_), 2.92 (m, 2H, Ar-CH_2_), 2.80–2.58 (m, 2H, -O-CH_2_), 2.52–2.48 (m, 2H, CH_2_-CH_3_), 0.95 (t, 3H, 7.5 Hz, CH_3_), OH not observed. ^13^C-NMR (CDCl_3_): δ 170.27 (C=O), 156.75, 154.48, 152.11, 143.74, 142.31, 140.93, 137.36, 137.54, 136.15, 134.91, 133.88, 131.60, 131.40, 130.23, 130.19, 129.26, 127.38, 125.45, 119.39, 114.59, 144.10, 113.90, 113.42, 112.07, 110.25, 66.18 (OCH_2_), 65.88 (OCH_2_), 56.88 (OCH_3_), 55.64 (OCH_3_), 55.50 (OCH_3_), 53.01, 47.74 (CH_2_), 37.24 (CH_3_N), 37.18 (CH_3_N), 35.17 (CH_2_O), 35.42 (OCH_2_), 33.86 (CH_3_N), 30.73 (CH_2_), 30.52 (CH_2_), 28.46 (CH_2_CH_3_), 13.60 (CH_3_), 13.17 (CH_3_). IR: ν_max_ (KBr) cm^−1^: 3251.9, 2933.8, 1735.3, 1607.2, 1589.8, 1508.4, 1239.2, 1127.4, 833.7. HRMS (EI): Found 618.2691 [M + Na]^+^, C_37_H_41_NO_6_Na requires 618.2691.

*[4-(tert-Butyldimethylsilanyloxy)phenyl]-(4-hydroxyphenyl)methanone* (**7a**). 4,4′-Dihydroxybenzo-phenone (**6**, 6.00 g, 28 mmol) and imidazole (2.094 g, 31 mmol) were dissolved in DMF (20 mL) with stirring. A solution of *tert*-butyldimethylsilyl chloride (4.22 g, 28 mmol) in DMF (20 mL) was added to the mixture over 1 h. The reaction mixture was allowed to stir at room temperature for 16 h followed by the addition of ethyl acetate (100 mL) and 10% hydrochloric acid (50 mL). The organic layer was washed with water (100 mL), brine (100 mL), dried over sodium sulphate and evaporated to dryness in vacuo to yield crude product. The material was purified via flash chromatography on silica gel (*n*-hexane:DCM, 5:1) to afford the product **7a** as a light yellow oil. (3.34 g, 27%). ^1^H-NMR (CDCl_3_): δ 7.76 (dd, 4H, *J* = 6.5 Hz, Ar-H), 6.94 (dd, 4H, *J* = 7.5 Hz, Ar-H), 1.02 (s, 9H, CH_3_), 0.27 (s, 6H, CH_3_). ^13^C-NMR (CDCl_3_): δ 195.66, 160.25, 159.47, 132.41, 132.02, 130.50, 129.37, 119.30, 114.90, 25.16 (CH_3_), 17.80, −4.78 (SiC). IR: ν_max_ (KBr) cm^−1^: 3362.6, 295.9, 2930.0, 2856.9, 1641.6, 1602.3, 1580.3, 1508.1, 1470.5, 1313.0, 1278.1, 1161.1, 913.4, 839.1, 771.9. HRMS (EI): Found 329.1579 [M + H]^+^, C_19_H_25_O_3_Si requires 329.1573. The diprotected benzophenone *bis(4-((tert-butyldimethylsilyl)oxy)phenyl)methanone* was also obtained as a colourless oil (34%). ^1^H-NMR (CDCl_3_): δ 7.75 (d, 4 H, *J* = 8.5 Hz, Ar-H), 6.91 (d, 4H, *J* = 8.5 Hz, Ar-H), 1.02 (s, 18 H, CH_3_), 0.26 (s, 12 H, CH_3_). ^13^C-NMR (CDCl_3_): δ 194.1 (C=O), 159.0, 131.7, 130.8, 119.2, 119.1, 25.2 (CH_2_), 17.8, −4.79 (SiC) IR: ν_max_ (Film) cm^−1^: 2956.0, 2930.8, 2886.7, 2858.8, 1653.1, 1599.1, 1508.0, 1267.5, 1161.2, 1006.4, 909.2. HRMS (EI): Found 437.1937 [M + Na]^+^, C_23_H_34_O_3_Si_2_Na requires 437.1944.

#### 3.2.4. General Method for Cyclofenil Derivatives **8a**–**8e** via McMurry Coupling

Titanium tetrachloride (4.5 eq., 5.2 g, 37.4 mmol, 3 mL) was added via a syringe dropwise to zinc dust (9 eq., 3.58 g, 54.81 mmol) in dry THF (50 mL) and the mixture was refluxed for 2 h in darkness and under nitrogen. The appropriate phenolic ketone **7a** (1 eq.) and cyclic ketone (3 eq.) were dissolved in dry THF (40 mL). This solution was added to the titanium tetrachloride/zinc mixture carefully via a syringe. The reaction mixture was then refluxed for a further 3 h. and then cooled and then diluted with EtOAc (75 mL) and 10% K_2_CO_3_ solution. The mixture was filtered under vacuum and the aqueous layer was extracted with EtOAc (3 × 50 mL). The combined organic layers were washed with 10% K_2_CO_3_ solution (20 mL), water (50 mL) and brine (50 mL) then dried over Na_2_SO_4_, filtered and the solvent was evaporated under reduced pressure to afford the crude product. The material was purified via flash chromatography over silica gel to afford the product.

*4-((4-((tert-Butyldimethylsilyl)oxy)phenyl)(cyclopentylidene)methyl)phenol* (**8a**). Following the general method above, using, TiCl_4_ (4.5 eq., 5.2 g, 27.4 mmol, 3 mL), Zn dust (9 eq., 3.58 g, 54.81 mmol), **7a** (1 eq., 2 g, 6.09 mmol) and cyclopentanone (3 eq., 1.54 g, 18.26 mmol, 1.88 mL). The crude material was purified via flash chromatography over silica gel (*n*-hexane:DCM, 3:1) to afford the product as an orange resin (2.25 g, 97%). ^1^H-NMR (CDCl_3_) δ 7.01–7.11 (m, 4H, (Ar-H)), 6.74–6.82 (m, 4H, (Ar-H)), 2.40 (t, *J* = 7.00 Hz, 4H, (2 × CH_2_)), 1.69 (quin, *J* = 3.50 Hz, 4H, (2 × CH_2_)), 1.01 (s, 9H, (3 × CH_3_)), 0.22 (s, 6H, (2 × CH_3_)). ^13^C-NMR (CDCl_3_) δ 153.4, 153.1, 141.6, 136.4, 135.7, 131.5, 130.0, 129.7, 118.9, 114.3, 32.8 (CH_2_), 26.5 (CH_2_), 25.2 (CH_3_), 17.7 (C(CH_3_)_3_), −4.8 (CH_3_). IR: *ν*_max_ (KBr) cm^−1^: 3400.5 (br.), 2955.5, 2930.6, 2358.3, 1635.33, 1598.3, 1507.4, 1270.0 (br.), 1163.4, 911.7. HRMS (EI): Found 379.2101 [M − H]^−^, C_24_H_31_O_2_Si requires 379.2093.

*4-((4-((tert-Butyldimethylsilyl)oxy)phenyl)(cyclohexaneylidene) methyl) phenol* (**8b**). Following the general method above, TiCl_4_ (4.5 eq., 5.2 g, 27.4 mmol, 3 mL), Zn dust (9 eq., 3.58 g, 54.81 mmol), **7a** (1 eq., 2 g, 6.09 mmol) and cyclohexaneanone (3 eq., 1.79 g, 18.26 mmol, 1.9 mL) were reacted. The crude material was purified via flash chromatography over silica gel (*n*-Hexane:DCM, 3:1) to afford the product as an orange resin (2.1 g, 87%). ^1^H-NMR (CDCl_3_) δ 6.95–7.04 (m, 4H, Ar-H), 6.74–6.79 (m, 4H, Ar-H), 2.23–2.31 (m, 4H, 2 × CH_2_), 1.55–1.69 (m, 6H, 3 × CH_2_), 1.02 (s, 9H, 3 × CH_3_), 0.24 (s, 6H, 2 × CH_3_). ^13^C-NMR (CDCl_3_) δ 153.3, 153.2, 137.8, 136.0, 135.6, 133.1, 130.7, 130.4, 118.8, 114.2, 32.1 (CH_2_), 28.2 (CH_2_), 26.4 (CH_2_), 25.2 (CH_3_), 17.7 (C(CH_3_)_3_), −4.8 (CH_3_). IR: *ν*_max_ (KBr) cm^−1^: 3390.8, 2928.4, 2855.8, 1698.7, 1604.9, 1505.7, 1471, 1463.2, 1447.1, 1258.7, 1167.1, 1099.0, 915.8, 802.1. HRMS (EI): Found 395.2405 [M + H]^+^, C_25_H_35_O_2_Si requires 395.2406.

*4-((4-((tert-Butyldimethylsilyl)oxy)phenyl)(cycloheptylidene)methyl)phenol* (**8c**). Following the general method above, TiCl_4_ (4.5 eq., 5.2 g, 27.4 mmol, 3 mL), Zn dust (9 eq., 3.58 g, 54.81 mmol), **7a** (1 eq., 2 g, 6.09 mmol) and cycloheptanone (3 eq., 2.04 g, 18.26 mmol, 2.15 mL) were reacted. The crude material was purified via flash chromatography over silica gel (*n*-hexane:DCM, 3:1) to afford the product as an orange resin (2.1 g, 84%). ^1^H-NMR (CDCl_3_) δ 6.99–7.06 (m, 4H, Ar-H), 6.73–6.79 (m, 4H, Ar-H), 2.33 (s, 4H, 2 × CH_2_), 1.59 (s, 8H, 4 × CH_2_), 1.00 (s, 9H, 3 × CH_3_), 0.21 (s, 6H, 2 × CH_3_). ^13^C-NMR (CDCl_3_) δ 153.1, 139.1, 136.4, 136.1, 135.8, 130.1, 129.9, 118.9, 114.3, 33.0 (CH_2_), 32.96 (CH_2_), 29.0 (CH_2_), 27.7 (CH_2_), 25.2 (CH_3_), 17.7 (C(CH_3_)_3_), −4.8 (CH_3_). IR: *ν*_max_ (KBr) cm^−1^: 3401.2, 2929.5, 2857.5, 1647.7, 1599.7, 1507.2, 1462.9, 1268.2, 1163.6, 1013.5, 912.9, 840.0, 805.4, 781.4. HRMS (EI): Found 409.2574 [M + H]^+^, C_26_H_37_O_2_Si requires 409.2563.

*4-((4-((tert-Butyldimethylsilyl)oxy)phenyl)(cyclooctylidene)methyl)phenol* (**8d**). Following the general method above, TiCl_4_ (4.5 eq., 5.2 g, 27.4 mmol, 3 mL), Zn dust (9 eq., 3.58 g, 54.81 mmol), **7a** (1 eq., 2 g, 6.09 mmol) and cyclooctanone (3 eq., 2.30 g, 18.26 mmol, 2.4 mL) were reacted. The crude material was purified via flash chromatography over silica gel (*n*-Hexane:DCM, 3:1) to afford the product as an orange resign (2.3 g, 89%). ^1^H-NMR (CDCl_3_). δ 6.99–7.11 (m, 4H, Ar-H), 6.73–6.80 (m, 4H, Ar-H), 2.28 (t, *J* = 6.50 Hz, 4H, 2 × CH_2_), 1.63–1.71 (m, 2H, CH_2_), 1.47–1.62 (m, 8H, 4 × CH_2_), 0.99 (s, 9H, 3 × CH_3_), 0.21 (s, 6H, 2 × CH_3_). ^13^C-NMR (CDCl_3_) δ 153.1, 139.4, 136.7, 136.3, 135.8, 129.8, 129.5, 119.1, 114.4, 32.0 (CH_2_), 26.1 (CH_2_), 25.9 (CH_2_), 25.8 (CH_2_), 25.2 (CH_3_), 17.7 (C(CH_3_)_3_), −4.9 (CH_3_). IR: *ν*_max_ (KBr) cm^−1^: 3345.2 (br.), 2928.7, 2856.5, 1603.4, 1503.5, 1253.8 (br.), 1166.7, 917.0. HRMS (EI): Found 445.2519 [M + Na]^+^, C_27_H_38_NaO_2_Si requires 455.2539.

*4-((4-((tert-Butyldimethylsilyl)oxy)phenyl)(4-methylcyclohexaneylidene) methyl) phenol* (**8e**). Following the general method above, TiCl_4_ (4.5 eq., 5.2 g, 27.4 mmol, 3 mL), Zn dust (9 eq., 3.58 g, 54.81 mmol), **7a** (1 eq., 2 g, 6.09 mmol) and 4-methylcyclohexaneanone (3 eq., 2.05 g, 18.27 mmol, 2.24 mL) were reacted. The crude material was purified via flash chromatography over silica gel (*n*-Hexane:DCM, 3:1) to afford the product as an orange resin (2.15 g, 86%). ^1^H-NMR (CDCl_3_) δ 7.04–7.08 (m, 4H, Ar-H), 6.80–6.86 (m, 4H, Ar-H), 2.69 (d, *J* = 13.1 Hz, 4H, 2 × CH_2_), 2.05 (m, 5H, CH, 2 × CH_2_), 1.09 (s, 9H, 3 × CH_3_), 1.02 (d, 3H, J = 6.5 Hz, CH_3_), 0.30 (s, 6H, 2 × CH_3_). ^13^C-NMR (CDCl_3_) δ 153.4, 153.3, 137.4, 136.2, 135.6, 133.3, 130.7, 130.5, 118.9, 114.4, 32.4 (CH), 29.0 (CH_2_), 26.6 (CH_2_), 25.2 (CH_3_), 17.8 (C(CH_3_)_3_), −4.7 (CH_3_). IR: *ν*_max_ (KBr) cm^−1^: 3369.1, 3031.2, 2919.0, 2855.1, 1704.1, 1603.2. 1505.4, 1471.7, 1362.1, 1253.0, 1167.5, 1099.6, 1007.4, 916.0839, 780.7, 734.2. HRMS (EI): Found 409.2570 [M + H]^+^, C_26_H_37_O_2_Si requires 409.2563.

#### 3.2.5. General Method for the Preparation of Bromoethyl Ethers **9a**–**9e**

The appropriate phenol **8a**–**8e** (1 eq.) was dissolved in 1,2-dibromoethane (~50 eq.) with stirring. Tetrabutylammonium hydrogen sulfate (5 mmol) was added followed by 1 M NaOH solution (50 mL). The biphasic mixture was stirred vigorously at room temperature for 16 h. followed by addition of DCM (100 mL) and NaHCO_3_ solution (100 mL). The aqueous layer was extracted with DCM (2 × 100 mL) and the organic extracts were combined and washed with water (50 mL), brine (50 mL) and dried over Na_2_SO_4_. The solvent was evaporated under reduced pressure, and the residue was purified via flash chromatography over silica gel (eluent: DCM:*n*-hexane) to afford the product.

*(4-((4-(2-Bromoethoxy)phenyl)(cyclopentylidene)methyl)phenoxy)(tert-butyl) dimethylsilane* (**9a**). Following the general method above, phenol **8a** (1 eq., 2.1 g, 5.52 mmol), 1,2-dibromoethane (~50 eq. 52.29 g, 275.8 mmol, 24 mL) and tetrabutylammonium hydrogen sulfate (1.7 g, 5 mmol) were reacted. The crude product was purified via flash chromatography over silica gel (*n*-hexane:DCM, 4:1) to afford the product as a clear resin (2.5 g, 93%). ^1^H-NMR (CDCl_3_) δ 6.67–7.46 (m, 8H, Ar-H), 4.23–4.39 (m, 2H, CH_2_), 3.59–3.75 (m, 2H, CH_2_), 1.46–1.78 (m, 4H, 2 × CH_2_), 1.01 (s, 9H, 3 × CH_3_), 0.23 (s, 6H, 2 × CH_3_). ^13^C-NMR (CDCl_3_) δ 156.9, 154.6, 130.0, 129.7, 128.9, 128.8, 128.6, 120.1, 118.9, 113.6, 113.6, 113.5, 67.3 (CH_2_), 32.8 (CH_2_), 28.7 (CH_2_), 26.6 (CH_2_), 25.2 (CH_3_), 17.7 (C(CH_3_)_3_), −4.8 (CH_3_). IR:*ν*_max_ (KBr) cm^−1^: 2955.0, 2858.7, 1651.1, 1597.8, 1508.3, 1255.3 (br.), 1169.7, 913.6. HRMS (EI): Found 487.1666 [M + H]^+^, C_26_H_36_BrO_2_Si requires 487.1668.

*(4-((4-(2-Bromoethoxy)phenyl)(cyclohexaneylidene)methyl)phenoxy)(tert-butyl)dimethylsilane* (**9b**). Using the general method above, phenol **8b** (1 eq., 2.1 g, 5.32 mmol), 1,2-dibromoethane (50 eq., 52.29 g, 275.8 mmol, 24 mL) and tetrabutylammonium hydrogen sulfate (1.7 g, 5 mmol) were reacted together. The crude product was purified via flash chromatography over silica gel (*n*-hexane:DCM, 4:1) to afford the product as a clear resin (2.44 g, 91%). ^1^H-NMR (CDCl_3_) δ 7.06 (d, *J* = 8.53 Hz, 2H, Ar-H), 6.98 (d, *J* = 8.28 Hz, 2H, Ar-H), 6.85 (d, *J* = 8.78 Hz, 2H, Ar-H), 6.76 (d, *J* = 8.78 Hz, 2H, Ar-H), 4.30 (t, *J* = 6.27 Hz, 2H, CH_2_), 3.66 (t, *J* = 6.27 Hz, 2H, CH_2_), 2.23–2.31 (m, 4H, 2 × CH_2_), 1.59–1.68 (m, 6H, 3 × CH_2_), 1.01 (s, 9H, 3 × CH_3_), 0.23 (s, 6H, 2 × CH_3_). ^13^C-NMR (CDCl_3_) δ 156.3, 153.8, 138.4, 136.8, 136.3, 133.5, 131.0, 130.8, 119.3, 114.1, 67.8 (CH_2_), 32.5 (CH_2_), 29.2 (CH_2_), 28.7 (CH_2_), 26.9 (CH_2_), 25.7 (CH_3_), 18.2 (C(CH_3_)_3_), −4.4 (CH_3_). IR: *ν*_max_ (KBr) cm^−1^: 2930.1, 2857.5, 1654.4, 1600.6, 1508.2, 1471.9, 1254.5, 1168.0, 913.8, 838.9, 804.6, 781.1. HRMS (EI): Found 501.1827 [M + H]^+^, C_27_H_38_BrO_2_Si requires 501.1824.

*(4-((4-(2-Bromoethoxy)phenyl)(cycloheptylidene)methyl)phenoxy)(tert-butyl)dimethylsilane* (**9c**). Following the general method above, phenol **8c** (1 eq., 2.35 g, 5.70 mmol), 1,2-dibromoethane (50 eq. 56.64 g, 298.8 mmol, 26 mL) and tetrabutylammonium hydrogen sulfate (1.7 g, 5 mmol) were reacted together. The crude product was purified via flash chromatography over silica gel (*n*-hexane:DCM, 4:1) to afford the product as a clear resin (2.76 g, 94%). ^1^H-NMR (CDCl_3_) δ 7.09 (d, *J* = 8.53 Hz, 2H, Ar-H), 7.00 (d, *J* = 8.53 Hz, 2H, Ar-H), 6.84 (d, *J* = 8.53 Hz, 2H, Ar-H), 6.75 (d, *J* = 8.53 Hz, 2H, Ar-H), 4.29 (t, *J* = 6.27 Hz, 2H, CH_2_), 3.65 (t, *J* = 6.27 Hz, 2H, CH_2_), 2.32 (s, 4H, 2 × CH_2_), 1.59 (s, 8H, 4 × CH_2_), 0.99 (s, 9H, 3 × CH_3_), 0.20 (s, 6H, 2 × CH_3_). ^13^C-NMR (CDCl_3_) δ 155.7, 153.2, 139.2, 136.7, 136.3, 135.8, 130.1, 129.8, 118.9, 113.6, 67.3 (CH_2_), 32.9 (CH_2_), 29.0 (CH_2_), 28.8 (CH_2_), 27.7 (CH_2_), 25.2 (CH_3_), 17.7 (C(CH_3_)_3_), −4.9 (CH_3_). IR: *ν*_max_ (KBr) cm^−1^: 2929.0, 2856.8, 1602.8, 1507.6, 1471.7, 1254.1, 1168.0, 1016.7, 913.9, 839.4, 781.0. HRMS (TOF-MS): Found 514.1895 [M + H]^+^, C_28_H_39_O_2_BrSi requires 514.1903.

*(4-((4-(2-Bromoethoxy)phenyl)(cyclooctylidene)methyl)phenoxy)(tert-butyl)dimethylsilane* (**9d**). Following the general method above, phenol **8d** (1 eq., 2.33 g, 5.70 mmol), 1,2-dibromoethane (50 eq. 56.64 g, 298.8 mmol, 26 mL) and tetrabutylammonium hydrogen sulfate (1.7 g, 5 mmol) were reacted together. The crude product was purified via flash chromatography over silica gel (*n*-hexane:DCM, 4:1) to afford the product as a clear resin (2.83 g, 94%). ^1^H-NMR (CDCl_3_) δ 7.12 (d, *J* = 8.53 Hz, 2H, Ar-H), 7.03 (d, *J* = 8.03 Hz, 2H, Ar-H), 6.85 (d, *J* = 8.53 Hz, 2H, Ar-H), 6.76 (d, *J* = 8.53 Hz, 2H, Ar-H), 4.28 (t, *J* = 6.27 Hz, 2H, CH_2_), 3.65 (t, *J* = 6.27 Hz, 2H, CH_2_), 2.24–2.32 (m, 4H, 2 × CH_2_), 1.63–1.71 (m, 2H, CH_2_), 1.48–1.63 (m, 8H, 4 × CH_2_), 0.99 (s, 9H, 3 × CH_3_), 0.20 (s, 6H, 2 × CH_3_). ^13^C-NMR (CDCl_3_) δ 155.6, 153.1, 139.6, 137.0, 136.5, 135.7, 129.7, 129.5, 119.1, 113.8, 67.3 (CH_2_), 32.0 (CH_2_), 28.8 (CH_2_), 26.1 (CH_2_), 26.0 (CH_2_), 25.8 (CH_2_), 25.2 (CH_3_), 17.7 (C(CH_3_)_3_), −4.8 (CH_3_). IR: *ν*_max_ (KBr) cm^−1^: 2929.6, 2857.7, 1651.2, 1598.9, 1508.5, 1255.3 (br.), 1168.2, 912.8. HRMS (EI): Found 529.2139 [M + H]^+^, C_29_H_42_BrO_2_Si requires. 529.2137.

*(4-((4-(2-Bromoethoxy)phenyl)(4-methylcyclohexaneylidene) methyl)phenoxy) (tert-butyl)dimethylsilane* (**9e**). Following the general method above, phenol **8e** (1 eq., 2.3 g, 5.63 mmol), 1,2-dibromoethane (50 eq. 56.64 g, 298.8 mmol, 26 mL) and tetrabutylammonium hydrogen sulfate (1.7 g, 5 mmol) were reacted together. The crude product was purified via flash chromatography over silica gel (*n*-hexane:DCM, 4:1) to afford the product as a clear resin (2.61 g, 90%). ^1^H-NMR (CDCl_3_) δ 7.09 (d, *J* = 8.5 Hz, 2H, Ar-H), 7.02 (d, *J* = 8.0 Hz, 2H, Ar-H), 6.87 (d, *J* = 8.5 Hz, 2H, Ar-H), 6.80 (d, *J* = 8.0 Hz, 2H, Ar-H), 4.31 (t, *J* = 6.3 Hz, 2H, CH_2_), 3.67 (t, *J* = 6.3 Hz, 2H, CH_2_), 2.64 (t, *J* = 11.5 Hz, 2H, CH_2_), 1.96–2.03 (m, 2H, CH_2_), 1.82 (d, *J* = 12.0 Hz, 2H, CH_2_), 1.61–1.72 (m, 1H, CH), 1.08–1.18 (m, 2H, CH_2_), 1.04 (s, 9H, CH_3_), 0.98 (d, *J* = 6.5 Hz, 3H, CH_3_), 0.21 (s, 6H, 2 × CH_3_). ^13^C-NMR (CDCl_3_) δ 155.9, 153.4, 137.6, 136.3, 135.9, 133.2, 130.6, 130.4, 118.8, 118.8, 113.6, 69.3 (CH_2_), 36.5 (CH_2_), 32.4 (CH), 31.4 (CH_2_), 28.8 (CH_2_), 25.3 (CH_3_), 17.77 (C(CH_3_)_3_), −4.8 (CH_3_). IR: *ν*_max_ (KBr) cm^−1^: 2951.7, 2927.8, 2857.0, 1604.5, 1507.3, 1472.0, 1457.4, 1254.1, 1168.2, 1017.15, 914.7, 839.7, 804.7, 780.5. HRMS (EI): Found 515.2006 [M + H]^+^, C_28_H_40_O_2_SiBr requires 515.1981.

#### 3.2.6. General Method for Preparation of Amines **10a**–**10e**

Methylamine (2 M in THF) (20 eq.) was added to the alkyl bromide compound **9a**–**9e** and sealed in a high pressure tube. The reaction mixture was heated to 60 °C while stirring for 48 h. The reaction mixture was allowed sufficient time to cool, allowing the internal pressure to decrease prior to opening the pressure tube. The solvent was evaporated under reduced pressure. The oil was dissolved in DCM (30 mL) and was washed with a pH10 aq. solution (30 mL) which was then extracted with DCM (2 × 30 mL). The organic layers were combined and washed with water (50 mL), brine (50 mL) and dried over Na_2_SO_4_. The solvent was evaporated under reduced pressure to obtain a brown oil. The crude material was purified via flash chromatography on silica gel. (DCM:EtOAc).

*2-(4-((4-((tert-Butyldimethylsilyl)oxy)phenyl)(cyclopentylidene)methyl)phenoxy)-N-methylethanamine* (**10a**). Following the general method above, **9a** (1 g, 2.05 mmol) and methylamine (20 eq., 41.02 mmol, 20 mL) were reacted together. The crude material was purified via flash chromatography on silica gel (DCM:EtOAc, 2:1) to afford a brown resin (737 mg, 82%). ^1^H-NMR (CDCl_3_) δ 7.10 (d, *J* = 8.53 Hz, 2H, Ar-H), 7.03 (d, *J* = 8.53 Hz, 2H, Ar-H), 6.86 (d, *J* = 8.53 Hz, 2H, Ar-H), 6.76 (d, *J* = 8.03 Hz, 2H, Ar-H), 4.12–4.19 (m, 2H, CH_2_), 3.03–3.16 (m, 2H, CH_2_), 2.58 (s, 3H, CH_3_), 2.34–2.44 (m, 4H, 2 × CH_2_), 1.63–1.73 (m, 4H, 2 × CH_2_), 1.00 (s, 9H, 3 × CH_3_), 0.20 (s, 6H, 2 × CH_3_). ^13^C-NMR (CDCl_3_) δ 156.0 (C-O-CH_2_), 153.2, 141.8, 136.4, 136.3, 131.4, 129.9, 129.7, 118.9, 113.4, 65.1 (CH_2_), 49.5 (CH_2_), 32.8 (CH_3_), 26.49 (CH_2_), 26.47 (CH_2_), 25.2 (CH_3_), 17.7 (C(CH_3_)_3_), −4.8 (CH_3_). IR: *ν*_max_ (KBr) cm^−1^: 3343.1 (br.), 2955.1, 2930.6, 2857.7, 1660.9, 1604.1, 1508.4, 1254.1 (br.), 1168.9, 915.4. HRMS (EI): Found 438.2838 [M + H]^+^, C_27_H_40_NO_2_Si requires 438.2828.

*2-(4-((4-((tert-Butyldimethylsilyl)oxy)phenyl)(cyclohexaneylidene)methyl)phenoxy)-N-methylethanamine* (**10b**). Following the general method above, **9b** (1 g, 2.0 mmol) and methylamine (20 eq., 41.02 mmol, 20 mL) were reacted together. The crude material was purified via flash chromatography on silica gel (DCM:EtOAc, 2:1) to afford a brown resin (781 mg, 86%). ^1^H-NMR (CDCl_3_) δ 7.03 (d, *J* = 8.5 Hz, 2H, Ar-H), 6.96 (d, *J* = 8.5 Hz, 2H, Ar-H), 6.84 (d, *J* = 8.5 Hz, 2H, Ar-H), 6.74 (d, *J* = 8.5 Hz, 2H, Ar-H), 4.13–4.17 (m, 2H, CH_2_), 3.08 (d, *J* = 6.5 Hz, 2H, CH_2_), 2.58 (s, 3H, CH_3_), 2.24 (d, *J* = 5.0 Hz, 4H, 2 × CH_2_), 1.56–1.63 (m, 6H, 3 × CH_2_), 0.99 (s, 9H, CH_3_), 0.21 (s, 6H, CH_3_). ^13^C-NMR (CDCl_3_) δ 156.1, 153.3, 137.9, 136.0, 135.8, 133.0, 130.5, 130.3, 118.8, 113.4, 65.1 (CH_2_), 49.5 (CH_2_), 34.7 (NCH_3_), 32.1 (CH_2_), 32.0 (CH_2_), 28.2 (CH_2_), 26.4 (CH_3_), 17.71 (C(CH_3_)_3_), −4.83 (CH_3_). IR: *ν*_max_ (KBr) cm^−1^: 3435.1, 2928.5, 2854.3, 1606.2, 1507.9, 1471.3, 1255.0, 1168.7, 915.1, 835.8, 778.9. HRMS (EI): Found 452.2982 [M + H]^+^, C_28_H_42_NO_2_Si requires 452.2985.

*2-(4-((4-((tert-Butyldimethylsilyl)oxy)phenyl)(cycloheptylidene)methyl)phenoxy)-N-methylethanamine* (**10c**). Following the general method above, **9c** (1 g, 1.94 mmol) and methylamine (~20 eq., 41.02 mmol, 20 mL) were reacted together. The crude material was purified via flash chromatography over silica gel (DCM:EtOAc, 2:1) to afford a brown resin (813 mg, 90%). ^1^H-NMR (CDCl_3_) δ 7.07 (d, *J* = 8.8 Hz, 2H, Ar-H), 7.00 (d, *J* = 8.3 Hz, 2H, Ar-H), 6.83 (d, *J* = 8.3 Hz, 2H, Ar-H), 6.74 (d, *J* = 8.3 Hz, 2H, Ar-H), 4.08 (d, *J* = 6.5 Hz, 2H, CH_2_), 3.61 (d, *J* = 6.5 Hz, 2H, CH_2_), 2.99 (s, 1H, NH), 2.52 (s, 3H, CH_3_), 2.29–2.33 (m, 4H, 2 × CH_2_), 1.55–1.64 (m, 8H, 4 × CH_2_), 0.99 (s, 9H, CH_3_), 0.20 (s, 6H, CH_3_). ^13^C-NMR (CDCl_3_) δ 156.7, 153.6, 139.5, 136.8, 136.7, 136.4, 132.0, 130.4, 130.3, 128.5, 120.0, 119.3, 113.8, 113.6, 65.1 (CH_2_), 49.5 (CH_2_), 33.5 (NCH_3_), 33.4 (CH_2_), 29.4 (CH_2_), 28.2 (CH_2_), 28.1 (CH_2_), 25.7 (CH_3_), 18.1 (C(CH_3_)_3_), −4.8 (CH_3_). IR: *ν*_max_ (KBr) cm^−1^: 3436.1, 2928.1, 2855.9, 1604.3, 1507.1, 1471.7, 1253.4, 1171.7, 1045.2, 914.6, 839.2, 805.0, 780.6. HRMS (EI): Found 446.3152 [M + H]^+^, C_29_H_44_NO_2_Si requires 446.3141.

*2-(4-((4-((tert-Butyldimethylsilyl)oxy)phenyl)(cyclooctylidene)methyl)phenoxy)-N-methylethanamine* (**10d**). Following the general method above, **9d** (1 g, 1.89 mmol) and methylamine (20 eq., 41.02 mmol, 20 mL) were reacted together. The crude material was purified via flash chromatography over silica gel (DCM:EtOAc, 2:1) to afford a brown resin (761 mg, 84%). ^1^H-NMR (CDCl_3_) δ 7.06–7.15 (m, 2H, Ar-H), 6.98–7.06 (m, 2H, Ar-H), 6.85–6.93 (m, 2H, Ar-H), 6.71–6.79 (m, 2H, Ar-H), 4.16–4.26 (m, 2H, CH_2_), 3.10–3.24 (m, 2H, CH_2_), 2.60 (s, 3H, CH_3_), 2.20–2.32 (m, 4H, 2 × CH_2_), 1.62–1.73 (m, 2H, CH_2_), 1.45–1.62 (m, 8H, 4 × CH_2_), 0.99 (s, 9H, 3 × CH_3_), 0.19 (s, 6H, 2 × CH_3_). ^13^C-NMR (CDCl_3_) δ 155.4 (C-O-CH_2_), 153.1, 139.6, 137.1, 136.5, 135.6, 129.6, 129.4, 119.1, 113.8, 63.3 (CH_2_), 48.5 (CH_2_), 33.6 (CH_3_), 32.0 (CH_2_), 26.0 (CH_2_), 25.9 (CH_2_), 25.7 (CH_2_), 25.2 (CH_3_), 17.7 (C(CH_3_)_3_), −4.8 (CH_3_). IR: *ν*_max_ (KBr) cm^−1^: 3413.8 (br.), 2927.7, 2856.0, 1643.2, 1605.9, 1506.7, 1241.1 (br.), 1168.5, 835.8. HRMS (EI): Found 480.3301 [M + H]^+^, C_30_H_46_NO_2_Si requires 480.3298.

*2-(4-((4-((tert-Butyldimethylsilyl)oxy)phenyl)(4-methylcyclohexaneylidene)methyl)phenoxy)-N-methyl-ethanamine* (**10e**). Following the general method above, **9e** (1 g, 1.94 mmol) and methylamine (~20 eq., 41.02 mmol, 20 mL) were reacted together. The crude material was purified via flash chromatography on silica gel (DCM:EtOAc, 2:1) to afford a brown resin (722 mg, 80%). ^1^H-NMR (CDCl_3_) δ 7.04 (d, *J* = 7.0 Hz, 2H, Ar-H), 6.97 (d, *J* = 8.5 Hz, 2H, Ar-H), 6.84 (d, *J* = 8.5 Hz, 2H, Ar-H), 6.75 (d, *J* = 8.5 Hz, 2H, Ar-H), 4.09 (t, *J* = 4.5 Hz, 2H, CH_2_), 3.01 (s, 2H, CH_2_), 2.75 (s, 1H, NH), 2.54–2.62 (m, 5H, CH_3_, CH_2_), 1.95 (t, *J* = 12.5 Hz, 2H, CH_2_), 1.78 (d, 2H, CH_2_), 1.60–1.64 (m, 1H, CH), 1.09 (q, *J* = 12.0 Hz, 2H, CH_2_), 1.00 (s, 9H, CH_3_), 0.94 (d, *J* = 6.6 Hz, 3H, CH_3_), 0.22 (s, 6H, CH_3_). ^13^C-NMR (CDCl_3_) δ 156.5, 153.3, 137.4, 135.9, 135.8, 133.2, 130.5, 130.4, 130.4, 118.8, 113.3, 66.1 (CH_2_), 50.2 (CH_2_), 36.4 (CH_2_), 35.6 (NCH_3_), 32.4 (CH), 31.3 (CH_2_), 25.2 (CH_3_), 17.7 (C(CH_3_)_3_), −4.8 (CH_3_). IR: *ν*_max_ (KBr) cm^−1^: 3435.6, 2946.9, 2927.7, 2856.3, 1605.4, 1507.9, 1457.6, 1471.9, 1253.3, 1168.5, 1045.1, 915.2, 838.1, 805.0, 780.0. HRMS (EI): Found 466.3156 [M + H]^+^, C_29_H_44_NO_2_Si requires 466.3141.

#### 3.2.7. General Method for Preparation of Cyclofenil Derivatives **11a**–**11e**

The appropriate silyl ether **10a**–**10e** was dissolved in THF (3 mL) while stirring under N_2_. An equimolar quantity of TBAF (1 M in THF) was added and the mixture was allowed to stir for 30 min. The reaction was monitored via TLC. The solvent was evaporated to dryness. The residue was redissolved in DCM (20 mL) and washed with a 10% HCl solution (10 mL). The organic phase was dried over Na_2_SO_4_ and evaporated to dryness in vacuo. The residue is purified via flash chromatography on silica gel to afford the products.

*4-(Cyclopentylidene(4-(2-(methylamino)ethoxy)phenyl)methyl)phenol* (**11a**). Following the general deprotection method, the cyclofenil derivative **10a** (200 mg, 0.457 mmol) and TBAF (1 M in THF) (0.457 mmol, 0.457 ml) were reacted together in THF (3 mL). The residue was purified via flash chromatography on silica gel (DCM:MeOH, gradient 50:1 to 40:1) to afford the product as a pale pink solid (107 mg, 73%), m.p. 160–163 °C. ^1^H-NMR (CDCl_3_) δ 9.23–9.42 (m, 1H, OH), 7.03 (d, *J* = 8.53 Hz, 2H, Ar-H), 6.92 (d, *J* = 8.53 Hz, 2H, Ar-H), 6.86 (d, *J* = 8.53 Hz, 2H, Ar-H), 6.68 (d, *J* = 8.53 Hz, 2H, Ar-H), 4.00 (t, *J* = 5.52 Hz, 2H, CH_2_), 2.84 (t, *J* = 5.27 Hz, 2H, CH_2_), 2.35 (s, 3H, NCH_3_), 2.26–2.34 (m, 4H, 2 × CH_2_), 1.54–1.69 (m, 4H, 2 × CH_2_). ^13^C-NMR (CDCl_3_) δ 157.1, 155.9, 140.9, 136.3, 134.5, 132.4, 130.3, 115.2, 114.3, 67.2 (CH_2_), 50.6 (CH_2_), 36.4 (NCH_3_), 33.2 (CH_2_), 26.9 (CH_2_). IR:*ν*_max_ (KBr) cm^−1^: 3438.3 (br.), 2952.55, 2864.3, 2591.1, 1608.2, 1507.1, 1268.9, 1235.6, 1053.8, 834.8. HRMS (EI): Found 324.1950 [M + H]^+^, C_21_H_26_NO_2_ requires 324.1964.

*4-(Cyclohexaneylidene(4-(2-(methylamino)ethoxy)phenyl)methyl)phenol* (**11b**). Following the general deprotection method above, the cyclofenil derivative **10b** (200 mg, 0.442 mmol) and TBAF (1 M in THF) (0.442 mmol, 0.442 ml) were reacted together in THF (3 mL). The residue was purified via flash chromatography on silica gel (DCM:MeOH, gradient 50:1 to 40:1) to afford the product as a white solid (116 mg, 78%), m.p. 169–170 °C. ^1^H-NMR (DMSO-*d*_6_) δ 6.95 (d, *J* = 8.53 Hz, 2H, Ar-H), 6.77–6.88 (m, 4H, Ar-H), 6.67 (d, *J* = 8.53 Hz, 2H, Ar-H), 3.98 (t, *J* = 5.52 Hz, 2H, CH_2_), 2.80 (t, *J* = 5.65 Hz, 2H, CH_2_), 2.33 (s, 3H, NCH_3_), 2.09–2.23 (m, 4H, 2 × CH_2_), 1.46–1.64 (m, 6H, 3 × CH_2_) (OH not observed). ^13^C-NMR (DMSO-*d*_6_) δ 157.3, 157.0, 137.0, 135.9, 134.2, 133.2, 130.9, 130.8, 115.3, 114.3, 67.5 (CH_2_), 50.8 (CH_2_), 36.6 (NCH_3_), 32.4 (CH_2_), 28.6 (CH_2_), 23.5 (CH_2_). IR: *ν*_max_ (KBr) cm^−1^: 3438.2 (br.), 2930.0, 2847.3, 2660.1, 2591.11, 1607.8, 1508.4, 1465.7, 1302.6, 1273.0, 1168.4, 1041.8, 833.7. HRMS (EI): Found 338.2104 [M + H]^+^, C_22_H_28_NO_2_ requires 338.2120.

*4-(Cycloheptylidene(4-(2-(methylamino)ethoxy)phenyl)methyl)phenol* (**11c**). Following the general deprotection method above, the cyclofenil derivative **10c** (200 mg, 0.429 mmol) and TBAF (1 M in THF) (0.429 mmol, 0.429 mL) were reacted together in THF (3 mL). The residue was purified via flash chromatography on silica gel (DCM:MeOH, gradient 50:1 to 40:1) to afford the product as a pale pink solid (122 mg, 81%), m.p. 169–171 °C. ^1^H-NMR (DMSO-*d*_6_) δ 7.00 (d, *J* = 8.78 Hz, 2H, Ar-H), 6.80–6.95 (m, 4H, Ar-H), 6.67 (d, *J* = 8.53 Hz, 2H, Ar-H), 3.97 (t, *J* = 5.65 Hz, 2H, CH_2_), 2.80 (t, *J* = 5.52 Hz, 2H, CH_2_), 2.33 (s, 3H, NCH_3_), 2.18–2.27 (m, 4H, 2 × CH_2_), 1.46–1.63 (m, 8H, 4 × CH_2_). ^13^C-NMR (DMSO-*d*_6_) δ 157.2, 156.0, 138.4, 137.1, 136.4, 134.5, 130.3, 130.3, 115.2, 114.3, 67.5 (CH_2_), 50.8 (CH_2_), 36.6 (NCH_3_), 33.3 (CH_2_), 29.2 (CH_2_), 28.0 (CH_2_), 23.5 (CH_2_). IR: *ν*_max_ (KBr) cm^−1^: 3440.6 (br.), 2929.7, 2849.4, 2575.8, 1607.4, 1505.9, 1463.3, 1274.6, 1240.8, 1168.6, 1045.2, 828.8. HRMS (EI): Found 352.2261 [M + H]^+^, C_23_H_30_NO_2_ requires 352.2277.

*4-(Cyclooctylidene(4-(2-(methylamino)ethoxy)phenyl)methyl)phenol* (**11d**). Following the general deprotection method above, the cyclofenil **10d** (200 mg, 0.417 mmol) and TBAF (1 M in THF) (0.417 mmol, 0.417 mL) were reacted together in THF (3 mL). The residue was purified via flash chromatography on silica gel (DCM:MeOH, gradient 50:1 to 40:1) to afford the product as a clear oil (119 mg, 78%). ^1^H-NMR (DMSO-*d*_6_) δ 7.02 (d, *J* = 8.53 Hz, 2H, Ar-H), 6.80–6.93 (m, 4H, Ar-H), 6.68 (d, *J* = 8.53 Hz, 2H, Ar-H), 3.96 (t, *J* = 5.65 Hz, 2H, CH_2_), 2.80 (t, *J* = 5.77 Hz, 2H, CH_2_), 2.32 (s, 3H, NCH_3_), 2.13–2.26 (m, 4H, 2 × CH_2_), 1.40–1.68 (m, 10H, 5 × CH_2_). ^13^C-NMR (DMSO-*d*_6_) δ 157.1, 156.8, 138.5, 137.2, 136.8, 134.1, 130.0, 129.8, 115.5, 114.5, 67.5 (CH_2_), 50.8 (CH_2_), 36.6 (NCH_3_), 32.4 (CH_2_), 26.4 (CH_2_), 26.2 (CH_2_), 23.5 (CH_2_). IR: *ν*_max_ (KBr) cm^−1^: 3438.1 (br.), 2932.3, 2871.9, 2852.2, 2640.4, 2566.5, 1607.9, 1587.1, 1505.9, 1455.8, 1274.9, 1239.5, 1168.1, 1046.6, 838.9. HRMS (EI): Found 366.2414 [M + H]^+^, C_24_H_32_NO_2_ requires 366.2433.

*4-((4-(2-(Methylamino)ethoxy)phenyl)(4-methylcyclohexaneylidene)methyl)phenol* (**11e**). Following the general deprotection method above, the cyclofenil derivative **10e** (200 mg, 0.429 mmol) and TBAF (1 M in THF) (0.429 mmol, 0.429 mL) were reacted together in THF (3 mL). The residue was purified via flash chromatography on silica gel (DCM:MeOH, gradient 50:1 to 40:1) to afford the product as a pale pink solid (124 mg, 82%), m.p: 74–76 °C. ^1^H-NMR (DMSO-*d*_6_) δ 6.94 (d, *J* = 8.50 Hz, 2H, Ar-H), 6.78–6.88 (m, 4H, Ar-H), 6.68 (d, *J* = 8.53 Hz, 2H, Ar-H), 3.98 (t, *J* = 5.52 Hz, 2H, CH_2_), 2.80 (t, *J* = 5.40 Hz, 2H, CH_2_), 2.48 (t, *J* = 16.10 Hz, 2H, CH_2_), 2.33 (s, 3H, NCH_3_), 1.90 (dt, *J* = 3.14, 12.86 Hz, 2H, CH_2_), 1.72 (d, *J* = 11.80 Hz, 2H, CH_2_), 1.51–1.63 (m, 3H, CH_2,_ CH), 0.90 (d, *J* = 6.27 Hz, 3H, CH_3_). ^13^C-NMR (DMSO-*d*_6_) δ 157.3, 156.6, 136.7, 135.9, 134.3, 133.6, 130.9, 130.8, 115.2, 114.3, 67.5 (CH_2_), 50.8 (CH_2_), 36.6 (NCH_3_), 32.7 (CH), 31.7 (CH_2_), 23.5 (CH_2_), 22.4 (CH_3_). IR:*ν*_max_ (KBr) cm^−1^: 3439.7 (br.), 2945.8, 2913.1, 2887.4, 2866.9, 1607.06, 1505.9, 1456.2, 1270.3, 1234.5, 1168.2, 1043.6, 830.4. HRMS (EI): Found 352.2264 [M + H]^+^, C_23_H_30_NO_2_ requires 352.2277.

#### 3.2.8. General Method for the Synthesis of OTBDMS Cyclofenil-Acrylic Acid Conjugates **12a**–**12e**

A mixture of the required acrylic acid **1l** (1.2 eq), EDC (1.4 eq.) and HOBt (1.4 eq.) was suspended in anhydrous dichloromethane (3 mL) and stirred for 10 min under a nitrogen atmosphere. The appropriate cyclofenil derivative **10a**–**10e** (1 eq.) was dissolved in anhydrous dichloromethane (3 mL) and slowly added to the mixture via syringe. The reaction was allowed stir for 16 h. The reaction was monitored via TLC. The reaction mixture was diluted to 15 mL with anhydrous dichloromethane. To this mixture, water (20 mL) was added. The aqueous phase was extracted with DCM (20 mL × 3), brine (50 mL), dried over Na_2_SO_4_ and evaporated to dryness in vacuo to yield the crude product. The material was purified via flash chromatography on silica gel using DCM:EtOAc as eluent.

(E)-N-(2-(4-((4-((tert-Butyldimethylsilyl)oxy)phenyl)(cyclopentylidene)methyl)phenoxy)ethyl)-3-(3-hydroxy-4-methoxyphenyl)-N-methyl-2-(3,4,5-trimethoxy phenyl) acrylamide (**12a**). The acrylic acid **1l** (1.2 eq., 196 mg, 0.54 mmol), EDC (1.4 eq., 120 mg, 0.63 mmol), and HOBt (1.4 eq., 85 mg, 0.63 mmol) were reacted with protected cyclofenil derivative **10a** (1 eq., 200 mg, 0.45 mmol) following the general method above. The product was obtained as a brown resin. The material was purified via flash chromatography on silica gel (DCM:EtOAc, gradient 15:1 to 10:1) to afford the product as a yellow solid (284 mg, 81%), m.p. 87–89 °C, which was used immediately in the following reaction without further purification. ^1^H-NMR (CDCl_3_) δ 6.97–7.16 (m, 4H, Ar-H, C=CH), 6.52–6.88 (m, 10H, Ar-H), 4.25 (m, 1H, 0.5 × OCH_2_), 3.60–3.92 (m, 15H, 0.5 × OCH_2_, NCH_2_, 4 × OCH_3_), 3.08–3.23 (m, 3H, NCH_3_), 2.40 (s, 4H, 2 × CH_2_), 1.69 (s, 4H, 2 × CH_2_), 1.00 (s, 9H, 3 × CH_3_), 0.22 (s, 6H, 2 × CH_3_). ^13^C-NMR (CDCl_3_) **δ** 169.0, 153.2, 152.8, 145.8, 144.6, 141.8, 129.9, 129.7, 118.9, 115.0, 113.2, 109.7, 105.5, 66.0 (CH_2_), 60.5 (OCH_3_), 55.4 (OCH_3_), 38.6 (CH_2_), 32.8 (CH_2_), 26.5 (CH_2_), 25.2 (CH_3_), 17.7 (C(CH_3_)_3_), −4.8 (CH_3_). IR: *ν*_max_ (KBr) cm^−1^: 3350.9 (br.), 2954.2, 2936.7, 2859.3, 1603.9, 1581.5, 1505.9, 1463.8, 1242.5 (br.), 1127.1, 912.2. HRMS (EI): Found 780.3922 [M + H]^+^, C_46_H_58_NO_8_Si requires 780.3932.

*(E)-N-(2-(4-((4-((tert-Butyldimethylsilyl)oxy)phenyl)(cyclohexaneylidene)methyl)phenoxy)ethyl)-3-(3-hydroxy*-*4-methoxyphenyl)-N-methyl-2-(3,4,5-trimethoxyphenyl)acrylamide* (**12b**). The acrylic acid **1l** (1.2 eq., 189 mg, 0.526 mmol)), EDC (1.4 eq., 118 mg, 0.616 mmol) and HOBt (1.4 eq., 83 mg, 0.616 mmol) were reacted with protected cyclofenil derivative **10b** (1 eq., 200 mg, 0.44 mmol) following the general method above. The product was obtained as a brown resin. The material was purified via flash chromatography on silica gel (DCM:EtOAc, gradient 15:1 to 10:1) to afford the product as a yellow solid (272 mg, 78%), m.p. 88–90 °C. which was used immediately in the following reaction without further purification. ^1^H-NMR (CDCl_3_) δ 6.90–7.09 (m, 4H, Ar-H, C=CH), 6.52–6.85 (m, 10H, Ar-H), 4.17–4.29 (m, 1H, 0.5 × OCH_2_), 3.57–3.91 (m, 15H, 0.5 × OCH_2_, NCH_2_, 4 × OCH_3_), 3.07–3.26 (m, 3H, NCH_3_), 2.18–2.30 (m, 4H, 2 × CH_2_), 1.52–1.69 (m, 6H, 3 × CH_2_), 1.00 (s, 9H, 3 × CH_3_), 0.21 (s, 6H, 2 × CH_3_). ^13^C-NMR (CDCl_3_) δ 170.0, 153.3, 152.8, 145.9, 144.6, 130.5, 130.3, 121.5, 118.8, 115.0, 113.1, 109.7, 105.5, 66.1 (CH_2_), 60.5 (OCH_3_), 55.4 (OCH_3_), 32.0 (CH_2_), 28.2 (CH_2_), 26.4 (CH_2_), 25.2 (CH_3_), 17.7 (C(CH_3_)_3_), −4.8 (CH_3_). HRMS (EI): Found 794.4079 [M + H]^+^, C_47_H_60_NO_8_Si requires 794.4088.

*(E)-N-(2-(4-((4-((tert-Butyldimethylsilyl)oxy)phenyl)(cycloheptylidene)methyl)phenoxy)ethyl)-3-(3-hydroxy-4-methoxyphenyl)-N-methyl-2-(3,4,5-trimethoxyphenyl)acrylamide* (**12c**). The acrylic acid **1l** (1.2 eq., 185 mg, 0.516 mmol), EDC (1.4 eq., 118 mg, 0.616 mmol) and HOBt (1.4 eq., 83 mg, 0.616 mmol) were reacted with protected cyclofenil derivative **10c** (1 eq., 200 mg, 0.43 mmol) following the general method above. The product was obtained as a brown resin. The material was purified via flash chromatography on silica gel (DCM:EtOAc, gradient 15:1 to 10:1) to afford the product as a yellow solid (284 mg, 81%), m.p. 84–86 °C which was used immediately in the following reaction without further purification. ^1^H-NMR (CDCl_3_) δ 6.93–7.12 (m, 4H, Ar-H, C=CH), 6.50–6.86 (m, 10H, Ar-H), 5.64 (br. s., 1H, OH), 4.17–4.27 (m, 1H, 0.5 × OCH_2_), 3.55–3.95 (m, 15H, 0.5 × OCH_2_, NCH_2_, 4 × OCH_3_), 3.06–3.24 (m, 3H, NCH_3_), 2.24–2.37 (m, 4H, 2 × CH_2_), 1.49–1.64 (m, 8H, 4 × CH_2_), 0.99 (s, 9H, 3 × CH_3_), 0.20 (s, 6H, 2 × CH_3_). ^13^C-NMR (CDCl_3_) δ 172.1, 156.6, 153.7, 153.3, 146.4, 145.1, 139.7, 137.9, 136.8, 136.3, 135.9, 130.5, 130.3, 129.9, 128.6, 122.0, 119.4, 115.5, 113.7, 110.2, 105.8, 66.5 (CH_2_), 60.9 (OCH_3_), 56.0 (OCH_3_), 55.9 (OCH_3_), 47.7 (CH_2_), 39.0 (NCH_3_), 33.5 (CH_2_), 33.4 (CH_2_), 29.4 (CH_2_), 28.2 (CH_2_), 28.1 (CH_2_), 25.7 (CH_3_), 18.2 (C(CH_3_)_3_), −4.4 (CH_3_). IR: *ν*_max_ (KBr) cm^−1^: 3436.1 (br.). HRMS (EI): Found 830.4050 [M + H]^+^, C_48_H_61_NNaO_8_Si requires 830.4064.

*(E)-N-(2-(4-((4-((tert-Butyldimethylsilyl)oxy)phenyl)(cyclooctylidene)methyl)phenoxy)ethyl)-3-(3-hydroxy-4-methoxyphenyl)-N-methyl-2-(3,4,5-trimethoxyphenyl)acrylamide* (**12d**). The acrylic acid **1l** (1.2 eq., 180 mg, 0.5 mmol)), EDC (1.4 eq., 118 mg, 0.616 mmol) and HOBt (1.4 eq., 83 mg, 0.616 mmol) were reacted with protected cyclofenil derivative **10d** (1 eq., 200 mg, 0.416 mmol) following the general method above. The product was afforded as a brown resin. The material was purified via flash chromatography on silica gel (DCM:EtOAc, gradient 15:1 to 10:1) to afford the product as a yellow solid (243 mg, 71%), m.p. 89–91 °C which was used immediately in the following reaction without further purification. ^1^H-NMR (CDCl_3_) δ 6.95–7.15 (m, 4H, Ar-H, C=CH), 6.51–6.87 (m, 10H, Ar-H), 4.17–4.27 (m, 1H, 0.5 × OCH_2_), 3.56–3.94 (m, 15H, 0.5 × OCH_2_, NCH_2_, 4 × OCH_3_), 3.05–3.23 (m, 3H, NCH_3_), 2.20–2.34 (m, 4H, 2 × CH_2_), 1.62–1.73 (m, 2H, CH_2_), 1.48–1.61 (m, 8H, 4 × CH_2_), 0.99 (s, 9H, 3 × CH_3_), 0.20 (s, 6H, 2 × CH_3_). ^13^C-NMR (CDCl_3_) δ 171.1, 156.4, 153.1, 152.8, 145.9, 144.7, 139.5, 136.6, 135.7, 135.4, 129.7, 129.4, 128.1, 121.5, 119.1, 115.0, 113.4, 109.7, 105.5, 66.1 (CH_2_), 60.5 (OCH_3_), 60.0 (OCH_3_), 55.6 (OCH_3_), 55.4 (OCH_3_), 47.2 (CH_2_), 38.6 (NCH_3_), 32.1 (CH_2_), 32.0 (CH_2_), 26.1 (CH_2_), 26.0 (CH_2_), 25.9 (CH_2_), 25.8 (CH_2_), 25.2 (CH_3_), 17.7 (C(CH_3_)_3_), −4.9 (CH_3_). IR: *ν*_max_ (KBr) cm^−1^:3425.7 (br.), 2929.0, 2856.1, 1633.8, 1603.9, 1581.6, 1505.9, 1463.7, 1240.2, 1126.2, 838.3. HRMS (EI): Found 844.4200 [M + H]^+^, C_49_H_63_NNaO_8_Si requires 844.4221.

(E)-N-(2-(4-((4-((tert-butyldimethylsilyl)oxy)phenyl)(4-methylcyclo hexaneylidene)methyl)phenoxy)ethyl)-3-(3-hydroxy-4-methoxyphenyl)-N-methyl-2-(3,4,5-trimethoxyphenyl)acrylamide (**12e**). The acrylic acid **1l** (1.2 eq., 189 mg, 0.526 mmol), EDC (1.4 eq., 118 mg, 0.616 mmol) and HOBt (1.4 eq., 83 mg, 0.616 mmol) were reacted with protected cyclofenil derivative **10e** (1 eq., 200 mg, 0.429 mmol) following the general method above The product was afforded as a brown resin. The material was purified via flash chromatography on silica gel (DCM:EtOAc, gradient 15:1 to 10:1) to afford the product as a yellow solid (294 mg, 85%), m.p. 89–93 °C which was used immediately in the following reaction without further purification. ^1^H-NMR (CDCl_3_) δ 6.88–7.09 (m, 4H, Ar-H, C=CH), 6.53–6.86 (m, 10H, Ar-H), 5.50–5.89 (br. s., 1H, OH), 4.23 (m, 1H, 0.5 × OCH_2_), 3.57–3.92 (m, 15H, 0.5 × OCH_2_, NCH_2_, 4 × OCH_3_), 3.07–3.24 (m, 3H, NCH_3_), 2.50–2.64 (m, 2H, CH_2_), 1.87–2.00 (m, 2H, CH_2_), 1.72–1.78 (m, 2H, CH_2_), 1.05–1.14 (m, 2H, CH_2_), 1.00 (s, 9H, 3 × CH_3_), 0.91–0.97 (m, 3H, CH_3_), 0.21 (s, 6H, 2 × CH_3_). ^13^C-NMR (CDCl_3_) δ 171.7, 156.3, 153.3, 152.8, 145.9, 144.7, 137.5, 135.9, 135.4, 133.1, 130.5, 130.3, 128.1, 121.5, 118.8, 115.0, 113.1, 109.7, 105.5, 66.1 (CH_2_), 60.5 (OCH_3_), 60.0 (OCH_3_), 55.6 (OCH_3_), 55.4 (OCH_3_), 47.2 (CH_2_), 38.5 (NCH_3_) 36.4 (CH_2_), 32.3 (CH), 31.3 (CH_2_), 25.2 (CH_3_), 21.6 (CH_3_), 17.7 (C(CH_3_)_3_), −4.8 (CH_3_). IR: *ν*_max_ (KBr) cm^−1^: 3436.6 (br.), 2929.1, 2852.2, 1628.9, 1604.4, 1581.8, 1505.9, 1463.4, 1239.6, 1126.1, 839.5. HRMS (EI): Found 808.4211 [M + H]^+^, C_48_H_62_NO_8_Si requires 808.4239.

#### 3.2.9. General Method for Deprotection of *tert*-Butyldimethylsilyl Ethers **12a**–**12e**

The appropriate silyl ether **12a**–**12e** was dissolved in a minimum amount of THF (3 mL) while stirring under N_2_. An equimolar quantity of TBAF (1 M in THF) was added and the mixture was allowed to stir for 30 min. The reaction was monitored via TLC. The solvent was evaporated to dryness. The residue was redissolved in DCM (20 mL) and washed with a 10% HCl solution (10 mL). The organic phase was dried over Na_2_SO_4_ and evaporated to dryness in vacuo. The residue is purified via flash chromatography on silica gel to afford the product.

*(E)-N-(2-(4-(Cyclopentylidene(4-hydroxyphenyl)methyl)phenoxy)ethyl)-3-(3-hydroxy-4-methoxyphenyl)-N-methyl-2-(3,4,5-trimethoxyphenyl)acrylamide* (**13a**). The protected cyclofenil-acrylic acid derivative **12a** (150 mg, 0.192 mmol) and TBAF (1 M in THF) (0.192 mmol, 0.192 mL) were reacted together in THF (3 mL) following the general method above. The residue was purified via flash chromatography on silica gel (DCM:EtOAc, gradient 5:1 to 2:1) to afford the product as a yellow solid (116 mg, 91%), m.p. 159–162 °C. ^1^H-NMR (CDCl_3_) δ 6.96–7.14 (m, 4H, Ar-H, C=CH), 6.47–6.91 (m, 10H, Ar-H), 4.19–4.28 (m, 1H, 0.5 × OCH_2_), 3.56–3.95 (m, 15H, 0.5 × OCH_2_, NCH_2_, 4 × OCH_3_), 3.10–3.26 (m, 3H, NCH_3_), 2.31–2.45 (m, 4H, 2 × CH_2_), 1.62–1.74 (m, 4H, 2 × CH_2_). ^13^C-NMR (CDCl_3_) δ 171.5, 156.1, 153.7, 152.9, 145.9, 144.6, 141.7, 136.3, 135.5, 135.2, 131.3, 129.9, 128.0, 121.6, 115.0, 114.4, 113.2, 109.7, 105.5, 66.0 (CH_2_), 60.5 (OCH_3_), 55.6 (OCH_3_), 55.4 (OCH_3_), 47.4 (CH_2_), 32.8 (CH_3_), 29.3 (CH_2_), 26.5 (CH_2_). IR: *ν*_max_ (KBr) cm^−1^: 3433.5 (br.), 2933.4, 2862.0, 1603.9, 1508.1, 1408.8, 1238.4, 1125.3, 835.2. HRMS (EI): Found 688.2868 [M + Na]^+^, C_40_H_43_NNaO_8_ requires 688.2886.

*(E)-N-(2-(4-(Cyclohexaneylidene(4-hydroxyphenyl)methyl)phenoxy)ethyl)-3-(3-hydroxy-4-methoxyphenyl)-N-methyl-2-(3,4,5-trimethoxyphenyl)acrylamide* (**13b**). The protected cyclofenil-acrylic acid derivative **12b** (150 mg, 0.189 mmol) and TBAF (1 M in THF) (0.189 mmol, 0.189 ml) were reacted together in THF (3 mL) following the general method above. The residue was purified via flash chromatography on silica gel (DCM:EtOAc, gradient 5:1 to 2:1) to afford the product as a yellow solid (119 mg, 93%), m.p. 166–170 °C. ^1^H-NMR (CDCl_3_) δ 6.87–7.07 (m, 4H, Ar-H), 6.50–6.84 (m, 10H, Ar-H), 4.17–4.31 (m, 1H, 0.5 × CH_2_), 3.54–3.95 (m, 15H, 0.5 × CH_2_, CH_2_, 3 × OCH_3_), 3.08–3.24 (m, 3H, NCH_3_), 2.17–2.30 (m, 4H, 2 × CH_2_), 1.50–1.67 (m, 6H, 3 × CH_2_). ^13^C-NMR (CDCl_3_) δ 172.1, 154.0, 152.9, 146.0, 144.7, 137.8, 137.3, 135.2, 134.9, 132.9, 130.5, 130.5, 129.6, 128.0, 121.6, 115.0, 114.3, 113.2, 109.7, 105.5, 66.0 (CH_2_), 60.5 (OCH_3_), 60.0 (OCH_3_), 55.6 (OCH_3_), 55.4 (OCH_3_), 47.3 (CH_2_), 38.7 (CH_3_), 32.0 (CH_2_), 28.2 (CH_2_), 26.4 (CH_2_). IR: *ν*_max_ (KBr) cm^−1^: 3425.4 (br.), 2926.5, 2847.3, 1605.2, 1582.5, 1510.9, 1449.1, 1273.8, 1237.5, 1125.7, 1026.9, 835.4. HRMS (EI): Found 702.3026 [M + Na]^+^, C_41_H_45_NNaO_8_ requires 702.3043.

*(E)-N-(2-(4-(Cycloheptylidene(4-hydroxyphenyl)methyl)phenoxy)ethyl)-3-(3-hydroxy-4-methoxyphenyl)-N-methyl-2-(3,4,5-trimethoxyphenyl)acrylamide* (**13c**). The protected cyclofenil-acrylic acid derivative **12c** (150 mg, 0.185 mmol) and TBAF (1 M in THF) (0.185 mmol, 0.185 ml) were reacted together in THF (3 mL) following the general method above. The residue was purified via flash chromatography on silica gel (DCM:EtOAc, gradient 5:1 to 2:1) to afford the product as a yellow solid (113 mg, 98%), m.p. 162–163 °C. ^1^H-NMR (CDCl_3_) δ 6.92–7.12 (m, 4H, Ar-H, C=CH), 6.49–6.84 (m, 10H, Ar-H), 4.16–4.26 (m, 1H, 0.5 × OCH_2_), 3.53–3.93 (m, 15H, 0.5 × OCH_2_, NCH_2_, 4 × OCH_3_), 3.0. ^13^C-NMR (CDCl_3_) δ 172.2, 156.6, 154.3, 153.3, 146.4, 145.1, 139.6, 137.8, 136.9, 136.2, 135.8, 135.6, 130.5, 130.4, 130.1, 128.5, 122.0, 115.5, 114.9, 113.7, 110.2, 106.1, 66.4 (CH_2_), 60.9 (OCH_3_), 60.5 (CH_2_), 56.0 (OCH_3_), 55.9 (OCH_3_), 53.5 (CH_2_), 50.1 (CH_2_), 47.8 (CH_2_), 39.1 (NCH_3_), 33.4 (CH_2_), 29.4 (CH_2_), 28.1 (CH_2_). IR: *ν*_max_ (KBr) cm^−1^: 3437.4 (br.), 2923.4, 2842.2, 160.3, 1582.3, 1506.0, 1410.0, 1273.7, 1238.2, 1125.3, 831.2. HRMS (EI): Found 716.3185 [M + Na]^+^, C_42_H_47_NNaO_8_ requires 716.3199.

*(E)-N-(2-(4-(Cyclooctylidene(4-hydroxyphenyl)methyl)phenoxy)ethyl)-3-(3-hydroxy-4-methoxyphenyl)-N-methyl-2-(3,4,5-trimethoxyphenyl)acrylamide* (**13d**). The protected cyclofenil-acrylic acid derivative **12e** (150 mg, 0.182 mmol) and TBAF (1 M in THF) (0.182 mmol, 0.182 ml) were reacted together in THF (3 mL) following the general method above. The residue was purified via flash chromatography on silica gel (DCM:EtOAc, gradient 5:1 to 2:1) to afford the product as a yellow solid (102 mg, 79%), m.p. 158–159 °C. ^1^H-NMR (CDCl_3_) δ 6.92–7.13 (m, 4H, Ar-H, C=CH), 6.50–6.86 (m, 10H, Ar-H), 4.16–4.25 (m, 1H, 0.5 × OCH_2_), 3.51–3.95 (m, 15H, 0.5 × OCH_2_, NCH_2_, 4 × OCH_3_), 3.05–3.24 (m, 3H, NCH_3_), 2.17–2.36 (m, 4H, 2 × CH_2_), 1.62–1.74 (m, 2H, CH_2_), 1.45–1.61 (m, 8H, 4 × CH_2_). ^13^C-NMR (CDCl_3_) δ 172.4, 156.6, 153.8, 152.9, 146.0, 144.7, 139.4, 139.4, 137.3, 135.6, 135.2, 133.9, 129.6, 129.6, 121.6, 115.0, 114.6, 113.4, 109.8, 105.5, 65.9 (CH_2_), 60.5 (OCH_3_), 60.1 (CH_2_), 55.5 (OCH_3_), 55.4 (OCH_3_), 39.1 (NCH_3_), 32.0 (CH_2_), 26.1 (CH_2_), 25.9 (CH_2_), 25.8 (CH_2_). IR: *ν*_max_ (KBr) cm^−1^: 3437.6 (br.), 2927.9, 2847.3, 1604.1, 1582.3, 1505.9, 1274.6, 1238.3, 1125.6, 831.6. HRMS (EI): Found 730.3336 [M + Na]^+^, C_43_H_49_NNaO_8_ requires 730.3356.

*(E)-3-(3-Hydroxy-4-methoxyphenyl)-N-(2-(4-((4-hydroxyphenyl)(4-methylcyclohexaneylidene)methyl)-phenoxy)ethyl)-N-methyl-2-(3,4,5-trimethoxy phenyl) acrylamide* (**13e**). The protected cyclofenil-acrylic acid derivative **12e** (150 mg, 0.186 mmol) and TBAF (1 M in THF) (0.186 mmol, 0.186 ml) were reacted together in THF (3 mL) following the general method above. The residue was purified via flash chromatography on silica gel (DCM:EtOAc; gradient 5:1 to 2:1) to afford the product as a yellow solid (110 mg, 86%), m.p. 158–161 °C. ^1^H-NMR (CDCl_3_) δ 6.87–7.06 (m, 4H, Ar-H), 6.51–6.84 (m, 10H, Ar-H), 4.17–4.27 (m, 1H, 0.5 × CH_2_), 3.54–3.94 (m, 15H, 0.5 × OCH_2_, NCH_2_, 4 × OCH_3_), 3.08–3.25 (m, 3H, NCH_3_), 2.49–2.64 (m, 2H, CH_2_), 1.93 (t, *J* = 11.54 Hz, 2H, CH_2_), 1.76 (d, *J* = 10.54 Hz, 2H, CH_2_), 1.55–1.68 (m, 1H, CH), 1.01–1.15 (m, 2H, CH_2_), 0.87–0.98 (m, 3H, CH_3_). **^1^**^3^C-NMR (CDCl_3_) δ 172.2, 154.1, 152.8, 146.0, 144.7, 137.4, 134.8, 133.1, 130.5, 130.5, 128.0, 121.6, 115.0, 114.3, 113.2, 109.8, 105.5, 65.9 (CH_2_), 60.5 (OCH_3_), 60.1 (OCH_3_), 55.5 (OCH_3_), 55.4 (OCH_3_), 47.4 (CH_3_), 38.7 (NCH_3_), 36.4 (CH_2_), 32.3 (CH), 31.3 (CH_2_), 21.6 (CH_3_). IR: *ν*_max_ (KBr) cm^−1^: 3421.5 (br.), 2912.0, 2840.1, 1605.5, 1582.3, 1508.4, 1454.1, 1274.2, 1237.9, 1125.8, 830.8. HRMS (EI): Found 694.3359 [M + H]^+^, C_42_H_48_NO_8_ requires 694.3380.

*N-{2-[4-(1,2-Diphenylbut-1-enyl)phenoxy]ethyl}-N-methylsuccinamic acid* (**14a**). (i) The amine **2b** (0.10 g, 0.28 mmol) and succinic anhydride (0.03 g, 0.28 mmol) were dissolved in dry DCM (5 mL). The reaction was allowed stir at room temperature for 16 h. (The reaction was monitored via TLC, DCM:MeOH, 4:1). The reaction mixture was diluted with DCM (10 mL), washed with 1 M NaOH solution (10 mL). The aqueous phase was extracted with DCM (10 mL × 3). The combined organic layers were acidified with dilute HCl dropwise, washed with water (10 mL) and brine (10 mL), dried over sodium sulfate and then evaporated to dryness *in* vacuo to afford the product **14a** (0.13 g, 97%) as a light brown oil. (ii) Succinic acid (0.05 g, 0.42 mmol), dicyclohexylcarbodiimide (DCC) (0.09 g, 0.42 mmol) and 1-hydroxy benzotriazole hydrate (HOBt) were dissolved in dry DCM (5 mL) under a N_2_ environment. The mixture was allowed to stir for 20 minutes before adding a solution of **1b** (0.15 g, 0.42 mmol) in dry DCM (3 mL). The reaction was allowed stir at room temperature for 24 h until no starting material was visible by TLC (DCM:MeOH = 4:1). The reaction mixture was filtered to remove the dicyclohexylurea and then evaporated in vacuo and the residue was purified via flash chromatography on silica gel (DCM:MeOH = 20:1) to afford an isomeric mixture (*E:Z* = 1:1) of the product as a light brown oil (0.17 g, 90% yield). ^1^H-NMR (CDCl_3_): δ 0.95–1.00 (m, 6H, CH_3_), 2.47–2.57 (m, 4H, CH_2_), 2.61–2.86 (m, 8H, succinic CH_2_), 2.99–3.21 (m, 6H, NCH_3_), 3.65–4.18 (m, 8H, CH_2_N, CH_2_O), 6.54–7.40 (m, 28 H, ArH), 8.72 (s, 2H, COOH). ^13^C-NMR (CDCl_3_): δ 13.65, 28.08, 28.46, 28.50, 29.06, 29.58, 29.88, 34.19, 34.26, 37.48, 37.55, 48.29, 48.33, 49.17, 49.26, 64.71, 65.04, 66.25, 66.59, 113.22, 113.97, 114.00, 125.71, 125.75, 126.09, 126.14, 126.59, 127.34, 127.81, 127.91, 127.95, 128.15, 129.47, 129.71, 130.68, 130.77, 131.98, 132.06, 135.86, 136.32, 136.37, 136.83, 138.14, 138.29, 141.50, 141.69, 142.02, 142.14, 142.26, 142.32, 142.42, 143.21, 143.29, 143.75, 155.98, 156.36, 156.86, 157.21, 172.32, 172.69, 176.78, 176.85. IR: ν_max_ (KBr) cm^−1^: 3054.8, 2969.2, 1732.2, 1606.5, 1507.4, 1463.1, 1442.2, 1407.6, 1283.3, 1241.3, 1174.0, 1138.9, 1074.1, 1054.4, 1030.3, 909.8, 732.1. HRMS (EI): Found 480.2162 [M + Na]^+^, C_29_H_31_NO_4_Na requires 480.2151.

*N-[2-(4-{1-[4-(tert-Butyldimethylsilanyloxy)phenyl]-2-phenylbut-1-enyl}phenoxy)ethyl]-N-methyl-succinamic acid* (**14b**). The amine **2b** (0.20 g, 0.41 mmol, *E*/*Z* = 1:1) and succinic anhydride (0.04 g, 0.41 mmol) were dissolved in dry DCM (5mL). The reaction was allowed stir at room temperature for 16 h and monitored via TLC (DCM:MeOH = 4:1). The reaction mixture was diluted with DCM (10 mL) and washed with 1M NaOH solution (10 mL). The aqueous phase was extracted with DCM (10 mL × 3). The combined organic layers were acidified with dilute HCl dropwise, washed with water (10 mL) and brine (10 mL), dried over sodium sulfate and then evaporated to dryness in vacuo to afford an isomeric mixture (*E:Z* = 1:1) of the product (222 mg, 92%) as a light brown oil. ^1^H-NMR (CDCl_3_): δ 0.12–0.25 (m, 12H, Si(CH_3_)_2_), 0.94–1.02 (m, 24H, SiC(CH_3_)_3_), 2.48–2.52 (m, 4H, CH_2_), 2.65–2.87 (m, 8H, 4 × CH_2_), 3.00–3.22 (m, 6H, NCH_3_), 3.70–4.17 (m, 8H, CH_2_N, CH_2_O), 6.49–7.20 (m, 26H, ArH). ^13^C-NMR (CDCl_3_): δ −4.92, −4.80, 13.18, 13.22, 17.75, 25.22, 25.24, 28.46, 28.60, 37.04, 37.10, 47.91, 65.78, 66.12, 112.65, 113.42, 118.53, 119.10, 125.46, 127.31, 127.39, 127.43, 129.26, 130.09, 130.22, 130.30, 131.38, 131.57, 131.65, 135.73, 136.20, 136.27, 137.37, 137.44, 140.65, 142.08, 153.06, 156.68, 172.69, 176.78. IR: ν_max_ (KBr) cm^−1^: 3435.7, 2927.5, 1696.5, 1624.0, 1603.6, 1509.3, 1417.2, 1248.5, 1202.1, 1167.1, 928.4, 837.0. HRMS (EI): Found 610.2972 [M + Na]^+^, C_35_H_45_NO_5_SiNa requires 610.2965.

*N-(2-{4-[1,2-Bis-(4-hydroxyphenyl)but-1-enyl]phenoxy}ethyl)-N-methylsuccinamic acid* (**14c**). The protected acid **14b** (0.09 g, 0.15 mmol) was treated with TBAF following the general deprotection method above and afforded an isomeric mixture (*E:Z* = 1:1) of product **13c** as a brown oil (0.06 g, 82%). ^1^H-NMR (CDCl_3_): δ 0.92–0.96 (m, 6H, CH_3_), 2.47–2.70 (m, 12H, CH_2_, succinic CH_2_), 2.95–3.16 (m, 6H, NCH_3_), 3.61–3.76 (m, 4H, CH_2_N), 3.96–3.97 (m, 2H, CH_2_O), 4.11–4.12 (m, 2H, CH_2_O), 6.49–7.17 (m, 26 H, ArH). ^13^C-NMR (CDCl_3_): δ 13.48, 13.52, 28.76, 28.90, 37.34, 37.40, 48.21, 66.08, 66.42, 112.95, 113.72, 118.83, 119.40, 125.86, 127.61, 127.69, 127.73, 129.56, 130.39, 130.52, 130.60, 131.68, 131.87, 131.95, 136.03, 136.50, 136.57, 137.67, 137.74, 140.95, 142.38, 153.36, 156.98, 172.99, 177.08. IR: ν_max_ (KBr) cm^−1^: 3435.7, 2930.6, 1713.4, 1604.6, 1505.6, 1408.2, 1254.2, 1171.6, 912.1, 838.3. HRMS (EI): Found 496.2115 [M + H]^+^, C_29_H_31_NO_5_Na requires 496.2100.

*N-{2-[4-((Z)-1,2-Diphenylbut-1-enyl)phenoxy]ethyl}-N-methylsuccinamic acid 2-methoxy-5-[(Z)-2-(3,4,5-trimethoxyphenyl)vinyl]phenyl ester* (**16a**). *N*-{2-[4-(1,2-Diphenylbut-1-enyl)phenoxy]ethyl}-*N*-methyl-succinamic acid **13a** (0.11 g, 0.24 mmol), dicyclohexylcarbodiimide (0.05 g, 0.24 mmol) and 1-hydroxybenzotriazole hydrate (0.03 g, 0.24 mmol) were dissolved in dry DCM (5 mL) under nitrogen atmosphere. The mixture was stired for 20 min before adding a solution of combretastatin A-4 **15** (0.15 g, 0.42 mmol) in dry DCM (3 mL). The reaction was stirred at room temperature for 24 h until no starting material was visible by TLC (DCM:MeOH = 4:1). The reaction mixture was filtered to remove the dicyclohexylurea byproduct. The mixture was evaporated to dryness in vacuo and the material was purified via flash chromatography on silica gel (DCM:MeOH = 20:1) to afford the product as an isomeric mixture (*E:Z* = 1:1) as a light yellow oil (150 mg, 83% yield). ^1^H-NMR (CDCl_3_): δ 0.92–0.98 (m, 6H, CH_3_), 2.45–2.55 (m, 4H, CH_2_), 2.67–3.24 (m, 14H, succinic CH_2_, NCH_3_), 3.69–4.26 (m, 32H, OCH_3_, OCH_3_, NCH_3_), 6.47–7.39 (m, 42H, ArH). ^13^C-NMR (CDCl_3_): δ 13.17, 24.53, 25.18, 25.68, 28.58, 33.51, 36.95, 47.67, 48.63, 55.47, 55.47, 55.64, 60.46, 60.53, 102.86, 105.39, 106.08, 111.50, 111.92, 112.76, 113.50, 122.89, 124.82, 125.21, 125.61, 125.99, 126.52, 126.85, 127.09, 127.33, 127.43, 127.67, 128.19, 129.00, 129.24, 129.65, 130.20, 130.35, 131.49, 132.01, 135.82, 139.55, 149.78, 152.48, 152.92, 170.91, 174.44. IR: ν_max_ (KBr) cm^−1^: 3434.8, 3326.8, 2929.3, 2850.8, 1764.3, 1626.5, 1578.1, 1508.5, 1243.5, 1127.1, 701.3. HRMS (EI): Found 778.3353 [M + Na]^+^, C_47_H_49_NO_8_Na requires 778.3356.

*N-(2-{4-[(Z)-1-(4-Hydroxyphenyl)-2-phenylbut-1-enyl]phenoxy}ethyl)-N-methyl succinamic acid 2-methoxy-5-[(Z)-2-(3,4,5-trimethoxyphenyl)vinyl]phenyl ester* **16b**. *N*-[2-(4-{1-[4-(*tert*-Butyldimethylsilanyloxy)-phenyl]-2-phenylbut-1-enyl}phenoxy)ethyl]-*N*-methylsuccinamic acid (**14b**, 0.10 g, 0.17 mmol), dicyclohexylcarbodiimide (0.04 g, 0.17 mmol) and 1-hydroxybenzotriazole hydrate (0.02 g, 0.17 mmol) were dissolved in dry DCM (5 mL) under a nitrogen atmosphere. The mixture was allowed to stir for 20 minutes before adding a solution of combretastatin A-4 **14** (0.05 g, 0.17 mmol) in dry DCM (3 mL). The reaction was allowed stir at room temperature for 24 h until no starting material was visible by TLC (DCM:MeOH = 4:1). The reaction mixture was filtered to remove the dicyclohexylurea byproduct. The mixture was evaporated to dryness in vacuo. The residue was dissolved in 3 mL anhydrous THF and stirred under a nitrogen atmosphere. A quantity of 0.1 M TBAF (0.20 mL, 0.02 mmol) was added to the mixture and allowed stir for 24 h. The mixture was evaporated to dryness under reduced pressure. The residue was dissolved in DCM and washed with 10% HCl solution. The resulting organic phase was dried over sodium sulfate and evaporated to dryness under vacuum. The residue was purified via flash chromatography on silica gel (DCM:MeOH, 20:1) to yield an isomeric mixture (*E:Z* = 1:1) of the product **16b** (115 mg, 88%). ^1^H-NMR (CDCl_3_): δ 0.92 −0.96 (m, 6H, CH_3_), 2.46–2.54 (m, 4H, CH_2_), 2.61–3.20 (m, 14H, 4 × CH_2_, NCH_3_), 3.67–3.88 (m, 28H, OCH_3_, NCH_3_), 3.97 (t, 0.48 × 4H, *J* = 5.0 Hz, CH_2_O), 4.13 (t, 0.52 × 4H, *J* = 5.0 Hz, CH_2_O), 6.42–6.56 (m, 10H, ArH), 6.42–6.56 (m, 14H, ArH), 7.07–7.18 (m, 16H, ArH). ^13^C-NMR (CDCl_3_): δ 13.22, 24.46, 25.13, 27.95, 28.58, 28.82, 33.43, 36.91, 36.98, 47.73, 48.68, 51.40, 53.02, 55.47, 55.50, 60.49, 64.37, 65.91, 66.24, 105.41, 105.55, 109.91, 111.51, 112.65, 113.42, 113.91, 114.63, 120.65, 122.87, 125.41, 127.38, 128.53, 129.09, 129.28, 129.63, 130.11, 130.19, 130.28, 131.57, 132.31, 134.68, 135.79, 136.26, 136.57, 137.48, 142.21, 142.29, 144.81, 145.38, 149.74, 152.38, 152.45, 153.87, 154.84, 155.87, 156.72, 171.20, 173.27. IR: ν_max_ (KBr) cm^−1^: 3429.3, 2930.5, 2347.8, 1735.7, 1627.6, 1580.6, 1508.3, 1264.8, 1240.1, 1171.1, 1127.2, 834.6, 732.1. HRMS (EI): Found 794.3306 [M + Na]^+^, C_47_H_49_NO_9_Na requires 794.3305.

*N-{2-[4-((Z)-1,2-Diphenylbut-1-enyl)phenoxy]ethyl}-N-methylsuccinamic acid 2-methoxy-5-[(E)-2-methoxycarbonyl-2-(3,4,5-trimethoxyphenyl)vinyl]phenyl ester* (**16c**). *N*-{2-[4-(1,2-Diphenylbut-1-enyl)-phenoxy]ethyl}-*N*-methylsuccinamic acid (**14a**, 0.06 g, 0.14 mmol), dicyclohexylcarbodiimide (0.03 g, 0.14 mmol) and 1-hydroxybenzo triazole hydrate (0.017 g, 0.136 mmol) were dissolved in dry DCM (5 mL) under a N_2_ environment. The mixture was allowed to stir for 20 min before adding a solution of the combretastatin analogue **1s** (0.05 g, 0.14 mmol) in dry DCM (3 mL). The reaction was allowed stir at room temperature for 24 h until no starting material was visible by TLC (DCM:MeOH = 4:1). The reaction mixture was filtered to remove the dicyclohexylurea byproduct. The mixture was evaporated to dryness in vacuo and the material was purified via flash chromatography on silica gel (DCM:MeOH = 20:1) to afford an isomeric mixture (*E:Z* = 1:1) of the product as a light yellow oil (108 mg, 97% yield). ^1^H-NMR (CDCl_3_): δ 0.92 -0.98 (m, 6H, CH_3_), 2.44–2.55 (m, 4H, CH_2_), 2.65–3.22 (m, 14H, 4 × CH_2_, NCH_3_), 3.74–4.18 (m, 38H, OCH_3_, NCH_3_, CH_2_O), 6.45–7.38 (m, 40H, ArH). ^13^C-NMR (CDCl_3_): δ 13.16, 24.51, 25.17, 28.58, 33.50, 48.70, 51.95, 52.01, 55.45, 55.51, 55.65, 55.74, 60.48, 65.99, 66.31, 103.35, 106.00, 106.18, 109.71, 111.40, 111.71, 112.74, 113.48, 116.24, 122.66, 125.08, 125.21, 125.59, 126.30, 126.84, 127.14, 127.32, 128.99, 129.99, 130.12, 130.20, 130.26, 130.34, 130.73, 131.48, 133.08, 138.94, 141.87, 152.83, 153.19, 167.81, 170.63, 170.70. IR: ν_max_ (KBr) cm^−1^: 3430.0, 3326.7, 2929.2, 2850.5, 1765.4, 1710.4, 1626.6, 1579.5, 1509.0, 1274.4, 1243.7, 1126.3, 1025.2, 771.1, 701.4. HRMS (EI): Found 836.3388 [M + Na]^+^, C_49_H_51_NO_10_Na requires 836.3411.

#### 3.2.10. Stability Study for Compounds **16a**, **16b** and **16c**

Analytical high-performance liquid chromatography (HPLC) stability studies were performed using a Symmetry^®^ column (C_18_, 5 µm, 4.6 × 150 mm), a Waters 2487 Dual Wavelength Absorbance detector, a Waters 1525 binary HPLC pump and a Waters 717 plus Autosampler. Samples were detected at wavelength of 254 nm. All samples were analysed using acetonitrile (80%):water (20%) as the mobile phase over 10 min and a flow rate of 1 mL/min. Stock solutions are prepared by dissolving 5mg of each of the compounds **16a**, **16b** and **16c** in 10 mL of mobile phase. Phosphate buffers at the desired pH values (4, 7.4, and 9) were prepared in accordance with the British Pharmacopoeia monograph 2017. Thirty µL of stock solution was diluted with 1 mL of appropriate buffer, shaken and injected immediately. Samples were withdrawn and analysed at time intervals of *t* = 0 min, 5 min, 30 min, 60 min, 90 min, 120 min, 24 h and 48 h.

### 3.3. Biochemical Evaluation

#### 3.3.1. Cell Culture of MCF-7 Cell Line

The human breast carcinoma cell line, MCF-7, was purchased from the European Collection of Animal Cell Cultures (ECACC, Public Health England, Porton Down, UK). The cells were maintained in MCF-7 complete medium; consisting of Eagle’s Minimum Essential Medium (MEM) supplemented with 10% (*v*/*v*) Foetal Bovine Serum (FBS), 2 mM L-glutamine, 100 μg/mL penicillin/streptomycin and 1% (*v*/*v*) non-essential amino acids. Cell cultures were maintained at 37 °C under a humidified atmosphere of 5% CO_2_/95% O_2_. The MTT assay was performed according to the reported protocol. The tetrazolium salt, 3-(4,5-dimethylthiazol-2-yl)-2,5-diphenyltetrazolium bromide (MTT) is taken up only by metabolically active cells and cleaved to form a formazan dye by mitochondrial dehydrogenases [30]. Assays were repeated in three experiments performed in triplicate (unless otherwise stated) and reported results represent the mean value ± standard error mean. Graphs of percentage cell viability versus concentration of the subject compound were processed using PRISM [78].

#### 3.3.2. Alamar Blue Assay for Measurement of Antiproliferative Effects

The biochemical assay was performed in triplicate on at least three independent occasions for the determinationof the mean values reported. MCF-7 cells were seeded in triplicate in 96-well plates at a density of 2.5 × 10^4^ cells/mL in a total volume of 200 µL per well, and incubated at 37 °C for 24 h. Cells were treated with varying concentrations of the appropriate compounds to yield the desired final concentration. Ethanol was used as a vehicle and cells were treated with 1% ethanol (*v/v*) in all experiments. Plates were incubated for 72 h at 37 °C + 5% CO_2_ after which Alamar Blue (Invitrogen, Carlsbad, CA, USA) was added to each well. The plates were incubated for a further 4 h without exposure to light. EMEM medium with the addition of Alamar Blue was used as a blank. Vehicle treated cells were considered to be 100% viable and the viabilities of each compound was calculated accordingly. Results were calculated using transformed data [Final Concentration = Log (Final Concentration)] to plot a non-linear, sigmoidal dose response curve from which the relative IC_50_ values were determined.

#### 3.3.3. Lactate Dehydrogenase Assay for Measurement of Cytotoxicity

In this assay, the release of cytoplasmic lactate dehydrogenase (LDH) is used as a measure of cell lysis. MCF-7 cells were seeded at a density of 1 × 10^4^ cells/well in a 96-well plate and incubated for 24 h. The cells were then dosed with 2 μL volumes of the test compounds, over the concentration range 1 nM–50 μM. Cytotoxicity was determined using the CytoTox 96 nonradioactive cytotoxicity assay (Promega, Madison, WI, USA) following the manufacturer’s protocol [79].

#### 3.3.4. Estrogen Receptor Fluorescent Polarisation Assay

Competitive binding affinity experiments were carried out using purified baculovirus-expressed human ERα and ERβ and fluoromone, a fluorescein labelled estrogen ligand. ER binding ability of the selected compounds was investigated using ERα and ERβ fluorescence based ER competitive assay kits supplied by Invitrogen [67,68]. The assay was performed using a protocol described by the manufacturer. The assay allows for high throughput screening of potential ER-subtype ligands. The selected compounds were screened using both the ERα and ERβ competitive assay kits. The protocol for carrying out the assay was similar for both ER subtypes. Principally, the main difference between the kits relates to the functional receptor concentration and the specific activity of the different ERs [33,34]. The recombinant ER and the fluorescent estrogen ligand were removed from the −80° freezer and thawed on ice (4 °C) for 1 h prior to use. Previously prepared serial dilutions of the test compounds, consisting of the concentration range: 0.1 mM, 10 μM, 1 μM, 100 nM and 10 nM, were pipetted (1 μL) into a 96-well Greiner black-bottomed multiwell plate. The final concentration in the well was diluted by two orders of magnitude. The concentration range of test compounds can be adjusted accordingly to best suit the assay. Each compound was repeated in duplicate. The ES2 screening buffer (100 mM potassium phosphate (pH 7.4), 100 μg/mL bovine gamma-globulin and 0.02% sodium azide) was pipetted (49 μL) into each well containing the test compounds. The amount of ER/Fluormone^™^ ES2 complex was calculated based on a final reaction volume of 100 μL per well, thus 50 μL of complex was required per well. The complex was made up with ES2 screening buffer and was then pipetted (50 μL) to the required wells. The controls used in the assay consisted of a well containing 100 μL of screening buffer (no fluorescence expected), 50:50 complex/buffer (maximum polarisation), 1:49:50 ethanol(test sample diluent)/complex/buffer (~maximum polarisation = negative control) and 1:49:50 Estradiol(10 μM)/complex/buffer (~minimum polarisation = positive control). The plate was read on a BMG Pherastar fluorescence polarisation instrument with 485 nm excitation and 530 nm emission interference filters and processed using the Pherastar software. The plate was read over a 20 min period with polarisation readings taken every two minutes. The average of the polarisation readings was reported. Curve-fitting of the polarisation results were carried out using PRISM. The concentration of the test compound that results in a half-maximum shift in polarisation equals the IC_50_ of the test compound. The IC_50_ value is a measure of the relative binding affinity of the test compound for the ER.

#### 3.3.5. Flow Cytometry

MCF-7 cells were seeded at a density of 5 × 10^4^ cells/well in a 12-well plate and treated with the indicated compounds for 72 h. Samples were then centrifuged at 650× *g* for 5 min and resuspended in 100 µL ice-cold PBS. Ice-cold 70% (*v*/*v*) ethanol (1 mL) was then added to fix the samples overnight at 4 °C. Samples were subsequently centrifuged at 800× *g* for 10 min and resuspended in 200 µL PBS. RNase A (12.5 µL of 10 mg/mL) and propidium iodide (37.5 µL of 1 mg/ mL) were added to samples which were then incubated for 30 min at 37°C. Cell cycle analysis was performed at 488 nm using a FACS Calibur flow cytometer (Becton Dickinson, Franklin Lakes, NJ, USA). The Macintosh-based application CellQuest was then used to analyse the data of 10,000 gated cells once cell debris had been excluded. Data points represent the mean ± S.E.M. of three independent experiments.

#### 3.3.6. Generation of Human Peripheral Blood Mononuclear Cells

Fresh peripheral blood was collected from healthy donors and diluted 1:3 with RPMI medium and added to half the equivalent volume of Lymphoprep. Samples were centrifuged at 750× *g* for 30 min to form a Ficoll gradient. The white buffy layer containing the peripheral blood mononuclear cells was removed, diluted to a volume of 50 mL with medium and centrifuged again for 10 min at 650× *g*. Cells were then seeded at a density of 1 × 10^6^ cells in 1 mL of RPMI medium supplemented with 10% FBS, 2 mM l-glutamine and 100 U/mL penicillin and 100 µg/mL streptomycin.

#### 3.3.7. NCI One-Dose and Five-Dose Screen Output

The NCI one-dose screen output is reported as a mean graph of the percent growth of treated cells and is similar in appearance to mean graphs generated in the 5-dose assay [80]. The value reported for the one-dose assay is growth relative to the no-drug control and relative to the time zero number of cells. The one-dose assay allows detection of growth inhibition (values between 0 and 100) and lethality (values less than 0). For example, a value of 100 means no growth inhibition. A value of 40 would mean 60% growth inhibition. A value of zero means no net growth over the course of the experiment. A value of −40 would mean 40% lethality. A value of −100 means all cells are dead. The results from the five-dose screen for **15a** were manually entered into the COMPARE analysis software via an on-line submission form [80]. The results from the COMPARE analysis are retrievable on-line by searching using the relevant JobID reference number. The COMPARE analysis was run on a database of common anti-cancer agents (JobID: 37888) and the larger more comprehensive database including natural products and other submitted agents (JobID: 37889).

### 3.4. Computational Details: Molecular Docking Study

The 3ERT X-ray structure of hERα cocrystallized with 4-OHT was downloaded from the PDB Web site [69,81]. For ERβ, the 1NDE X-ray structure cocrystallized with the triazine ERβ modulator 4-(2-{[4-{[3-(4-chlorophenyl)-propyl]sulfanyl}-6-(1-piperazinyl)-1,3,5-triazin-2-yl]amino}ethyl)-phenol was downloaded [70]. Both were prepared using QuickPrep in MOE 2016.0802 [82]. Water molecules in proximity to the ligands were retained. The preprocessed X-ray structures were previously validated by our group [83]. Compounds were drawn in ChemBioDraw Ultra 13.0.2.3021 (PerkinElmer) and converted to sd files within Pipeline Pilot [BIOVIA, Dassault Systèmes, http://accelrys.com/products/collaborative-science/biovia-pipeline-pilot/]. MOE Dock was utilised for generation of the three compounds docking poses using default parameters with the exception of placing and refining 100 poses [82]. Over 300 conformers of each compound were generated stochastically in MOE for docking.

## 4. Conclusions

The estrogen receptors ERα and ERβ which modulate the effects of the estrogen hormones are important targets for design of chemotherapeutic agents whch target diseases such as breast cancer and osteoporosis. A series of novel conjugate molecules incorporating the ER ligands endoxifen and cyclofenil-endoxifen hybrids covalently linked to the antimitotic and tubulin targetting agent CA-4 were synthesised. These conjugates were evaluated as ER targeting ligands. A number of these compounds demonstrated pro-apoptotic effects, with significant antiproliferative activity in ER-positive MCF-7 breast cancer cell lines. These conjugates displayed potent binding affinity towards ERα and ERβ isoforms at nanomolar concentrations as determined by FP assay. The endoxifen conjugate **16b** demonstrates antiproliferative activity in ER positive MCF-7 breast cancer cells (IC_50_ = 5.7 nM) and ER binding affinity to ERα (IC_50_ = 52 nM) and ERβ (IC_50_ = 115 nM). The conjugate **16a** displayed significant growth inhibition effects on the NCI 60 cancer cell panel, suggesting that this compound may also inhibit cancer cell growth through non-ER dependent mechanisms. These conjugate compounds are identified as suitable lead compounds for future drug development based on favourable in vitro stability profile over pH range 4–9 and also in plasma. The conjugate **16a** differs from **16b** only by a hydroxy functionality which is known to improve the binding of the ER-ligand fragment and is presumably the major factor in the 18-fold difference in antiproliferative potency. This is reinforced by the high affinity binding of **16b** to ERα with an IC_50_ ERα of 52.1 nM. It remains unclear the exact role the combretastatin moiety has in the antiproliferative effect. These two conjugates **16a** and **16b** are covalently bound to the CA4 fragment by ester linkages which could be readily hydrolysed in vivo thus releasing the CA4. The compound **14a** represents the cleaved endoxifen-linker component of conjugate **16a** and displays very low antiproliferative activity. The intact conjugate may be exerting antiproliferative activity through binding in the ER resulting in the displacement of helix-12 and ER-antagonism. However, if the conjugate is cleaved the combretastatin may be exerting its antimitotic activity. Additionally, the conjugate may be working through both an ER-antagonistic pathway and antimitotic pathway i.e., through dual-action.

The cyclofenil-amide compound **13e** is also identified as a promising lead compound of a clinically relevant ER conjugate with IC_50_ in MCF-7 cells of 187 nM, and ER binding affinity to ERα (IC_50_ = 19 nM) and ERβ (IC_50_ = 229 nM), which can target ER in the breast cancer cell and can thus deliver a cytotoxic agent CA4 to the cancer cell. Conjugate and bifunctional compounds which incorporate an ER ligand offer a useful method of delivering cytotoxic drugs to tissue sites such as breast cancers which express ERs. From the results obtained in the NCI 60 cell line assay, it is clear that compounds such as **16a**, although displaying nanomolar potency for MCF-7 ER positive cells and high affinity ER binding, do not display selectivity in activity towards the ER-expressing cell line MCF-7, indicating that **16a** may also be modulating additional biological mechanisms (i.e., in promoting apoptosis or inhibition of tubulin polymerisation) other than via the ER alone. This result highlights possible therapeutic applications for these prototype ER-conjugates. Further research may determine the mechanism of antiproliferative action of these compounds in cancer cells. These conjugate compounds have potential application for further development as antineoplastic agents, (possibly modulating more than a single molecular target–in this case tubulin), in the treatment of both ER positive and ER negative breast cancers and these chemical classes of structures may be useful as scaffolds to be considered for multitarget drugs in future drug development studies.

## Figures and Tables

**Table 1:** Structures of acrylic acids **1a**–**1r** (see Scheme 1).

Compound	R_1_	R_2_	R_3_	R_4_	R_5_	R_6_	R_7_	Yield (%)
**1a**	OMe	OMe	OMe	H	OMe	NO_2_	H	63
**1b**	OMe	H	OMe	OMe	OMe	OMe	H	35
**1c**	OMe	OMe	OMe	OMe	OMe	OMe	H	96
**1d**	OMe	H	OMe	H	OMe	OH	H	7
**1e**	OMe	OMe	OMe	H	OH	OH	H	36
**1f**	OMe	OMe	OMe	H	H	OH	H	88
**1g**	OMe	OMe	OMe	H	OMe	OMe	H	45
**1h**	OMe	OMe	OMe	H	OMe	OMe	OMe	30
**1i**	H	OMe	OH	OMe	OMe	OMe	H	38
**1j**	OMe	OMe	OMe	OMe	OH	OMe	H	48
**1k**	H	OMe	H	H	OCOCH_3_	H	H	56
**1l**	OMe	OMe	OMe	H	OMe	OH	H	36
**1m**	H	OMe	H	H	OCH_2_O		H	49
**1n**	OMe	OMe	OMe	H	OMe	H	H	71
**1o**	H	OMe	H	OMe	OMe	OMe	H	47
**1p**	OMe	OMe	OMe	-	2-Methylthienyl	-	-	45
**1q**	OMe	OMe	OMe	-	3-Furfuryl	-	-	64
**1r**	OMe	OMe	OMe	H	OMe	OH	H	83

**Table 2:**
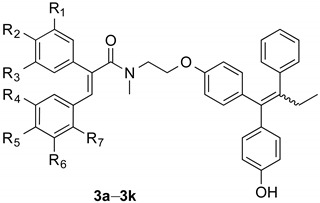
Conjugate compounds **3a**–**3k**.

Compound	R_1_	R_2_	R_3_	R_4_	R_5_	R_6_	R_7_	Yield *
**3a**	OMe	OMe	OMe	H	OMe	NO_2_	H	82 ^b^
**3b**	OMe	H	OMe	OMe	OMe	OMe	H	33 ^a^
**3c**	OMe	OMe	OMe	OMe	OMe	OMe	H	83 ^b^
**3d**	OMe	H	OMe	H	OMe	OH	H	54 ^a^
**3e**	OMe	OMe	OMe	H	OH	OH	H	60 ^b^
**3f**	OMe	OMe	OMe	H	H	OH	H	45 ^a^
**3g**	OMe	OMe	OMe	H	OMe	OMe	H	79 ^b^
**3h**	OMe	OMe	OMe	H	OMe	OMe	OMe	26 ^a^
**3i**	H	OMe	OH	OMe	OMe	OMe	H	77 ^b^
**3j**	OMe	OMe	OMe	OMe	OH	OMe	H	71 ^b^
**3k**	H	OMe	H	H	OCH_2_O		H	45 ^b^
**3l**	OMe	OMe	OMe	2-Methylthienyl	-	-	-	16 ^b^
**3m**	OMe	OMe	OMe	3-Furfuryl	-	-	-	76 ^b^

* Overall yield: coupling and deprotection. ^a^ (i) DCC, HOBt, CH_2_Cl_2_, 24 h, 20 °C; (ii)TBAF, THF, 0.5 h, 20 °C; ^b^ (i) EDC, HOBt, CH_2_Cl_2_, 24 h, 20 °C; (ii) TBAF, THF, 0.5 h, 20 °C.

**Table 3:** Antiproliferative effects of acrylic acid conjugates in MCF-7 human breast cancer cells ^a^.

Compound	IC_50_ (μM) ^b^	cLogP ^j^	Compound	IC_50_ (μM) ^b^	cLogP ^j^
**3a**	>50	8.19	**11a**	3.09	4.62
**3b**	>50	8.02	**11b**	4.78	5.18
**3c**	>50	7.61	**11c**	1.38	5.74
**3d**	>50	8.40	**11d**	3.23	6.30
**3e**	>50	7.42	**11e**	1.77	5.70
**3f**	>50	7.84	**5a**	51.3	7.04
**3g**	>50	7.62	**5b**	46.77	6.82
**3h**	>50	7.63	**5c**	>50	7.16
**3i**	4.20	7.69	**5d**	>50	6.94
**3j**	11.75	9.09	**13a**	1.38	6.75
**3k**	1.47	7.33	**13b**	2.01	7.31
**3l**	>50	8.65	**13c**	2.50	7.87
**3m**	36.5	7.68	**13d**	0.346	7.83
**3n**	^f^	2.15	**13e**	0.182	7.83
**3o**	^g^	1.75	**16a**	0.090	8.73
**3p**	^h^	2.15	**16b**	0.0057	9.21
**3q**	^i^	3.33	**14a**	>50	5.28
			Tamoxifen ^e^	2.13	6.82
			Endoxifen ^d^	0.029	6.04
			CA4 ^c^	0.008	3.32

^a^ Mean IC_50_ values of compounds for their antiproliferative effects and percent cytotoxicity on a human MCF-7 breast cancer cell line. ^b^ IC_50_ values are half maximal inhibitory concentrations required to block the growth stimulation of MCF-7 cells. Values are an average of at least three experiments performed in triplicate with typical standard errors below 15%. ^c^ The IC_50_ value obtained for **14** (CA4) in this assay is 8 nM for MCF-7 and is in good agreement with the reported values for CA4 in MCF-7 cell line [61,62,63]. ^d^ The reported IC_50_ value for endoxifen in the MCF-7 cell line is 50 nM [65]. *^e^* The IC_50_ value for Tamoxifen is in agreement with the reported IC_50_ value in MCF-7 cells (4.4 μM) [64]. ^f^ 20% growth inhibition for compound **3n** at 1 μM. ^g^ 16% growth inhibition for compound **3o** at 1 μM. ^h^ 60% growth inhibition for compound **3p** at 1 μM. ^i^ 25% growth inhibition for compound **3q** at 1 μM. ^j^ cLogP values calculated from ChemDraw Ultra, version 12.0. (PerkinElmer, Waltham, MA, USA).

**Table 4:** In vitro human cancer cell growth inhibition for compound **16a** *^a^*: Antiproliferative evaluation in the NCI60 cell line in vitro screen.

Cell Line	Compound 16a	Compound 16a	Cell Line	Compound 16a	Compound 16a
	Growth % (10^−5^ M) ^a^	GI_50_ (μM) ^b^		Growth % (10^−5^ M) ^a^	GI_50_ (μM) ^b^
*Leukemia*			*Melanoma*		
CCRF-CEM	20.38	0.0394	LOX IMV1	28.97	0.0358
HL-60(TB)	28.19	0.0145	MALME-3M	74.86	>10.00
K-562	41.7	<0.0100	M14	1.39	0.0129
MOLT-4	36.2	0.0398	MDA-MB-435	−16.61 ^e^	<0.0100
RPMI-8226	−18.51 ^e^	0.0271	SK-MEL-2	37.97	>10.00
SR	29.68	0.0189	SK-MEL-28	89.31	<0.0100
*Non-Small Cell Lung Cancer*			SK-MEL-5	−34.34 ^e^	0.0201
A549/ATCC	18.56	0.059	UACC-257	48.73	>10.00
EKVX	22.72	0.0569	UACC-62	25.13	0.0181
HOP-62	35.29	0.0492	*Ovarian cancer*		
HOP-92	58.55	<0.0100	IGROV1	−26.34 ^e^	0.0473
			OVCAR-3	−57.79 ^e^	<0.0100
NCI-H226	11.59	0.0471	OVCAR-4	30.96	0.0628
NCI-H23	12.25	0.0459	OVCAR-5	34.65	0.0894
NCI-H332M	57.18	0.0672	OVCAR-8	12.79	0.049
NCI-H460	−6.77 ^e^	0.0299	NCI/ADR-RES	−41.32 ^e^	0.0111
NCI-H552	−30.85 ^e^	0.0197	SK-OV-3	17.23	0.0368
*Colon Cancer*			*Renal cancer*		
COLO 205	−55 ^e^	0.378	786-0	11.54	0.185
HCT-2998	4.72	0.0317	A498	29.47	0.0581
HCT-116	1.61	0.0156	ACHN	22.73	0.0455
HCT-15	0.71	0.0245	CAKI-1	23.4	0.0307
HT29	6.79	0.489	RXF 393	12.08	0.0398
KM12	−37 ^e^	<0.0100	SN12C	31.84	0.0576
SW-620	28.17	<0.0100	TK-10	33.77	0.589
*CNS Cancer*			UO-31	31.52	0.0884
SF-268	18.51	0.0253	*Breast cancer*		
SF295	6.21	0.0208	MCF-7	10.06	0.0345
SF539	−7.31 ^e^	0.0213	MDA-MB-231/ATCC	23.19	0.0286
SNB-19	28.03	0.0365	HS 578T	17.57	0.0255
SNB-75	39.46	0.0166	BT-549	7.07	0.0438
U251	10.13	0.0316	T-47D	59.73	>10
*Prostate cancer*			MDA-MB-468	0.68	0.0343
PC-3	19.1	0.012	**MG-MID (μM) ^c^**	-	1.62
DU-145	−36.33 ^e^	0.0125	**MG% (10^−5^ M) ^d^**	14.74	-

^a^ Data obtained from NCI in vitro human tumour cell screen at 10 μM concentration. ^b^ GI_50_ is the molar concentration of the compound causing 50% inhibition of growth of the tumour cells. ^c^ MG-MID: the mean of GI_50_ values over all cell lines for the tested compound. ^d^ MG% (10^−5^ M): Mean Growth % over all cell lines for the tested compound. ^e^ Cytotoxic effect.

**Table 5:** Estrogen receptor binding affinities for compounds ^a^.

Compound	ERα IC_50_ (nM) ^a,b^	ERβ IC_50_ (nM) ^a,b^	ERα RBA ^c^	ERβ RBA ^c^	Selectivity (α/β)
**3i**	182	436	3.13	1.28	2.44
**11c**	1738	199	0.328	2.81	0.12
**11d**	170	112	3.35	5.00	0.67
**11e**	3162	67.0	0.180	8.35	0.02
**13e**	19.0	229	30.0	2.44	12.3
**14c**	11.3	6.7	50.44	83.6	0.60
**16a**	79.9	22.7	7.13	24.66	0.29
**16b**	52.1	115	10.94	4.87	2.25
**16c**	70.9	90.1	8.04	6.22	1.29
Endoxifen	47.0	118	12.1	4.75	2.55
4-Hydroxytamoxifen	30.0	26.1	19.0	21.5	0.88
Estradiol	5.7	5.6	-	-	0.98

^a^ Mean IC_50_ values of compounds for their competitive binding in ERα and ERβ isoforms. ^b^ Values are an average of at least nine replicate experiments, for ERα with typical standard errors below 15%, and six replicate experiments for ERβ, with typical standard errors below 15%. ^c^ The relative binding affinity (RBA) of ER ligands is reported, using IC_50_ values for estradiol as the reference ligand [ERα (5.7 nM) and ERβ (5.6 nM); 100% binding value.]

## Appendix Supplementary Material

The following are available online.
