# Supplementary material for: Novel Selective Estrogen Receptor Ligand Conjugates Incorporating Endoxifen-Combretastatin and Cyclofenil-Combretastatin Hybrid Scaffolds: Synthesis and Biochemical Evaluation
Source: Molecules. 2017 Aug 31;22(9):1440. doi: 10.3390/molecules22091440 (PMC6151695; doi:10.3390/molecules22091440)
Supplement: Supplementary file 1 [file molecules-22-01440-s001.pdf]

Supplementary Information

Synthesis and biochemical evaluation of novel selective estrogen receptor ligand conjugates incorporating endoxifen-combretastatin and combretastatin hybrid scaffolds

Patrick J. Kelly[1], Niall O. Keeley[2], Sandra A. Bright[3], Bassem Yassin[2], Gloria Ana[1], Darren Fayne[3], Daniela Zisterer[3] and Mary J Meegan[1,2]

[1] School of Pharmacy and Pharmaceutical Sciences, Trinity Biomedical Sciences Institute, 152-160 Pearse Street, Trinity College Dublin, Dublin 2, Ireland; kellyp9@tcd.ie(P.J.K.); anag@tcd.ie(G.A.)

[2] School of Pharmacy and Pharmaceutical Sciences, Trinity College Dublin, Dublin 2, Ireland; nkeely@tcd.ie (N.O.K.); bassem76@yahoo.com (B.Y.)

[3] School of Biochemistry and Immunology, Trinity Biomedical Sciences Institute,152-160 Pearse Street, Trinity College Dublin, Dublin 2, Ireland; brightsandi@tcdgmail.com(S.A.B); FAYNED@tcd.ie(DF); dzistrer@tcd.ie (D.Z.)

1. Synthesis of phenylacetic acids **K , L**

2. Synthesis of cinnamic acids **4a, 4c** and phenylpropanoic acids **4b, 4d**

Synthesis of phenylacetic acids **K** and **L**

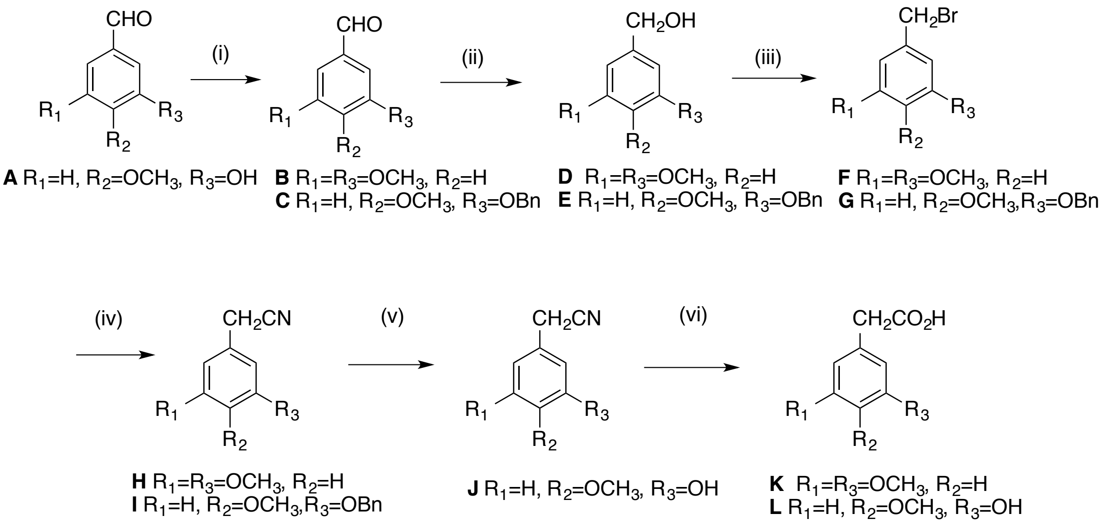

Scheme S1. Synthesis of phenylacetic acids **K** and **L**. Reagents and conditions: KOH, $C_6H_5CH_2Br$, EtOH, 6h, reflux; (ii) $NaBH_4$, THF, 3 h, 20 ºC; (iii)PBr_3, DCM, 1 h, 20 ºC; (iv) NaCN, DMF, 1h,20 ºC; (v) Pd/C, black, EtOAc, H_2, 24 h, Pressure reactor;(vi) conc HCl, 2.5 h, reflux.

Scheme S1: Synthesis of phenylacetic acids **L** and **K**

Reagents and conditions: (i) $C_6H_5CH_2Br$, KOH, EtOH, 6 h, reflux; (ii) $NaBH_4$, THF, 3 h, RT; (iii) PBr_3, DCM, 1 h, RT; (iv) NaCN, DMF, 1 h, RT; (v) Pd/C black, EtOH, H_2, 24 h, Pressure reactor; (vi) Conc HCl, 2.5 h, reflux.

3-(Benzyloxy)-4-methoxybenzaldehyde **C**

To a solution of KOH (1.3 g, 20 mmol) in ethanol (12 mL) was added 3-hydroxy-4-methoxybenzaldehyde **A** (2 g, 10 mmol) and benzyl bromide (4.4 g, 10 mmol). The solution was heated under reflux for 6 h and then evaporated to dryness *in vacuo*. The residue was purified by flash chromatography on silica gel (*n*-Hexane:EtOAc, 4:1) to afford the product as a cream solid. (1.3 g, 53 %), Mp. 61-64 ºC. ^{1}H-NMR 400MHz ($CDCl_3$): 9.834 (s, 1H, CHO), 7.496-7.285 (m, 7H, Ar-H), 7.01 (d, 1H, J=8Hz, Ar-H), 5.20 (s, 2H, CH_2), 3.978 (s, 3H, OCH_3). ^{13}C-NMR 100MHz ($CDCl_3$): 190.58 (CHO), 155.05, 148.70, 136.30, 129.98, 128.65, 128.15, 127.51, 126.95, 111.30, 110.78, 70.83 (CH_2), 56.21 (OCH_3). IR: v_{max} (KBr) cm^{-1}: 2910.72, 1702.38, 1606.56, 1207.33, 1150.42. HRMS (EI): Found 265.0831 $(M+Na)^+$, $C_{15}H_{14}O_3Na$ requires 265.0841.

(3,5-Dimethoxyphenyl)methanol **D**

$LiAlH_4$ (2.33 g, 61.42 mmol) was added to a solution of 3,5-dimethoxybenzaldehyde **B** (5.6 g, 30.7 mmol) in THF (100 mL) and the mixture was stirred for 3 h. Acetic acid (1 mL) was added, followed by $NaHCO_3$ (10 g) in water (50 mL) to neutralize the residual aqueous extract. The resulting mixture was filtered through celite and extracted with ethyl acetate (100 mL × 3), brine (100 mL), dried over sodium sulphate and evaporated *in vacuo* to yield the product as a white solid, (5.05 g, 98 %), Mp. 63-65 °C, which did not require further purification. ^{1}H-NMR 400MHz ($CDCl_3$):6.52 (s, 2H, Ar-H), 6.38 (s, 1H, Ar-H), 4.61 (s, 2H, CH_2), 3.79 (s, 6H, 2 × OCH_3) ^{13}C-NMR 100MHz ($CDCl_3$): 160.46, 142.98, 104.08, 99.11, 64.76 (CH_2OH), 54.88 (O CH_3) IR: ν_{max} (KBr) cm^{-1}: 3343.1, 2933.4, 1678.0, 1598.1, 1504.3, 1456.1, 1383.3, 1005.6, 806.9, 697.1, 640.5 HRMS (EI): Found 169.0775 $(M+H)^+$, $C_9H_{13}O_3$ requires 169.0865.

(3-(Benzyloxy)-4-methoxyphenyl)methanol **E**

$NaBH_4$ (0.5 g, 13.2 mmol) was added to a solution of 4-benzyloxy-3-methoxybenzaldehyde **C** (3 g, 12.3 mmol) in 50 mL of methanol. After stirring for 3 h, acetic acid was added (1 mL). To this $NaHCO_3$ (10 g) in 50 mL of water and the solution extractred with ethyl acetate (50 mL × 3), brine (50 mL), dried over sodium sulphate and evaporated to dryness *in vacuo* to yield product as a white solid, (2.86 g, 95 %), Mp. 65-66 °C. The material did not require further purification. ^{1}H-NMR 400MHz ($CDCl_3$): 7.48-7.28 (m, 5H, Ar-H), 7.05-6.87 (m, 3H, Ar-H), 5.18 (s, 2H, CH_2), 4.59(s, 2H, CH_2), 3.90 (s, 3H, CH_3) ^{13}C-NMR 100MHz ($CDCl_3$): 149.26, 148.29, 137.06, 135.35, 128.57, 127.88, 120.04, 113.11, 111.70, 70.95 (CH_2), 65.23 (CH_2OH), 56.11(OCH_3). IR: ν_{max} (KBr) cm^{-1}: 3204.17, 1591.6, 1425.3, 1240.7, 1157.8, 1008.6, 754.0. HRMS (EI): Found 267.1007 $(M+Na)^+$, $C_{15}H_{16}O_3Na$ requires 267.0997.

1-(Bromomethyl)-3,5-dimethoxybenzene **F**

A solution of the benzylic alcohol **D** (1.8 g, 10.62mmol), in DCM (30 mL), was cooled to 0 ^{o}C at which time PBr_3 (1 M in DCM) (1 mL, 10.62 mmol) was added. After 1 h at room temperature, saturated $NaHCO_3$ was added dropwise and the aqueous layer was extracted with dichloromethane (50 mL × 3), ethyl acetate (50 mL × 3), brine (50 mL), dried over sodium sulphate and evaporated to dryness to yield product as a

white solid, (1.97 g, 80 %), Mp. 67-70 °C. The material did not require further purification, ^{1}H-NMR 400MHz ($CDCl_3$): 6.56 (d, 2H, *J*= 2 Hz, Ar-H), 6.41 (t, 1H, *J*= 2 Hz, Ar-H), 4.42 (s, 2H, CH_2), 3.82 (s, 6H, OCH_3) ^{13}C-NMR 100MHz ($CDCl_3$): 33.2 (CH_2), 54.9 (O CH_3), 100.13, 106.49, 139.27, 160.44 IR: $ν_{max}$ (KBr) cm^{-1}: 3003.4, 2969.9, 2936.5, 1609.4, 1517.8, 1162.3, 1006.8, 811.6, 696.4. HRMS (EI): Found 230.9942 $(M+H)^+$, $C_9H_{12}BrO_2$ requires 230.9942.

1-((5-(Bromomethyl)-2-methoxyphenoxy)methyl)benzene **G**

A solution of the benzylic alcohol **E** (4.692 g, 19.09 mmol), in dichloromethane (30 mL), was cooled to 0 ^{0}C at which time PBr_3 (1 M in DCM) (1.8 mL, 19.09 mmol) was added. After 1 h at room temperature saturated $NaHCO_3$ was added dropwise and the aqueous layer was extracted with dichloromethane (50 mL × 3). It was extraxtred with ethyl acetate (50mL × 3), brine (50 mL), dried over sodium sulphate and evaporated to dryness *in vacuo* to yield product as a white solid, (5.46 g, 93 %), Mp. 77-78 °C. The material did not require further purification. ^{1}H-NMR 400MHz ($CDCl_3$): 7.38-7.48 (m, 5H, Ar-H), 6.85-7.00 (m, 3H, Ar-H), 5.18 (s, 2H, CH_2), 4.48 (s, 2H, CH_2), 3.91 (s, 3H, OCH_3). ^{13}C-NMR 100MHz ($CDCl_3$): 149.94, 148.25, 136.79, 130.15, 128.82, 128.60, 127.98, 127.44, 122.16, 114.78, 111.59, 77.06 (CH_2), 56.05 (OCH_3), 34.36 (CH_2Br). IR: $ν_{max}$ (KBr) cm^{-1}: 3444.0, 3033.4, 3969,1609.4, 1517.8, 1162.3, 1006.8, 811.6, 696.4. HRMS (EI): Found 307.0255 $(M+H)^+$, $C_{15}H_{16}BrO_2$ requires 307.0334.

2-(3,5-Dimethoxyphenyl)acetonitrile **H**

To a solution of the benzylic bromide **F** (1.5 g, 6.49 mmol) in DMF (25 mL), was added sodium cyanide (0.33 g, 6.73 mmol). After 1h, water was added and the aqueous layer was extracted with ethyl acetate (50 mL × 3), brine (50 mL), dried over sodium sulphate and evaporated to dryness to yield product as a white solid, (1.1 g, 74 %), Mp. 74-78 °C. The material did not require further purification. ^{1}H-NMR 400MHz ($CDCl_3$): 6.48 (s, 2H, Ar-H), 6.42 (s, 1H, Ar-H), 3.81 (s, 6H, O CH_3), 3.70 (s, 2H, CH_2) ^{13}C-NMR 100MHz ($CDCl_3$): 160.83, 131.50, 117.32 (CN), 105.56, 99.41, 54.98 (O CH_3), 54.94 (OCH_3), 23.30 CH_2) IR: $ν_{max}$ (KBr) cm^{-1}: 2941.6, 2246.4, 1590.4, 1515.4,

1289.7, 1238.2, 1141.8, 1009.2. HRMS (EI): Found 178.0780 $(M+H)^+$, $C_{10}H_{12}NO_2$ requires 178.0868.

2-(3-(Benzyloxy)-4-methoxyphenyl)acetonitrile **I**

To a solution of the benzylic bromide **G** (5.46 g, 17.78 mmol) in DMF (40 mL), was added sodium cyanide (1.127 g, 23 mmol). After 1 h water was added and the aqueous layer. It was extracted with ethyl acetate (50 mL × 3), brine (50 mL), dried over sodium sulphate and evaporated to dryness in vacuo to yield product white solid, (3.95 g, 87 %), Mp. 77-78 °C. The material did not require further purification. ^{1}H-NMR 400MHz (DMSO-d_6): 7.47 (d, 2H, J=7Hz, Ar-H), 7.41 (t, 2H, J=7Hz, Ar-H), 7.36 (d, 1H, 7Hz, Ar-H), 7.06 (s, 1H, Ar-H), 7.00 (m, 1H, Ar-H), 6.90 (m, 1H, Ar-H), 5.07 (s, 2H, CH_2), 3.92 (s, 2H, CH_2), 3.77 (s, 3H, OCH_3). ^{13}C-NMR 100MHz (DMSO-d_6): 149.52, 148.53, 136.67, 128.65, 128.05, 127.40, 122.04, 120.80, 118.12, 113.70, 112.08, 71.13 (CH_2), 56.10 (OCH_3), 23.16 (CH_2NC). IR: ν_{max} (KBr) cm^{-1}: 2936.6, 2246.4, 1590.4, 1515.4, 1289.7, 1238.2, 1141.8, 1009.1, 794.7, 705.4. HRMS (EI): Found 276.1005 $(M+Na)^+$, $C_{16}H_{15}NO_2Na$ requires 276.1000.

2-(3-Hydroxy-4-methoxyphenyl)acetonitrile **J**

The benzylic cyanide **I** (3 g, 11.84 mmol) was dissolved in a 50/50 mixture of methanol and ethyl acetate (20 mL). To this solution Pd/C Black (80 mg) was added and hydrogenated in a Parr hydrogenator under 3 bar pressure for 24 h. The resulting mixture was filtered through celite. The solution was evaporated to dryness, water (20 mL) was added; the aqueous phase was extracted with ethyl acetate (20 mL × 3), brine (50 mL), dried over sodium sulphate and evaporated to dryness *in vacuo*. The material was purified *via* flash chromatography on silica gel (*n*-Hexane:EtOAc, 1:1) to afford the product as a white solid, (1.5 g, 77 %), Mp. 106-107 °C. ^{1}H-NMR 400MHz ($CDCl_3$): 6.89 (s, 1H, Ar-H), 6.84 (s, 2H, Ar-H), 3.91(s, 3H, O CH_3), 3.67 (s, 2H, CH_2). ^{13}C-NMR 100MHz ($CDCl_3$): 146.03 ($\underline{C}OCH_3$), 145.60 ($\underline{C}OH$), 122.36, 119.11, 117.88 (CN), 113.91, 110.72, 55.60 (OCH_3), 22.44 (CH_2CN). IR: ν_{max} (KBr) cm^{-1}: 3416.3, 2936.5, 2249.9, 1717.8, 1592.8, 1515.1, 1263.6, 1140.1, 1023.5, 698.1. HRMS (MS Cl+): Found $C_9H_9NO_2$ $(M+H)^+$, 164.0619 requires 164.0633.

2-(3,5-Dimethoxyphenyl)acetic acid **K**

A concentrated solution of hydrochloric acid (50 mL) was added to the benzylic cyanide **H** (3.135 g, 17.6 mmol) and heated at reflux for 2.5 h. Water (50 mL) was added and the reaction mixture was allowed to cool to room temperature. The aqueous phase was extracted with dichloromethane (50 mL × 3), brine (50 mL), dried over sodium sulphate and evaporated to dryness *in vacuo.* The crude product was purified *via* flash chromatography on silica gel (*n*-Hexane:EtOAc, 1:1) to afford the product as a white solid. (2.14 g, 61 %), Mp. 101-103 °C. ^{1}H-NMR 400MHz ($CDCl_3$): 6.46 (s, 2H, Ar-H), 6.41 (s, 1H, Ar-H), 3.81 (s, 6H, O CH_3), 3.61 (s, 2H, CH_2). ^{13}C-NMR 100MHz ($CDCl_3$): 177.68 (COOH), 160.89, 135.27, 107.48, 99.43, 55.35 (OCH_3), 41.30 (CH_2). IR: ν_{max} (KBr) cm^{-1}: 2910.72, 1702.38, 1606.56, 1207.33, 1150.42. HRMS (EI): Found 197.0711 $(M+H)^+$, $C_{10}H_{13}O_4$ requires 197.0814.

2-(3-Hydroxy-4-methoxyphenyl)acetic acid **L**

A concentrated solution of hydrochloric acid (100 mL) was added to the deprotected benzylic cyanide **J** (3 g, 18.38 mmol) and the mixture heated at reflux for 2.5 h. Water (50 mL) was added and the reaction mixture was allowed to cool to room temperature. The aqueous phase was extracted with dichloromethane (50 mL × 3), brine (50 mL), dried over sodium sulphate and evaporated to dryness *in vacuo* to yield product. The material was purified *via* flash chromatography on silica gel (*n*-Hexane:EtOAc, 1:1) to afford the product as a white solid, (700 mg, 21 %), Mp. 130-131 °C. ^{1}H-NMR 400MHz ($CDCl_3$): 6.86 (d, 1H, 8.5Hz, Ar-H), 6.78 (d, 1H, 2Hz, Ar-H), 6.73 (dd, 1H, 8.5Hz, 2Hz, Ar-H), 3.84 (s, 3H, O CH_3), 3.47 (s, 2H, CH_2). ^{13}C-NMR 100MHz ($CDCl_3$): 174.90 (COOH), 147.10 (CO CH_3), 146.51 (COH), 127.82, 120.56, 116.36, 111.80, 55.46 (O CH_3), 40.34 (CH_2) IR: ν_{max} (KBr) cm^{-1}: 3393.6, 3029.6, 294.8, 1695.2, 1515.0, 1406.9, 1277.2, 1131.5, 1026.5, 776.0 HRMS (EI): Found 181.0567 $(M-H)^-$, $C_9H_9O_4$ requires 181.0501.

Synthesis of cinnamic acids **4a, 4c** and phenylpropanoic acids **4b, 4d**

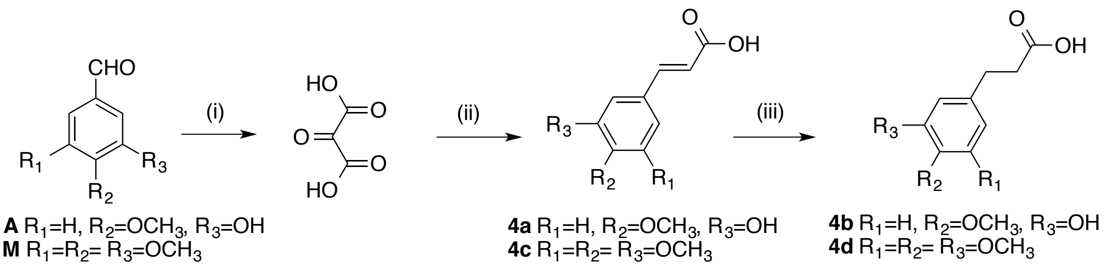

Scheme S2. Synthesis of cinnamic acids **4a, 4c** and propanoic acids **4b, 4d**. Reagents and conditions: (i) Piperidine, acetic acid, Microwave, 8 min. (ii) 12% KOH, Pd/C, H_2, 1h, 20 °C.

Scheme 2: Synthesis of acids **4a-4d**

Reagents and conditions: (i) Piperidine, Acetic acid, 8 min; (ii) KOH 12 %, Pd/C, 1 h, RT.

(*E/Z*)-3-(3-Hydroxy-4-methoxyphenyl)acrylic acid **4a**

A mixture of 3-hydroxy-4-methoxybenzaldehyde **A** (6234 mg, 4.1 mmol), malonic acid (1.706 g, 16.4 mmol), piperidine (1.395 g, 1.62 mL, 16.4 mmol) and acetic acid (2.5 mL) was added to a 5 mL microwave vial. The tube was shaken well and irradiated under microwave for 20 min (150 W, 130 ^{o}C). The cooled mixture was poured into ice-cold water (20 mL) and extracted with ethyl acetate (3 × 20 mL). The organic layer was washed with saturated sodium chloride and dried over sodium sulfate. The solvent was evaporated under reduced pressure to obtain a brown resin which was purified *via* flash chromatography on silica gel (*n*-Hexane:EtOAc, 2:1) to afford the product as a white solid, (72%), Mp. 233-234 °C. ^{1}H-NMR 400MHz (DMSO-d_6):12.20 (br-s, 1H, COOH), 9.19 (br-s, 1H, OH), 7.47-7.43 (d, 1H, 16Hz, C=CH), 7.08 (s, 2H, Ar-H), 6.95-6.93 (d, 1H, 8Hz, Ar-H), 6.27-6.23 (d, 1H, 16Hz, C=CH), 3.80 (s, 3H, O CH_3). ^{13}C-NMR 100MHz (DMSO-d_6): 167.79 (COOH), 149.83, 146.64, 144.21, 127.04, 121.00, 116.20, 114.06, 111.92, 55.59 (OCH_3). IR: ν_{max} (KBr) cm^{-1}: 3394.86, 2940.68, 1703.60, 1519.51, 1445.77, 1276.98. HRMS (EI): Found 193.0561 $(M-H)^{-}$, $C_{10}H_9O_4$ requires 193.0501.

3-(3-Hydroxy-4-methoxyphenyl)propanoic acid **4b**

The acid **4a** (200 mg) was dissolved in EtOAc (20 mL); to this was added 10% Pd/C. The mixture was allowed to stir for 1 h under a hydrogen atmosphere and monitored by TLC until all the starting material had reacted. The reaction mixture was filtered through celite. The solvent was evaporated under reduced pressure to obtain the product as a white solid (162 mg, 80%), Mp. 146-150 °C, which did not require further purification. ^{1}H-NMR 400MHz (DMSO-d_6): 12.08 (br-s, 1H, COOH), 8.82 (br-s, 1H, OH), 6.79 (d, 1H, 8Hz, Ar-H), 6.63 (s, 1H, Ar-H), 6.58 (d, 1H, 9Hz, Ar-H), 3.72 (s, 3H, O CH_3), 2.67 (t, 2H, 7.5Hz, CH_2), 2.44 (t, 2H, 7.5Hz, CH_2). ^{13}C-NMR 100MHz (DMSO-d_6): 173.86 (COOH), 146.26, 145.91, 133.45, 118.59, 118.59, 115.61, 112.23, 55.64 (O CH_3), 35.54 (CH_2), 29.72 (CH_2). IR: ν_{max} (KBr) cm^{-1}: 3350 (OH), 1700 (COOH). HRMS (EI): Found 195.0726 $(M-H)^-$, $C_{10}H_{11}O_4$ requires 195.0657.

(*E/Z*)-3-(3,4,5-Trimethoxyphenyl)acrylic acid **4c**

A mixture of 3,4,5-trimethoxybenzaldehyde **M** (804 mg ,4.1 mmol), malonic acid (1.706 g, 16.4 mmol), piperidine (1.395 g, 1.62 mL, 16.4 mmol) and acetic acid (2.5 mL) was added to a 5 mL microwave vial. The tube was shaken well and irradiated under microwave 8 min (150 W, 130 ^{o}C). The cooled mixture was poured into ice-cold water (20 mL) and extracted with ethyl acetate (3 × 20 mL). The organic layer was washed with saturated sodium chloride and dried over sodium sulfate. The solvent was evaporated under reduced pressure to obtain a brown resin which was purified *via* flash chromatography on silica gel (*n*-Hexane:EtOAc, 2:1) to afford the product as a white solid (956 mg, 84%), Mp.: 126-127 °C. ^{1}H-NMR 400MHz ($CDCl_3$): δ 7.74-7.70 (d, 1H, *J*= 5.5Hz, C=CH), 6.80 (s, 2H, Ar-H), 6.39-6.35 (d, 1H, 5.5Hz, C=CH), 3.92 (s, 9H, OCH_3). ^{13}C-NMR 100MHz ($CDCl_3$): δ 171.90 (COOH), 153.00, 146.65, 129.02, 115.97, 105.03, 60.55 (OCH_3), 55.71(OCH_3). IR: ν_{max} (KBr) cm^{-1}: 2945.74, 1719.94, 1687.73, 1629.03, 1584.20, 1416.49, 1285.90, 1123.54, 998.07. HRMS (EI): Found 261.0752 $(M+Na)^+$, $C_{12}H_{14}O_5Na$ requires 261.0739.

3-(3,4,5-Trimethoxyphenyl)propanoic acid **4d**

Compound **4c** (200 mg) was dissolved in EtOAc (20 mL); to this was added 10% Pd/C. The mixture was allowed to stir for 1 h under a hydrogen atmosphere and monitored by TLC until all the starting material had reacted. The reaction mixture was filtered

through celite and evaporated under reduced pressure to obtain the product as a white solid (142 mg, 65%), Mp. 103-104 °C which did not require further purification. ^{1}H-NMR 400MHz ($CDCl_3$): 6.68 (s, 2H, Ar-H), 3.78 (s, 6H, OCH_3), 3.65 (s, 3H, OCH_3), 2.69 (t, 2H, J=7.5Hz, CH_2), 2.47 (t, 2H, J=7.5Hz, CH_2). ^{13}C-NMR 100MHz ($CDCl_3$): 171.36 (COOH), 145.62, 132.85, 118.39, 118.78, 115.61, 111.23, 56.88(OCH_3), 55.64 (OCH_3), 34.74 (CH_2), 28.22 (CH_2). IR: ν_{max} (KBr) cm^{-1}: 2921.03, 2854.23, 1698.42, 1584.11, 1526.41, 1473.69, 1430.85, 1253.94, 1135.37. HRMS (EI): Found 263.0752 $(M+Na)^+$, $C_{12}H_{16}O_5Na$ requires. 263.0895.
